# Genome-wide DNA Methylation Profiling in Lyme Neuroborreliosis Reveals Altered Methylation Patterns of *HLA* Genes

**DOI:** 10.1093/infdis/jiad451

**Published:** 2023-10-12

**Authors:** Anna J Henningsson, Sandra Hellberg, Maria Lerm, Shumaila Sayyab

**Affiliations:** Division of Clinical Microbiology, Department of Laboratory Medicine, County Hospital Ryhov, Jönköping; Division of Inflammation and Infection, Department of Biomedical and Clinical Sciences, Linköping University, Linköping, Sweden; Division of Inflammation and Infection, Department of Biomedical and Clinical Sciences, Linköping University, Linköping, Sweden; Division of Inflammation and Infection, Department of Biomedical and Clinical Sciences, Linköping University, Linköping, Sweden; Division of Inflammation and Infection, Department of Biomedical and Clinical Sciences, Linköping University, Linköping, Sweden

**Keywords:** Lyme neuroborreliosis, *Borrelia burgdorferi*, DNA methylation, Illumina Infinium methylation EPIC 850K, HLA

## Abstract

Lyme neuroborreliosis (LNB) is a complex neuroinflammatory disorder caused by *Borrelia burgdorferi*, which is transmitted through tick bites. Epigenetic alterations, specifically DNA methylation (DNAm), could play a role in the host immune response during infection. In this study, we present the first genome-wide analysis of DNAm in peripheral blood mononuclear cells from patients with LNB and those without LNB. Using a network-based approach, we highlighted *HLA* genes at the core of these DNAm changes, which were found to be enriched in immune-related pathways. These findings shed light on the role of epigenetic modifications in the LNB pathogenesis that should be confirmed and further expanded upon in future studies.

## Teaser

We present the first genome-wide analysis of DNA methylation in peripheral blood mononuclear cells from patients with and those without the tick-borne infection Lyme neuroborreliosis (LNB). Our findings show that LNB is associated with immune-related DNA methylation changes.

Lyme neuroborreliosis (LNB), a complex inflammatory disorder affecting the nervous system, is caused by spirochetes belonging to the *Borrelia burgdorferi* sensu lato complex that are transmitted to humans via tick bites [1]. The pathogenetic mechanisms are currently only partly understood and may be influenced by both host- and pathogen-related factors.

During infection, host cells undergo intricate transcriptional reprogramming where epigenetic alterations are involved in regulating the expression of immune-related genes that play a central role in the immune response against invading pathogens [2]. While DNA methylation (DNAm), the most extensively studied epigenetic modification, was previously considered quite stable, it is becoming increasingly clear that DNAm changes can occur relatively quickly in response to a changing environment. Indeed, accumulating evidence indicates that exposure to pathogens can alter the DNAm patterns in host immune cells [3–6], which could promote host immunity or help pathogens evade the immune system. Furthermore, epigenetic changes can persist after infection and lead to epigenetic memory or “imprinting” on the genome and could possibly have consequences for long-term disease [7]. Understanding the complex interplay between DNAm, immune responses, and bacterial infections is crucial for identifying new diagnostic tools and treatment strategies, which is highly needed for LNB, especially in patients with slow recovery after antibiotic treatment.

Here we present the first genome-wide DNAm analysis of peripheral blood mononuclear cells (PBMCs) in patients with LNB and patients without LNB (hereafter “non-LNB”).

## METHODS

Patients >18 years of age referred for suspected LNB to the Department of Infectious Diseases, County Hospital Ryhov, Sweden, were prospectively included in the study after their informed consent. Blood samples, serum, cerebrospinal fluid (CSF), and clinical data were collected according to a standardized protocol. Serum and CSF were analyzed for the presence of *Borrelia*-specific antibodies using the IDEIA Lyme Neuroborreliosis immunoglobulin G (IgG) and immunoglobulin M (IgM) test (Oxoid, Hampshire, United Kingdom) and the recomBead Borrelia IgG assay (Mikrogen GmbH). The patients were classified according to the European Federation of the Neurological Sociates guidelines [8]. In this study, 7 patients were included from the group fulfilling the criteria for definite LNB: CSF pleocytosis (mononuclear leukocytes >5 × 10^6^/L) and positive intrathecal antibody index according to the IDEIA and/or recomBead assays. Seven patients without CSF pleocytosis and no *Borrelia*-specific antibodies detected in serum or CSF were included from the group of patients classified as non-LNB. Clinical characteristics of the included patients are presented in Supplementary Tables 1 and 2. The study was approved by the Ethical Review Board in Linköping (M106-04, 2011/65-32, 2015/192-32, 2018/388-32, 2019-02449).

PBMCs were isolated from blood samples collected in BD Vacutainer CPT tubes with sodium heparin (BD Biosciences, Franklin Lakes, New Jersey) according to the instructions provided by the manufacturer and stored at −140°C. After thawing, DNA was isolated using the QIAamp DNA Blood Mini Kit (Qiagen, Hilden, Germany). Genome-wide methylation changes were analyzed using the Illumina Infinium Methylation EPIC 850K BeadChip array (Illumina, San Diego, California) as per the manufacturer's instructions. DNAm analysis was performed by the core facility for Bioinformatics and Expression Analysis at Karolinska Institute, Stockholm, Sweden. Preprocessing of the raw DNAm profiles was performed, including (1) filtering and removal of probes located close to single-nucleotide polymorphisms and probes located on the X and Y chromosomes; (2) quality control; (3) normalization using the subset-quantile within array normalization method; and (4) batch correction (see Supplementary Materials and Methods). Differentially methylated CpGs (DMCs) were defined as probes having a nominal *P* < .05 along with a mean methylation difference (MMD) of >0.2, and a gene was considered as differentially methylated (DMG) if it contained at least 1 DMC. To identify the most functionally interconnected genes, the DMGs were used as input to identify network modules based on the protein–protein interaction network (PPI) using MODifieR [9].

## RESULTS

**Figure 1.**
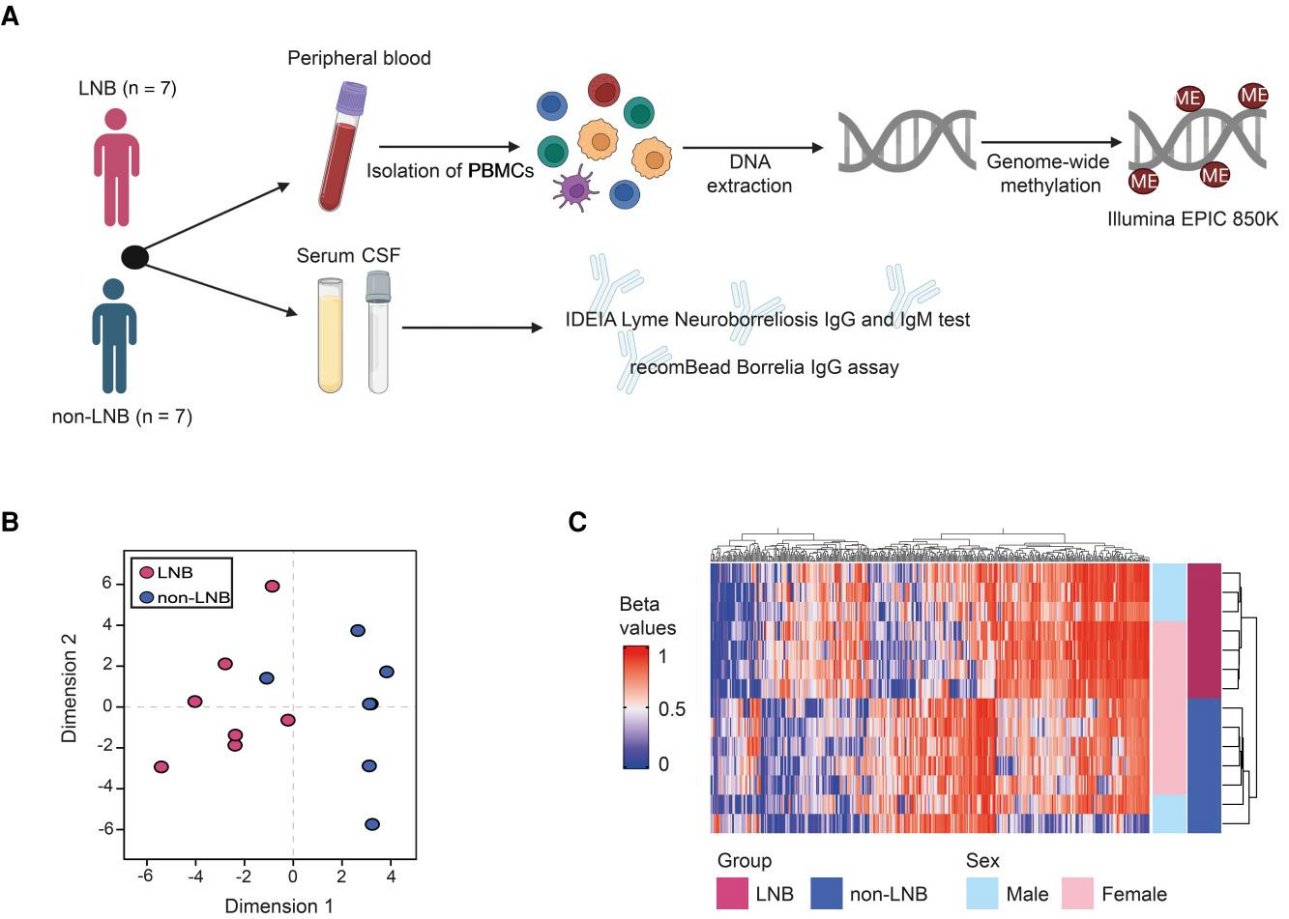
DNA methylation analysis of Lyme neuroborreliosis (LNB). *A*, Peripheral blood mononuclear cells were isolated from people with LNB (n = 7) and people without LNB (non-LNB; n = 7). DNA was extracted and genome-wide profiling of DNA methylation was performed using the Illumina EPIC array covering 850K CpG sites across the genome. Serum and cerebrospinal fluid samples were analyzed for the presence of *Borrelia*-specific antibodies used for the classification of LNB and non-LNB. *B*, Multidimensional scaling plot of the top 1000 most variable CpG positions. *C*, Heatmap of the differentially methylated CpGs (DMCs). DMCs were defined as having a nominal *P* < .05 and a mean methylation difference of >0.2. The non-LNB sample that clustered with LNB patients was removed from the downstream analysis due to ongoing virosis but has been maintained in the figure for illustrative purposes only. Abbreviations: CSF, cerebrospinal fluid; IgG, immunoglobulin G; IgM, immunoglobulin M; LNB, Lyme neuroborreliosis; ME, methylation; PBMCs, peripheral blood mononuclear cells.

To investigate epigenetic alterations during LNB, we performed genome-wide DNAm profiling in PBMCs derived from patients with LNB and non-LNB (Figure 1*A*). Initial unsupervised clustering using multidimensional scaling showed that LNB and non-LNB clustered separately, except for 1 individual in the non-LNB group that clustered together with the LNB patients (Figure 1*B*). Upon further examination of the medical records, this non-LNB patient had shown signs of ongoing virosis at inclusion based on their symptoms (headache, fever, fatigue, and myalgia), moderately elevated C-reactive protein, and normal leukocyte counts. The patient was negative for CSF pleocytosis and *Borrelia* antibodies (IgM and IgG) in serum and CSF, ruling out LNB as a cause of the symptoms. Furthermore, serology was negative for tick-borne encephalitis (TBE) and anaplasmosis. Polymerase chain reaction analyses performed on plasma were negative for *B burgdorferi* sensu lato, *Borrelia miyamotoi*, TBE virus, *Anaplasma phagocytophilum*, *Rickettsia* spp, *Neoehrlichia mikurensis*, and *Babesia* spp. This patient was excluded from the downstream analysis.

Differential methylation analysis showed 428 CpGs to be differentially methylated between LNB and non-LNB patients (nominal *P* < .05 with MMD >0.2; Supplementary Figure 1). Of these, 256 CpGs (59.8%) were hypermethylated and the remaining 172 CpGs were hypomethylated (40.2%; Supplementary Figure 1). This LNB DNAm signature could distinguish LNB patients from non-LNB (Figure 1*C*), suggesting that infection with *B burgdorferi* alters the epigenetic profile in PBMCs.

**Figure 2.**
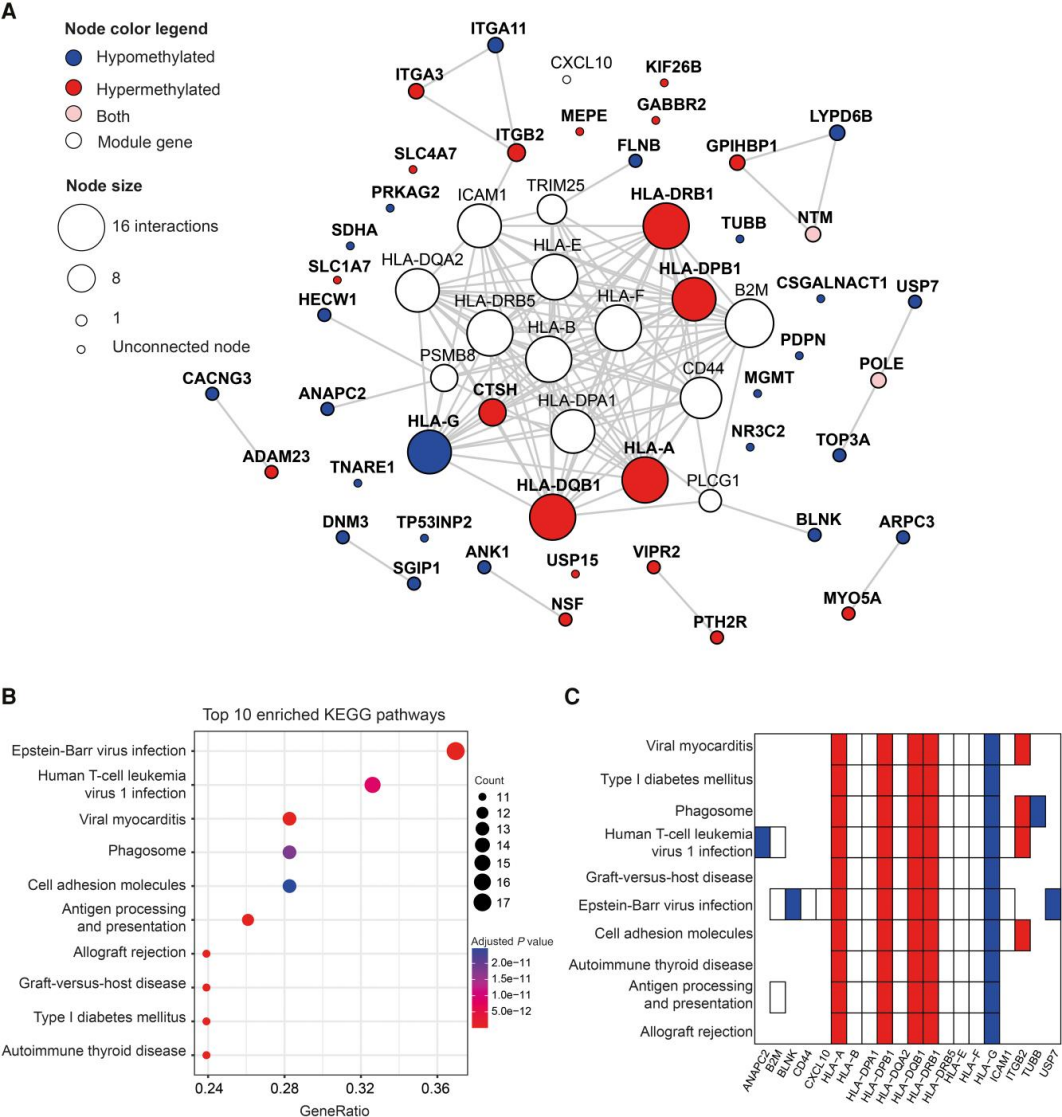
Network module of methylation changes associated with Lyme neuroborreliosis (LNB). *A*, Graphical illustration of the LNB consensus module. The module inference methods MCODE and DIAMOnD were used to infer methylation modules and overlapped to produce the LNB consensus module. Nodes represents genes and the connecting edges show the protein–protein interactions (PPIs). Blue nodes correspond to genes containing only hypomethylated differentially methylated CpGs (DMCs) and red nodes indicate only hypermethylated DMCs that were differentially methylated in the original data (nominal *P* < .05 and mean methylation difference >0.2). Pink nodes contain both hypo- and hypermethylated differentially methylated genes (DMGs). White nodes represent genes added in the module that was not part of the original data. Node size corresponds to the number of interactions in the PPI network. The interactions were chosen from the STRING database with a threshold of >900. *B*, Dot plot of the top 10 most significantly enriched KEGG pathways based on the genes derived from the module. The x-axis represents the gene ratio, dot size represents gene count, and dot color represents adjusted *P* values. A pathway was considered significantly enriched with an adjusted *P* < .05. *C*, Heat plot of the genes annotated to the top 10 most significantly enriched KEGG pathways. Blue denotes hypomethylated and red hypermethylated DMGs, respectively.

The DMCs were annotated to their respective genes, resulting in a total of 248 unique DMGs (Supplementary Table 3). Disease-related proteins have been shown to cluster together in the PPI network [10], creating modules of interconnected proteins belonging to the same biological processes. We found that the DMGs were connected by 1.76 times more interactions than expected (*P* = .0002) using the STRING database PPI network (version 11.5, confidence score threshold >700), suggesting a functional relationship. To further explore this functional relationship and identify more functionally related genes, we employed a network-based modular approach using the DMGs as input. We used the module inference methods MCODE and DIAMOnD as implemented in the R software package MODifieR [9] to construct LNB disease modules. The genes that were identified as present in both methods (MCODE: n = 2886 genes; DIAMOnD: n = 303 genes) were included in the final consensus LNB module, comprising a total of 57 genes (Supplementary Table 4). We found that 44 (77%) of the module genes originated from the original set of DMGs, whereas the remaining 13 genes were derived from the interaction network (Figure 2*A*). Of these 44 genes, 19 genes were hypermethylated (*­*eg, *HLA-A*, *HLA-DPB1*, *HLA-DQB1*, *HLA-DRB1*, *ITGA3*) and 23 genes were hypomethylated (eg, *HLA-G*, *USP7*). The consensus LNB module showed an overall enrichment of KEGG pathways related to viral infections, autoimmune diseases, and antigen presentation (Figure 2*B*). Not surprisingly, the *HLA* genes were mostly involved in driving the enrichment of these pathways (Figure 2*C*, Supplementary Table 5). The *HLA* genes also constituted a central part of the consensus LNB module (Figure 2*A*), where the *HLA* genes that were also differentially methylated had the highest number of connections in the consensus module (n = 14–15 connections). Furthermore, the CpGs associated to the *HLA* genes showed clear difference in methylation status between LNB and non-LNB (Supplementary Figure 2).

## DISCUSSION

LNB is a challenging disease with a poorly defined pathogenesis, which constitutes a significant limitation for the development of more effective diagnostic approaches and for identification and treatment of patients at increased risk for a prolonged disease course. While bacterial infections have previously been associated with changes in DNAm levels, there are, to our knowledge, no studies so far on this in relation to *Borrelia* infection. In this brief report, we present unique DNAm data that shows that the DNA methylome of circulating immune cells is altered in patients with LNB.

A network-based approach was used to search for functionally related genes among the 248 DMGs and their most interconnected proteins in the PPI network, a strategy that has been successfully applied to identify disease genes and biomarkers [10, 11]. The resulting LNB network module highlighted *HLA* genes to be among the most central genes, and the presence of *HLA* genes dominated among the top enriched pathways. Certain HLA class II alleles have been shown to correlate strongly with Lyme borreliosis disease severity and to be involved in promoting antibody-responsive or antibody-refractory Lyme arthritis [12, 13]. However, there are contradictory results regarding HLA and *B burgdorferi* where studies report both up-regulated and down-regulated expression of HLA class II on antigen-presenting cells [14, 15] in response to *B burgdorferi*. We found that 3 of the 4 HLA class II alleles that were differentially methylated were hypermethylated in LNB as compared to non-LNB patients. The dominance of *HLA* genes and many of the pathways that were enriched are generally associated with immune responses to infections; thus, it needs to be further clarified if this is a general immune response or specific to *Borrelia* infection. A previous study showed that down-regulation of *HLA* genes was only observed in response to *B burgdorferi* infection and not observed in response to various other pathogens [14], which could suggest that this could be more specific to the *Borrelia* infection. However, it should be noted that the DMCs and pathways are very broad and HLA in particular plays a central role in the response to infection, which is a potential limitation of the study. A follow-up study of these DNAm patterns in people with other central nervous system (CNS) conditions would be very interesting and provide further knowledge on the specificity of these LNB-associated DNAm changes.

The small number of samples included in this exploratory study is a potential limitation and also limits the number of DMCs that survived the strict threshold imposed by correcting for multiple hypothesis testing. To mitigate this issue, we performed a network-based analysis [9] to identify functionally interacting genes, which have a more significant biological impact as compared to single gene analysis. Further studies with increased sample sizes are needed to corroborate and extend the current findings. Another limitation is that we only included samples taken shortly after *Borrelia* infection and since, for example, several HLA-DRB1 alleles have been associated to the development of chronic manifestations of Lyme arthritis, it would be highly relevant to investigate if the observed DNAm changes persist in individuals who develop more persisting symptoms of LNB.

While LNB is a disease pertained to the CNS, we have studied changes in peripheral immune cells. Indeed, potentially more disease-relevant changes could be identified in tissues closer to the target organ. However, from a clinical perspective, lumbar puncture is more cumbersome and causes the patient more discomfort than a blood sample, and identifying disease-associated changes in a more easily accessible specimen than CSF would be very valuable. Since this is the first study of its kind, there are no comparable studies for validation of our findings in PBMCs, but the fact that DNAm changes can be readily captured in blood holds potential for future studies.

In summary, this study is the first of its kind and provides compelling evidence of a role for DNAm in the pathogenesis of LNB. The results presented in this pilot study provide a foundation for the discovery of new biomarkers for LNB and generate new hypotheses that can deepen the understanding of important disease mechanisms.

## Supplementary Data

Supplementary materials are available at *The Journal of Infectious Diseases* online. Consisting of data provided by the authors to benefit the reader, the posted materials are not copyedited and are the sole responsibility of the authors, so questions or comments should be addressed to the corresponding author.

## Supplementary Material

jiad451_Supplementary_Data

## Notes

***Acknowledgments.*** We especially acknowledge Paula Gyllemark at the Department of Infectious Diseases at County Hospital Ryhov, Jönköping, for assistance in collection of the clinical data; Malin Larger, Department of Laboratory Medicine, County Hospital Ryhov, Jönköping, for help with the DNA extraction; the staff at the Department of Infectious Diseases and the Department of Laboratory Medicine, County Hospital Ryhov, Jönköping, for sampling and sample handling; and Annabel Burkhard, Department of Biomedical and Clinical Sciences, Linköping University, for the initial bioinformatics analysis. We additionally acknowledge the Core Facility at Novum Bioinformatics and Expression Analysis (BEA), and the BEA at the Karolinska Institute, Stockholm, Sweden, for performing the Illumina Infinium Methylation EPIC 850K array. We also acknowledge the Swedish National Infrastructure for Computing at the National Supercomputing Centre, Linköping University, for enabling the data handling, partially funded by the Swedish Research Council (grant number 2018-05973). Figure 1 was created using biorender.com.

***Author contributions.*** A. J. H. was responsible for the collection of the patient material and compilation of clinical data. S. H. and S. S. performed bioinformatics analysis and interpretation. A. J. H., M. L., and S. S. were involved in the overall design of the study. S. S. and S. H. prepared figures and tables. S. H. drafted the manuscript. All authors were involved in reading and revising the manuscript prior to submission, and all authors approved the submitted version.

***Financial support.*** This work was supported by the Swedish Society of Medicine (grant number 784721); the Medical Research Council of Southeast Sweden (grant number 852511); and Futurum–the Academy of Health and Welfare, Region Jönköping County. Funding to pay the Open Access publication charges for this article was provided by Linköping University.

## References

[1] Ford L, Tufts DM. Lyme neuroborreliosis: mechanisms of *B. burgdorferi* infection of the nervous system. Brain Sci 2021; 11:789. 34203671 10.3390/brainsci11060789 PMC8232152

[2] Qin W, Scicluna BP, van der Poll T. The role of host cell DNA methylation in the immune response to bacterial infection. Front Immunol 2021; 12:696280. 34394088 10.3389/fimmu.2021.696280 PMC8358789

[3] Huoman J, Sayyab S, Apostolou E, et al Epigenetic rewiring of pathways related to odour perception in immune cells exposed to SARS-CoV-2 in vivo and in vitro. Epigenetics 2022; 17:1875–91. 35758003 10.1080/15592294.2022.2089471 PMC9665140

[4] Pacis A, Tailleux L, Morin AM, et al Bacterial infection remodels the DNA methylation landscape of human dendritic cells. Genome Res 2015; 25:1801–11. 26392366 10.1101/gr.192005.115 PMC4665002

[5] Mukherjee S, Vipat VC, Chakrabarti AK. Infection with influenza A viruses causes changes in promoter DNA methylation of inflammatory genes. Influenza Other Respir Viruses 2013; 7:979–86. 23758996 10.1111/irv.12127 PMC4634256

[6] Karlsson L, Das J, Nilsson M, et al A differential DNA methylome signature of pulmonary immune cells from individuals converting to latent tuberculosis infection. Sci Rep 2021; 11:19418. 34593857 10.1038/s41598-021-98542-3 PMC8484443

[7] Nikesjo F, Sayyab S, Karlsson L, et al Defining post-acute COVID-19 syndrome (PACS) by an epigenetic biosignature in peripheral blood mononuclear cells. Clin Epigenetics 2022; 14:172. 36517875 10.1186/s13148-022-01398-1 PMC9748378

[8] Mygland A, Ljostad U, Fingerle V, et al EFNS guidelines on the diagnosis and management of European Lyme neuroborreliosis. Eur J Neurol 2010; 17:8–16, e1–4. 19930447 10.1111/j.1468-1331.2009.02862.x

[9] de Weerd HA, Badam TVS, Martinez-Enguita D, et al MODifier: an ensemble R package for inference of disease modules from transcriptomics networks. Bioinformatics 2020; 36:3918–9. 32271876 10.1093/bioinformatics/btaa235

[10] Menche J, Sharma A, Kitsak M, et al Disease networks. Uncovering disease-disease relationships through the incomplete interactome. Science 2015; 347:1257601. 25700523 10.1126/science.1257601 PMC4435741

[11] Hellberg S, Eklund D, Gawel DR, et al Dynamic response genes in CD4^+^ T cells reveal a network of interactive proteins that classifies disease activity in multiple sclerosis. Cell Rep 2016; 16:2928–39. 27626663 10.1016/j.celrep.2016.08.036

[12] Iliopoulou BP, Guerau-de-Arellano M, Huber BT. HLA-DR alleles determine responsiveness to *Borrelia burgdorferi* antigens in a mouse model of self-perpetuating arthritis. Arthritis Rheum 2009; 60:3831–40. 19950279 10.1002/art.25005 PMC2828865

[13] Steere AC, Klitz W, Drouin EE, et al Antibiotic-refractory Lyme arthritis is associated with HLA-DR molecules that bind a *Borrelia burgdorferi* peptide. J Exp Med 2006; 203:961–71. 16585267 10.1084/jem.20052471 PMC3212725

[14] Brouwer MAE, Jones-Warner W, Rahman S, et al *B. burgdorferi* sensu lato-induced inhibition of antigen presentation is mediated by RIP1 signaling resulting in impaired functional T cell responses towards *Candida albicans*. Ticks Tick Borne Dis 2021; 12:101611. 33360386 10.1016/j.ttbdis.2020.101611

[15] Gutierrez-Hoffmann MG, O'Meally RN, Cole RN, Tiniakou E, Darrah E, Soloski MJ. *Borrelia burgdorferi*–induced changes in the class II self-immunopeptidome displayed on HLA-DR molecules expressed by dendritic cells. Front Med 2020; 7:568. 10.3389/fmed.2020.00568 PMC7524959 33043033

