# Supplementary material for: Genome-wide DNA Methylation Profiling in Lyme Neuroborreliosis Reveals Altered Methylation Patterns of HLA Genes
Source: J Infect Dis. 2023 Oct 12;229(4):1209–14. doi: 10.1093/infdis/jiad451 (PMC11011177; doi:10.1093/infdis/jiad451)
Supplement: jiad451_Supplementary_Data [file jiad451_supplementary_data.zip › Supplementary_Material_JID_clean.docx]

**Supplementary materials and methods**

**Cohort description**

All participants gave written informed consent before participating in the study and the study was approved by the Ethical Review Board in Linköping (M106-04, 2011/65-32, 2015/192-32, 2018/388-32, 2019-02449). Detailed information about the subjects included in the study can be found in **Table S1** and **Table S2.** Initial descriptive analysis of demographic variables was performed on the available information about age, gender. Continuous variables were compared using an unpaired two-tailed t-test and categorical variables were examined using the Pearson χ2 test or Fisher’s exact test (if the number of observations was smaller than five).

***Isolation of peripheral blood mononuclear cells***

Whole blood was collected in BD Vacutainer^®^ CPT™ tubes with Sodium Heparin^N^ (BD Bioscience, Franklin Lakes, NJ, USA) and the PBMCs were isolated according to the protocol provided by the manufacturer. The cells were resuspended in freezing medium consisting of 10% dimethyl sulfoxide (Sigma-Aldrich, Saint Louis, MO, USA) and 90% heat-inactivated fetal bovine serum (Sigma-Aldrich) supplemented with Penicillin-Streptomycin (Sigma-Aldrich). The cells were stored at -80^○^C in a Mr.Frosty™ Freezing Container (Thermo Fisher Scientific, Waltham, MA, USA) and subsequently transferred to -140°C and stored until further use.

***Extraction of DNA and DNA methylation analysis***

Cells were thawed and DNA was extracted from PBMC using the QIAamp DNA Blood Mini Kit (Qiagen, Hilden, Germany) according to the instructions provided by the manufacturer using the spin protocol. The quality and concentration of the DNA was determined using a Nanodrop^TM^ Spectrophotometer (Thermo Scientific^TM^). Genome-wide DNA methylation changes were analyzed using the Illumina Infinium Methylation EPIC 850K BeadChip array (Illumina Inc, San Diego, USA) as per the manufacturer’s instructions. Analysis was carried out at the core facility for Bioinformatics and Expression Analysis at Karolinska Institute, Stockholm, Sweden.

***Pre-processing of DNA methylation***

The resulting raw IDAT-files containing the raw DNA methylation profiles were analysed in R (v. 4.0.2) using the minfi package (v. 1.36.0) [1] and the data were pre-processed in several steps. The following filters were applied: i) removal of probes with detection p-values above 0.001, ii) removal of non-CpG probes, iii) removal of multi-hit probes, iv) removal of all probes in X and Y chromosomes. We removed the sex chromosomes from our data set, as female X-inactivation skews the distribution of β values. Of the initial 865,918 probes, 809,691 probes remained upon filtering. After filtering, quality control was performed, and normalisation of the data was done with subset-quantile within array (SWAN) normalisation method [2]. The β values and M values of the samples were calculated against each probe per sample. The quality of the data was assessed before and after the normalisation. Thereafter, we performed singular value decomposition (SVD) analysis using the ChAMP package (version 2.19.3) to identify underlying components of variation within the filtered and normalised data set [3]. Corrections were performed for slide and array using ComBat from the SVA package (version 3.38.0) [4]. To investigate any inherent differences between the two groups (LNB *versus* non-LNB), multidimensional scaling (MDS) using the Euclidean distances between the samples for the 1000 most variable CpGs was used. To account for a potential influence of cell heterogeneity on the observed methylation changes, we utilized the Houseman method to infer the cell type proportions within the samples. [5]. We observed no differences in the cell proportions in individuals with LNB compared to non-LNB (data not shown).

***Differential DNA methylation analysis***

To identify differentially methylated CpGs between LNB and non-LNB, we performed differential DNA methylation analysis using the limma package (v. 3.46.0) [6]. A linear model was fitted to the filtered, normalised and SVD-corrected DNA methylation data. For each investigated probe, moderated t-statistics, log2 Fold Change (logFC) and p-values were computed. The logFC values represent the average β methylation difference (referred to as mean methylation difference, MMD) between the LNB *versus* non-LNB. Differentially methylated CpGs (DMCs) were defined as probes having a nominal p-value < 0.05 along with an MMD of > 0.2. The distribution of the DMCs among all investigated DNA methylation sites were illustrated by heatmaps using ComplexHeatmap package in R [7]. The DMCs were mapped to genes using the annotation file provided by Illumina. A differentially methylated gene (DMG) was defined as having at least one DMC and were considered hyper- or hypomethylated if all DMCs within the gene were hyper- or hypomethylated, respectively. If both hyper- and hypomethylated genes were present in the same gene, the gene was considered having a mixed methylation pattern (**Supplementary Table S3**).

***Module identification***

To identify the most functionally related genes among the DMGs (n=248; comparing LNB with non-LNB), disease modules were inferred using MCODE [8] and DIAMoND [9] from the R package MODifieR (version 0.1.3) [10]. We used the human protein-protein interaction (PPI) network from the STRING database (version 11) [11], including only interactions with a combined evidence score of at least 700. From the resulting modules, a consensus module was derived comprising the intersection of the module genes identified by MCODE (n=2,886 genes) and DIAMoND (n=303). The consensus module consisted of 57 genes out of which 44 genes were part of the seed DMGs. The consensus module was visualized using Cytoscape (version 3.8.2) [12], using a threshold of score of at least 900 for the PPI. The genes derived from each module method can be found in **Supplementary Table S4**.

***Pathway enrichment analysis***

Pathway enrichment analysis, based on KEGG, was performed using the function *enrichKEGG* from clusterProfiler (version 4.7.1.002) [13] (**Supplementary Table S5**). The results were corrected for the background (*i.e.,* all measured genes with the inclusion of the PPI network genes when testing module genes), and the pathways with an adjusted p-value ≤ 0.05 were considered significant. Visualization of the top 10 most enriched pathways was with *dotplot* and *heatplot*.

**References**

1. Aryee MJ, Jaffe AE, Corrada-Bravo H, et al. Minfi: a flexible and comprehensive Bioconductor package for the analysis of Infinium DNA methylation microarrays. Bioinformatics **2014**; 30:1363-9.

2. Maksimovic J, Gordon L, Oshlack A. SWAN: Subset-quantile within array normalization for illumina infinium HumanMethylation450 BeadChips. Genome Biol **2012**; 13:R44.

3. Morris TJ, Butcher LM, Feber A, et al. ChAMP: 450k Chip Analysis Methylation Pipeline. Bioinformatics **2014**; 30:428-30.

4. Leek JT, Johnson WE, Parker HS, Jaffe AE, Storey JD. The sva package for removing batch effects and other unwanted variation in high-throughput experiments. Bioinformatics **2012**; 28:882-3.

5. Houseman EA, Accomando WP, Koestler DC, et al. DNA methylation arrays as surrogate measures of cell mixture distribution. BMC Bioinformatics **2012**; 13:86.

6. Ritchie ME, Phipson B, Wu D, et al. limma powers differential expression analyses for RNA-sequencing and microarray studies. Nucleic Acids Res **2015**; 43:e47.

7. Gu Z, Eils R, Schlesner M. Complex heatmaps reveal patterns and correlations in multidimensional genomic data. Bioinformatics **2016**; 32:2847-9.

8. Bader GD, Hogue CW. An automated method for finding molecular complexes in large protein interaction networks. BMC Bioinformatics **2003**; 4:2.

9. Ghiassian SD, Menche J, Barabasi AL. A DIseAse MOdule Detection (DIAMOnD) algorithm derived from a systematic analysis of connectivity patterns of disease proteins in the human interactome. PLoS Comput Biol **2015**; 11:e1004120.

10. de Weerd HA, Badam TVS, Martinez-Enguita D, et al. MODifieR: an Ensemble R Package for Inference of Disease Modules from Transcriptomics Networks. Bioinformatics **2020**; 36:3918-9.

11. Szklarczyk D, Gable AL, Lyon D, et al. STRING v11: protein-protein association networks with increased coverage, supporting functional discovery in genome-wide experimental datasets. Nucleic Acids Res **2019**; 47:D607-D13.

12. Shannon P, Markiel A, Ozier O, et al. Cytoscape: a software environment for integrated models of biomolecular interaction networks. Genome Res **2003**; 13:2498-504.

13. Yu G, Wang LG, Han Y, He QY. clusterProfiler: an R package for comparing biological themes among gene clusters. OMICS **2012**; 16:284-7.

**Supplementary Figures**

**
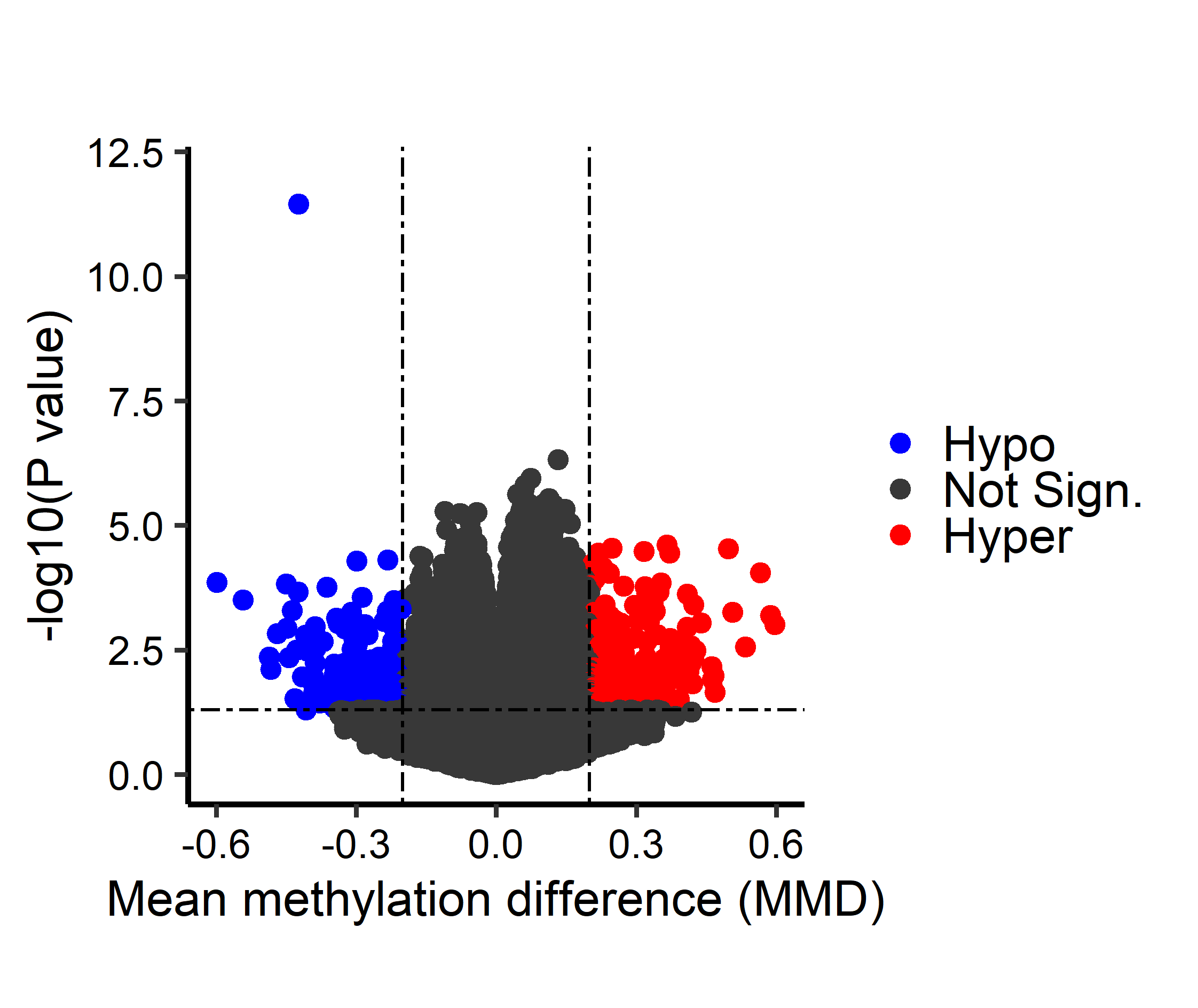
**

**Figure S1. Differentially methylated CpGs between LNB and non-LNB.** Volcano plot of -log10(P value) against the mean methylation difference (MMD) comparing patients with Lyme neuroborreliosis (LNB) as compared to patients without LNB (non-LNB). Threshold for differentially methylated CpGs were nominal p-value < 0.05 and a MMD > 0.2 and are indicated in the figure. Red dots denote significant hypermethylated CpGs (n=256) and blue denotes significant hypomethylated CpGs (n=172).

**
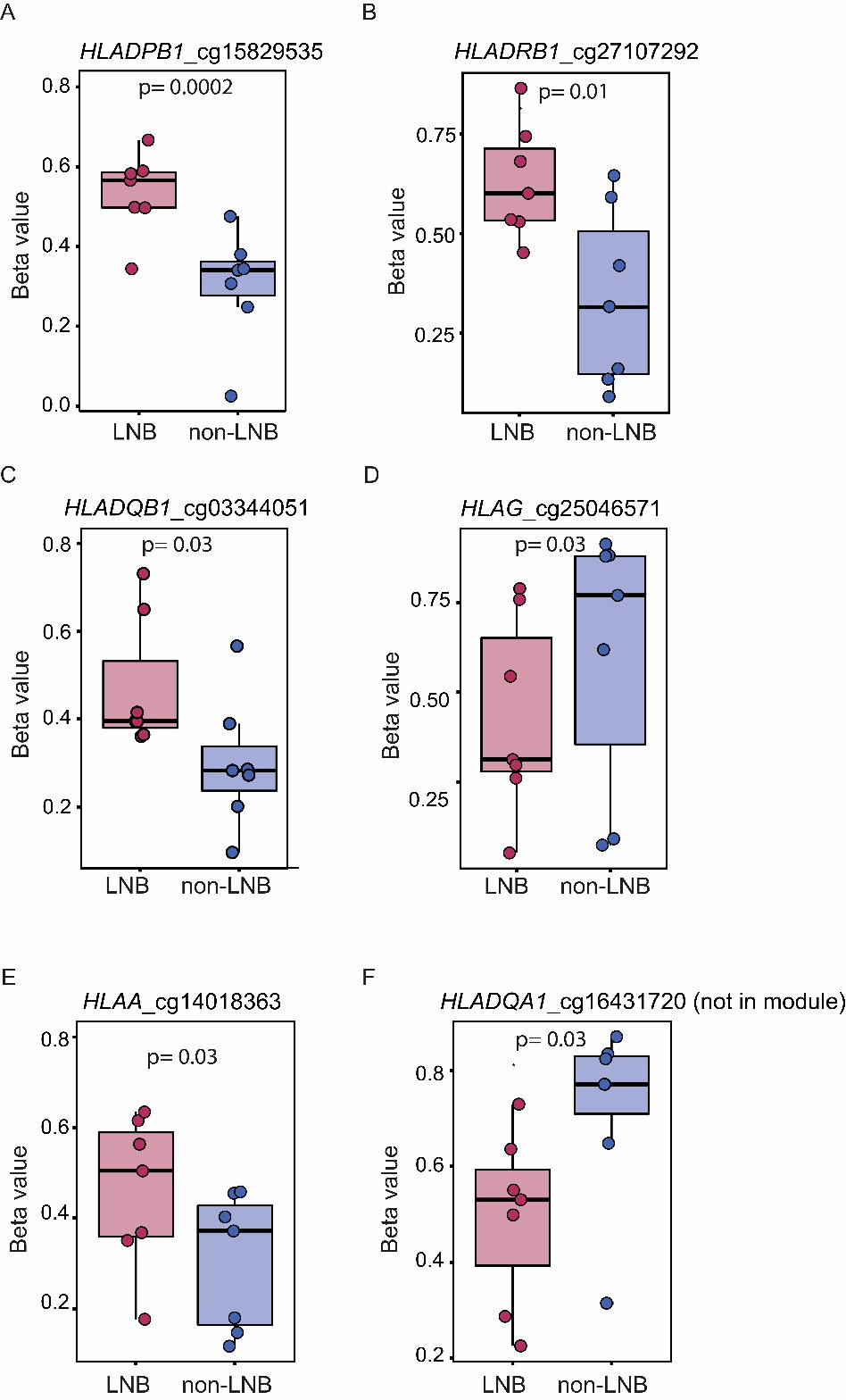
**

**Figure S2. DNA methylation status of HLA-associated CpGs.** (A-F) Boxplots of β values (y-axis) showing the DNA methylation for the 6 HLA-associated CpGs present among the differentially methylated CpGs (p<0.05 and mean methylation difference > 0.2) comparing patients with Lyme neuroborreliosis (LNB) and patients without LNB (non-LNB). The individual p values for each comparison are shown in the figure.

**Supplementary Data**

Supplementary information and data are available at *The Journal of Infectious Diseases* online. The methylation data is available at the GeneExpression Omnibus GSE232841**.** The data consists of filtered and pre-processed DNA methylation data from the de-identified individual samples in the study. The code used to generate the results in the present study is available at Github ([https://github.com/Lerm-Lab/LNB](https://eur01.safelinks.protection.outlook.com/?url=https%3A%2F%2Fgithub.com%2FLerm-Lab%2FLNB&data=05%7C01%7Csandra.hellberg%40liu.se%7C6c3ede45074446c87ed008db61a836a4%7C913f18ec7f264c5fa816784fe9a58edd%7C0%7C0%7C638211147312183158%7CUnknown%7CTWFpbGZsb3d8eyJWIjoiMC4wLjAwMDAiLCJQIjoiV2luMzIiLCJBTiI6Ik1haWwiLCJXVCI6Mn0%3D%7C3000%7C%7C%7C&sdata=cGTnwoUagX1xD3DSqNP7oCdtoJHNKYUIlywte8%2FIA0Y%3D&reserved=0)).
