# Supplementary material for: Genome-wide DNA Methylation Profiling in Lyme Neuroborreliosis Reveals Altered Methylation Patterns of HLA Genes
Source: J Infect Dis. 2023 Oct 12;229(4):1209–14. doi: 10.1093/infdis/jiad451 (PMC11011177; doi:10.1093/infdis/jiad451)
Supplement: jiad451_Supplementary_Data [file jiad451_supplementary_data.zip › Table S1.docx]

| **Table S1. Clinical characteristics of the cohort** | |  |
| --- | --- | --- |
|  | LNB | non-LNB |
| ***n*** | 7 | 7 |
| **Age, years (mean±SD)** | 48±16 | 51±21 |
| **Sex (f/m)** | 4/3 | 5/2 |
| **Previous Lyme Borreliosis** | 0/7 | 1/6 |
| **CSF mononuclear leukocytes x10^6^/L (mean±SD)** | 249±139 | 1±0 |
| **CSF/serum albumin index (mean±SD)** | 18.7±6.3 | 6.5±0.9 |
| **Borrelia IgG (pos/neg)** |  |  |
| *serum* | 6/1 | 0/7 |
| *CSF* | 6/1 | 0/7 |
| **Borrelia IgM (pos/neg)** |  |  |
| *serum* | 4/3 | 0/7 |
| *CSF* | 4/3 | 0/7 |
| **Symptoms (yes/no)** |  |  |
| *Headache* | 6/1 | 2/5 |
| *Fatigue* | 7/0 | 1/6 |
| *Fever* | 1/6 | 1/6 |
| *Neck pain* | 5/2 | 0/7 |
| *Poor apetite* | 3/4 | 1/6 |
| *Nasuea* | 1/6 | 1/6 |
| *Concentration difficulties* | 1/7 | 0/7 |
| *Radiculitis* | 5/2 | 0/7 |
| *Myalgia/arthralgia* | 4/3 | 1/6 |
| *Paresthesias* | 6/1 | 0/7 |
| *Vertigo* | 1/6 | 0/7 |
| *Facial palsy* | 4/3 | 0/7 |
| *CSF= cerebrospinal fluid, LNB = Lyme neuroborreliosis* |  |  |
