# Supplementary material for: Genome-wide DNA Methylation Profiling in Lyme Neuroborreliosis Reveals Altered Methylation Patterns of HLA Genes
Source: J Infect Dis. 2023 Oct 12;229(4):1209–14. doi: 10.1093/infdis/jiad451 (PMC11011177; doi:10.1093/infdis/jiad451)
Supplement: jiad451_Supplementary_Data [file jiad451_supplementary_data.zip › Table S2.docx]

| **LNB patient No.** | **Age (years)** | **Sex (f/m)** | **Duration of symptoms (days)** | **CSF mononuclear cells  (x 10 ^6^/L))** | ***Borrelia*-specific IgM and IgG AI** | **Head-ache (y/n)** | **Fatigue (y/n)** | **Fever (y/n)** | **Neck pain (y/n)** | **Poor apetite (y/n)** | **Nausea (y/n)** | **Concen-tration difficulties (y/n)** | **Radiculitis (y/n)** | **Myalgia/ arthralgia (y/n)** | **Vertigo (y/n)** | **Facial palsy (y/n)** |
| --- | --- | --- | --- | --- | --- | --- | --- | --- | --- | --- | --- | --- | --- | --- | --- | --- |
| 1 | 58 | m | 2 | 26 | IgG pos IgM neg | y | y | n | y | n | n | n | y | y | n | n |
| 2 | 52 | f | 28 | 48 | IgG pos IgM neg | y | y | n | n | n | n | n | y | n | n | n |
| 3 | 69 | m | 6 | 14 | IgG neg IgM pos | n | y | n | n | n | n | n | n | n | n | y |
| 4 | 60 | f | 14 | 1030 | IgG neg IgM pos | y | y | n | y | y | n | y | y | y | y | y |
| 5 | 25 | m | 2 | 172 | IgG pos IgM pos | y | y | n | y | y | y | n | n | n | n | y |
| 6 | 33 | f | 28 | 62 | IgG pos IgM pos | y | y | n | y | n | n | n | y | y | n | y |
| 7 | 40 | m | 28 | 390 | IgG pos IgM pos | y | y | y | y | n | n | n | y | y | n | n |

**Supplementary table S2. Clinical characteristics of individual Lyme neuroborreliosis patients.**

LNB: Lyme neuroborreliosis. f: female. m: male. CSF: cerebrospinal fluid. AI: CSF/serum antibody index. pos: positive. neg: negative. y: yes. n: no.
