# Supplementary material for: Genome-wide DNA Methylation Profiling in Lyme Neuroborreliosis Reveals Altered Methylation Patterns of HLA Genes
Source: J Infect Dis. 2023 Oct 12;229(4):1209–14. doi: 10.1093/infdis/jiad451 (PMC11011177; doi:10.1093/infdis/jiad451)
Supplement: jiad451_Supplementary_Data [file jiad451_supplementary_data.zip › Table S3.docx]

| **Table S3.** Differentially methylated CpGs between LNB and non-LNB. Nominal p-value <0.05 and mean methylation difference > 0.2 | | | | |
| --- | --- | --- | --- | --- |
| **CpG ID** | **SYMBOL** | **logFC** | **P.Value** | **adj.P.Val** |
| cg02945674 | ABCA1 | 0,277145 | 0,024138 | 0,553769 |
| cg01045938 | ACADSB | -0,32063 | 0,019998 | 0,53929 |
| cg01802772 | ACOT11 | 0,250798 | 0,019806 | 0,538233 |
| cg10318771 | ADAM23 | 0,230634 | 0,033186 | 0,576985 |
| cg16398051 | ADAMTS17 | 0,27385 | 0,018877 | 0,535235 |
| cg05079227 | ADAMTS17 | -0,20329 | 0,039652 | 0,591603 |
| cg21211688 | ADAMTSL2 | -0,4859 | 0,004413 | 0,435925 |
| cg05709581 | ADGRD1 | 0,272973 | 0,041583 | 0,595337 |
| cg00413734 | AEBP1 | -0,4237 | 3,50E-12 | 2,96E-06 |
| cg17830280 | AGBL1 | 0,217207 | 0,001405 | 0,376859 |
| cg06742252 | AGTPBP1 | 0,213395 | 0,03466 | 0,580439 |
| cg16081854 | AHRR | 0,402907 | 0,009021 | 0,479712 |
| cg16675581 | AKR7A2 | 0,285557 | 0,015431 | 0,519597 |
| cg06843575 | ALMS1 | -0,23218 | 0,023244 | 0,550604 |
| cg09307883 | ANAPC2 | -0,21651 | 0,039213 | 0,59056 |
| cg08537289 | ANG | 0,245436 | 0,027883 | 0,563807 |
| cg22256607 | ANK1 | -0,28988 | 0,009681 | 0,48375 |
| cg02658985 | APBB2 | -0,30946 | 0,049532 | 0,609707 |
| cg21117207 | ARHGEF19 | 0,217389 | 0,009694 | 0,483794 |
| cg12206353 | ARHGEF28 | 0,291456 | 0,010447 | 0,489412 |
| cg23603995 | ARID1B | 0,416163 | 0,002837 | 0,413442 |
| cg10738648 | ARPC3 | -0,30331 | 0,035141 | 0,581472 |
| cg24576051 | ATP10A | 0,249151 | 0,007097 | 0,463493 |
| cg16032134 | ATP10A | 0,236686 | 0,011996 | 0,499544 |
| cg17784027 | ATP6V0D2 | 0,250237 | 0,014039 | 0,511239 |
| cg15147473 | BACH2 | 0,358542 | 0,004848 | 0,439623 |
| cg00713204 | BANP | 0,259965 | 0,017811 | 0,529743 |
| cg04506342 | BAZ2B | 0,255167 | 0,027957 | 0,564126 |
| cg03389755 | BCAR3 | -0,24049 | 0,010137 | 0,487732 |
| cg00905457 | BLNK | -0,32462 | 0,01363 | 0,509041 |
| cg21864016 | C14orf132 | 0,353505 | 0,000143 | 0,293049 |
| cg18709904 | C14orf182 | -0,42488 | 0,000217 | 0,321208 |
| cg18874114 | C16orf46 | -0,26023 | 0,005626 | 0,448637 |
| cg01892977 | C1QTNF8 | 0,208217 | 0,033556 | 0,577569 |
| cg11803132 | C8orf37-AS1 | 0,319088 | 0,000168 | 0,306405 |
| cg11074323 | CACNG3 | -0,39131 | 0,011155 | 0,494145 |
| cg01126931 | CAMK1D | 0,209909 | 0,011707 | 0,497801 |
| cg20189467 | CCDC144A | 0,359444 | 0,020407 | 0,540886 |
| cg04612030 | CCDC154 | 0,278365 | 0,016559 | 0,524095 |
| cg00236261 | CCDC3 | -0,21765 | 0,008573 | 0,476322 |
| cg24284539 | CCDC3 | -0,31129 | 0,01021 | 0,488399 |
| cg27653615 | CCZ1 | -0,4373 | 0,000511 | 0,341471 |
| cg23611477 | CDK11A | -0,22262 | 0,00106 | 0,360436 |
| cg26400546 | CDK18 | 0,210419 | 0,007212 | 0,464174 |
| cg09084244 | CDK2AP1 | -0,54227 | 0,000316 | 0,336322 |
| cg09445240 | CHIA | -0,26195 | 0,037514 | 0,587056 |
| cg21823080 | CNTNAP2 | -0,25454 | 0,0068 | 0,460386 |
| cg17296678 | COL13A1 | 0,365976 | 2,47E-05 | 0,272742 |
| cg16995742 | COPS8 | -0,3033 | 0,043135 | 0,597833 |
| cg05531409 | CPNE4 | 0,251512 | 0,043825 | 0,599685 |
| cg01565803 | CRP | 0,274738 | 0,036332 | 0,584163 |
| cg07781090 | CSGALNACT1 | -0,27595 | 0,008241 | 0,472976 |
| cg02079756 | CTB-7E3.1 | 0,309752 | 0,038466 | 0,588709 |
| cg01682370 | CTSH | 0,253667 | 0,005234 | 0,444397 |
| cg25044524 | CYTSB | 0,231798 | 0,04432 | 0,600384 |
| cg00218628 | DDX11 | -0,2396 | 0,019381 | 0,537074 |
| cg06354054 | DHX37 | 0,382159 | 0,009079 | 0,479914 |
| cg14506194 | DIRC3 | 0,246551 | 0,000792 | 0,34668 |
| cg27487121 | DMKN | 0,209542 | 0,000558 | 0,341696 |
| cg03040423 | DNAJC8 | 0,247678 | 0,011688 | 0,497702 |
| cg09644135 | DNM3 | -0,20786 | 0,002323 | 0,400939 |
| cg01377456 | DTNA | 0,216493 | 0,041207 | 0,595074 |
| cg18621672 | DYDC1 | 0,33627 | 0,000372 | 0,336322 |
| cg09581911 | DYRK4 | -0,31322 | 0,016737 | 0,524813 |
| cg09829645 | DYSF | -0,28961 | 0,022308 | 0,546773 |
| cg05074631 | DZIP1 | -0,20148 | 0,003067 | 0,414163 |
| cg07466463 | EIF2C2 | 0,344621 | 0,001542 | 0,383078 |
| cg01380346 | ELL | 0,220782 | 0,035602 | 0,582494 |
| cg05130642 | EPPK1 | -0,62053 | 6,37E-05 | 0,282948 |
| cg00929286 | ESRRB | 0,357448 | 0,007611 | 0,466926 |
| cg13953972 | FAM107B | 0,240187 | 0,017658 | 0,529615 |
| cg06124141 | FAM120A | -0,31846 | 0,001238 | 0,367867 |
| cg12914114 | FAM120B | 0,389133 | 0,009884 | 0,48535 |
| cg09209679 | FAM19A5 | -0,40745 | 0,049957 | 0,610738 |
| cg21265404 | FAM75C1 | 0,299846 | 0,000415 | 0,33964 |
| cg05237503 | FBXO3 | -0,31048 | 0,000545 | 0,341471 |
| cg11707219 | FGF17 | -0,24078 | 0,000843 | 0,348904 |
| cg20564724 | FLNB | -0,20392 | 0,000482 | 0,341471 |
| cg18892258 | GABBR2 | 0,23864 | 0,017767 | 0,529743 |
| cg19935756 | GABRB1 | 0,417116 | 0,005838 | 0,451121 |
| cg16463459 | GALNT10 | 0,416701 | 0,002609 | 0,407704 |
| cg03812172 | GCK | 0,25333 | 0,000805 | 0,346983 |
| cg05144129 | GLTPD2 | 0,389341 | 0,002455 | 0,402032 |
| cg23283667 | GPIHBP1 | 0,282321 | 0,006098 | 0,454304 |
| cg11758647 | GPR111 | -0,22952 | 0,016229 | 0,522554 |
| cg15031384 | GPR63 | 0,251589 | 0,002161 | 0,396957 |
| cg10662047 | GRAMD1B | 0,596874 | 0,000969 | 0,357769 |
| cg05519582 | GRAMD4 | -0,34267 | 0,000728 | 0,344737 |
| cg18584561 | GREB1 | -0,20529 | 0,03016 | 0,569618 |
| cg16908938 | GREB1 | -0,28378 | 0,034397 | 0,580003 |
| cg11036359 | HCG4 | -0,28698 | 0,029669 | 0,568352 |
| cg17348244 | HEATR2 | 0,56541 | 8,80E-05 | 0,287019 |
| cg06096382 | HECW1 | -0,34762 | 0,013353 | 0,506706 |
| cg06972843 | HIVEP2 | -0,30932 | 0,003044 | 0,414163 |
| cg14018363 | HLA-A | 0,209562 | 0,028426 | 0,565372 |
| cg15829535 | HLA-DPB1 | 0,272571 | 0,000165 | 0,306405 |
| cg16431720 | HLA-DQA1 | -0,26202 | 0,029412 | 0,567876 |
| cg03344051 | HLA-DQB1 | 0,214072 | 0,023776 | 0,552624 |
| cg27107292 | HLA-DRB1 | 0,313053 | 0,014541 | 0,514446 |
| cg25046571 | HLA-G | -0,34936 | 0,026914 | 0,561906 |
| cg09371091 | HRH1 | -0,23841 | 0,006824 | 0,460386 |
| cg03854098 | HSPA12A | -0,22211 | 0,002073 | 0,393899 |
| cg02890259 | HSPB7 | 0,312618 | 0,044786 | 0,601148 |
| cg20964965 | INPP5A | -0,26367 | 0,021149 | 0,544276 |
| cg26217827 | ITGA11 | -0,44845 | 0,001166 | 0,364103 |
| cg13213536 | ITGA3 | 0,216378 | 0,01286 | 0,503585 |
| cg02464073 | ITGB2 | 0,328508 | 0,006001 | 0,453558 |
| cg17718679 | KCNE3 | -0,21208 | 0,015877 | 0,521602 |
| cg06638795 | KCNG3 | 0,376984 | 0,006818 | 0,460386 |
| cg09737095 | KCNJ5 | 0,206854 | 0,045338 | 0,602349 |
| cg12401798 | KCNQ2 | 0,303511 | 0,000798 | 0,346983 |
| cg05164926 | KCTD11 | -0,20576 | 0,008702 | 0,477194 |
| cg16412745 | KIAA0284 | 0,58709 | 0,000648 | 0,344531 |
| cg05522042 | KIAA0513 | 0,26695 | 0,004166 | 0,430761 |
| cg11461787 | KIAA1614 | 0,334445 | 0,025649 | 0,558576 |
| cg07501029 | KIF26B | 0,409088 | 0,00109 | 0,362249 |
| cg04104977 | L1TD1 | 0,355529 | 0,011543 | 0,49712 |
| cg07796016 | LCE1C | 0,314352 | 0,006167 | 0,454916 |
| cg01275521 | LLGL2 | 0,244191 | 0,009753 | 0,483971 |
| cg08227260 | LMF1 | -0,25896 | 0,004973 | 0,441522 |
| cg15174052 | LMF1 | -0,2182 | 0,015138 | 0,517359 |
| cg14507403 | LMTK2 | 0,21616 | 0,031386 | 0,573164 |
| cg07173352 | LOC100652768 | 0,533212 | 0,002778 | 0,412381 |
| cg09321367 | LOC101927438 | 0,279729 | 0,006733 | 0,459773 |
| cg20187719 | LOC285375 | 0,370635 | 0,039396 | 0,591195 |
| cg05095647 | LOC339975 | 0,381979 | 0,006914 | 0,461521 |
| cg16964206 | LOC401387 | 0,214792 | 0,014454 | 0,514037 |
| cg05288475 | LOC440461 | -0,26929 | 0,017015 | 0,526079 |
| cg22557003 | LPHN3 | -0,22777 | 0,005707 | 0,449825 |
| cg09255886 | LUZP1 | 0,273017 | 0,004766 | 0,438905 |
| cg08133365 | LUZP1 | 0,286227 | 0,010576 | 0,490018 |
| cg16120147 | LYNX1 | 0,231669 | 0,037585 | 0,587271 |
| cg19840088 | LYPD6B | -0,22656 | 0,000839 | 0,347998 |
| cg20950152 | MAP2K6 | 0,264004 | 0,012726 | 0,502521 |
| cg19492498 | MBL2 | 0,321882 | 0,000228 | 0,323411 |
| cg24398793 | MEOX2 | 0,319408 | 0,023471 | 0,551759 |
| cg11379315 | MEPE | 0,217432 | 0,030155 | 0,569618 |
| cg07797740 | MFSD4 | -0,28189 | 0,000986 | 0,358702 |
| cg09993319 | MGMT | -0,29174 | 0,042138 | 0,595902 |
| cg18678645 | MIR886 | 0,228401 | 0,000505 | 0,341471 |
| cg08745965 | MIR886 | 0,211324 | 0,000833 | 0,347384 |
| cg00124993 | MIR886 | 0,265821 | 0,000894 | 0,352802 |
| cg04481923 | MIR886 | 0,232438 | 0,001371 | 0,374031 |
| cg26896946 | MIR886 | 0,254559 | 0,002954 | 0,414163 |
| cg25340688 | MIR886 | 0,255173 | 0,003501 | 0,420642 |
| cg06536614 | MIR886 | 0,274199 | 0,003567 | 0,422672 |
| cg25923457 | MLLT10 | -0,30338 | 0,023549 | 0,552047 |
| cg05392448 | MORN1 | 0,215523 | 0,041205 | 0,595074 |
| cg12466610 | MOSC2 | -0,21216 | 0,033382 | 0,577354 |
| cg26077133 | MSRA | -0,38788 | 0,005683 | 0,449787 |
| cg03370588 | MYO5A | 0,323688 | 0,015542 | 0,520001 |
| cg13324220 | MYOF | -0,4434 | 0,004472 | 0,436317 |
| cg01295646 | MYOM2 | -0,20801 | 0,00261 | 0,407704 |
| cg21847720 | MYOM2 | -0,24019 | 0,004485 | 0,436317 |
| cg17181941 | NCAPH | 0,308061 | 0,01452 | 0,514326 |
| cg07711085 | NKAIN4 | 0,21888 | 0,026646 | 0,561216 |
| cg15295200 | NMNAT3 | 0,387764 | 0,00638 | 0,456817 |
| cg17061760 | NR3C2 | -0,37122 | 0,002122 | 0,395206 |
| cg11595218 | NRXN2 | 0,241043 | 0,049005 | 0,608915 |
| cg08238516 | NSF | 0,21425 | 0,044804 | 0,601174 |
| cg04650544 | NTM | 0,267451 | 0,003656 | 0,423927 |
| cg09455118 | NTM | -0,22405 | 0,02201 | 0,546252 |
| cg16289461 | NXN | 0,252469 | 0,016434 | 0,523714 |
| cg14605795 | OR2T11 | 0,207151 | 5,82E-05 | 0,276791 |
| cg03133799 | OR2T34 | 0,237754 | 0,007388 | 0,465201 |
| cg05298460 | OR4M2 | 0,366175 | 0,004395 | 0,434882 |
| cg04606582 | OR4N3P | 0,291188 | 0,006426 | 0,45699 |
| cg16373817 | PALLD | 0,327295 | 0,000833 | 0,347384 |
| cg21015022 | PARP12 | 0,23501 | 0,04734 | 0,606156 |
| cg27494055 | PCDHA10 | -0,43172 | 0,030161 | 0,56962 |
| cg21566433 | PCSK6 | -0,23301 | 0,027419 | 0,563141 |
| cg14307563 | PDE4D | -0,4154 | 0,010901 | 0,492625 |
| cg16959075 | PDE8A | 0,256967 | 0,006048 | 0,453844 |
| cg02933679 | PDPN | -0,30445 | 0,002053 | 0,393899 |
| cg14281403 | PHC2 | 0,468256 | 0,022568 | 0,548006 |
| cg10666341 | PLA1A | -0,23934 | 0,010508 | 0,489566 |
| cg18816122 | PLEKHG4B | 0,307759 | 0,027814 | 0,563747 |
| cg16855633 | PNMA2 | 0,201493 | 0,009089 | 0,479914 |
| cg20139683 | POLE | -0,63127 | 2,64E-05 | 0,273384 |
| cg14181112 | POLE | 0,232514 | 0,000392 | 0,33774 |
| cg01608425 | POLE | -0,3997 | 0,002967 | 0,414163 |
| cg19723528 | PPIL2 | 0,466133 | 0,010464 | 0,489497 |
| cg10926851 | PPP2R2B | 0,360954 | 0,043921 | 0,599843 |
| cg03958058 | PPP2R2D | 0,215162 | 0,040599 | 0,593594 |
| cg03493520 | PRCP | 0,248731 | 0,003333 | 0,41719 |
| cg02368820 | PRDM16 | -0,3868 | 0,002947 | 0,414163 |
| cg14767338 | PRDM16 | -0,27456 | 0,034304 | 0,579864 |
| cg17611936 | PRKAG2 | -0,21145 | 0,009224 | 0,481068 |
| cg06378142 | PRR12 | -0,38979 | 0,017798 | 0,529743 |
| cg13901316 | PTH2R | 0,241894 | 0,014389 | 0,513764 |
| cg11144103 | PTRF | 0,267843 | 0,018239 | 0,532193 |
| cg12953206 | RAB25 | -0,28041 | 0,022842 | 0,548862 |
| cg09216282 | RFX3-AS1 | 0,34513 | 0,026106 | 0,560327 |
| cg06060754 | RGS14 | -0,33913 | 0,000937 | 0,355568 |
| cg17509989 | RGS14 | -0,38762 | 0,001075 | 0,360859 |
| cg16006841 | RGS14 | -0,29813 | 0,002403 | 0,401864 |
| cg02673002 | RNF169 | -0,21318 | 0,017384 | 0,528219 |
| cg05871851 | RNF213 | 0,279687 | 0,006862 | 0,460611 |
| cg12016309 | RNU6-2 | -0,30858 | 0,027118 | 0,56253 |
| cg04248279 | RPH3AL | -0,28712 | 0,000274 | 0,331114 |
| cg21560722 | SBF2 | 0,281093 | 0,013614 | 0,509012 |
| cg25161252 | SBK2 | 0,241312 | 0,00399 | 0,429985 |
| cg11133658 | SCARA5 | 0,247872 | 0,012555 | 0,502021 |
| cg23490161 | SDHA | -0,22604 | 0,004791 | 0,439287 |
| cg08778598 | SDHAP3 | -0,25083 | 0,004362 | 0,43454 |
| cg14582632 | SERPINB2 | 0,412416 | 0,013905 | 0,510408 |
| cg19596870 | SERPINB6 | -0,34726 | 0,006713 | 0,459661 |
| cg19577958 | SERPINB9 | 0,412096 | 0,008571 | 0,476322 |
| cg07884673 | SFMBT1 | 0,205946 | 0,016487 | 0,524095 |
| cg09364373 | SGIP1 | -0,28864 | 0,01821 | 0,532184 |
| cg19032705 | SH3PXD2B | 0,229083 | 0,011515 | 0,496951 |
| cg09157251 | SHANK2 | -0,29559 | 0,02851 | 0,565518 |
| cg03997626 | SIAH3 | 0,264768 | 0,021523 | 0,54531 |
| cg04924408 | SIAH3 | 0,259275 | 0,025521 | 0,558257 |
| cg11610546 | SLC14A2-AS1 | 0,23808 | 0,03464 | 0,580335 |
| cg17284124 | SLC15A2 | 0,411717 | 0,010599 | 0,490205 |
| cg12146221 | SLC19A1 | 0,46205 | 0,006798 | 0,460386 |
| cg09134876 | SLC1A7 | 0,326583 | 0,011639 | 0,497376 |
| cg25410233 | SLC35F3 | 0,217224 | 0,036906 | 0,585804 |
| cg11104311 | SLC35F3 | 0,213618 | 0,038162 | 0,588257 |
| cg06550894 | SLC39A14 | 0,239436 | 0,006236 | 0,454916 |
| cg00460155 | SLC4A7 | 0,275869 | 0,004941 | 0,441358 |
| cg05873820 | SLC6A6 | 0,273202 | 0,021846 | 0,545974 |
| cg13229487 | SLC7A1 | -0,24679 | 0,038918 | 0,589606 |
| cg13946163 | SLC9A2 | -0,308 | 0,014611 | 0,514771 |
| cg20704148 | SLCO2B1 | 0,367887 | 0,003937 | 0,428826 |
| cg11173002 | SLIT3 | 0,324721 | 0,005837 | 0,451121 |
| cg02111632 | SMCO2 | -0,2981 | 0,041974 | 0,595674 |
| cg11706829 | SMOC1 | 0,372119 | 0,002763 | 0,411932 |
| cg04305804 | SMYD3 | 0,365641 | 0,014312 | 0,513483 |
| cg04798314 | SMYD3 | 0,326863 | 0,019942 | 0,538822 |
| cg16490124 | SNORA14B | 0,208411 | 0,018069 | 0,531377 |
| cg17341969 | SNPH | 0,301243 | 0,033152 | 0,576985 |
| cg16670573 | SNRNP25 | -0,21889 | 0,00032 | 0,336322 |
| cg22802014 | SNRNP40 | 0,321639 | 0,00215 | 0,396957 |
| cg19539986 | SNX6 | -0,39267 | 0,025472 | 0,558174 |
| cg06302877 | SP6 | 0,213067 | 0,002094 | 0,394443 |
| cg13920856 | TANC1 | -0,40387 | 0,003327 | 0,41719 |
| cg02068164 | TARS | -0,27517 | 0,001551 | 0,383191 |
| cg19500155 | TBCD | 0,24358 | 0,000661 | 0,344531 |
| cg13078798 | TGFBR3 | -0,29931 | 0,020213 | 0,540462 |
| cg14549203 | TMC7 | 0,388517 | 0,005685 | 0,449787 |
| cg18756931 | TMCO4 | -0,34645 | 0,006018 | 0,453558 |
| cg05471616 | TMTC1 | 0,422557 | 0,000387 | 0,33774 |
| cg15133953 | TOP3A | -0,28989 | 0,004709 | 0,438806 |
| cg20592836 | TP53INP2 | -0,37807 | 0,036764 | 0,58527 |
| cg03403996 | TRIM31 | -0,35899 | 0,018215 | 0,532193 |
| cg17579624 | TRIOBP | 0,242996 | 0,031865 | 0,574337 |
| cg03047376 | TSNARE1 | -0,23035 | 0,04568 | 0,603036 |
| cg18131458 | TSPAN11 | 0,357901 | 0,015502 | 0,520001 |
| cg21035183 | TTLL6 | 0,264649 | 0,015962 | 0,521639 |
| cg17732597 | TUBB | -0,21331 | 0,035194 | 0,581624 |
| cg01334504 | U2AF1 | -0,23194 | 0,004791 | 0,439287 |
| cg15281606 | UBE2F | -0,26727 | 0,004929 | 0,441358 |
| cg08880261 | UMODL1 | -0,30072 | 0,003329 | 0,41719 |
| cg10146442 | UNC84A | -0,29904 | 5,20E-05 | 0,276791 |
| cg08871399 | USP15 | 0,261413 | 0,025409 | 0,557877 |
| cg01891583 | USP7 | -0,46937 | 0,001475 | 0,381613 |
| cg25909532 | VIPR2 | 0,296177 | 0,000396 | 0,33774 |
| cg07456585 | VSX2 | -0,29623 | 0,00579 | 0,450928 |
| cg05865331 | WDR20 | 0,236968 | 0,043924 | 0,599843 |
| cg04156365 | WDR72 | 0,270995 | 0,030244 | 0,570032 |
| cg14741147 | WFDC6 | -0,20648 | 0,031379 | 0,573144 |
| cg23765831 | WIPF3 | 0,247378 | 2,89E-05 | 0,276492 |
| cg12995384 | ZBTB2 | 0,228778 | 6,94E-05 | 0,285624 |
| cg05867245 | ZBTB46 | 0,217433 | 0,042675 | 0,596971 |
| cg03885735 | ZNF365 | -0,21426 | 0,03836 | 0,58855 |
| cg17880750 | ZNF484 | 0,348899 | 0,000216 | 0,321208 |
| cg02078039 | ZNRD1 | -0,23883 | 0,00834 | 0,474118 |
| cg25728685 | ZSCAN12 | 0,238624 | 0,006529 | 0,457739 |
| cg02956194 |  | 0,496973 | 2,90E-05 | 0,276492 |
| cg13574174 |  | 0,316457 | 3,30E-05 | 0,276791 |
| cg02770728 |  | 0,218801 | 3,58E-05 | 0,276791 |
| cg01282508 |  | 0,371515 | 3,61E-05 | 0,276791 |
| cg12300724 |  | -0,23281 | 4,92E-05 | 0,276791 |
| cg22190077 |  | 0,241633 | 9,15E-05 | 0,287019 |
| cg00855458 |  | 0,212608 | 0,000117 | 0,287019 |
| cg08914944 |  | -0,59857 | 0,000139 | 0,291664 |
| cg05792312 |  | -0,44949 | 0,000148 | 0,297177 |
| cg26816491 |  | 0,203023 | 0,000161 | 0,306405 |
| cg09935224 |  | -0,36278 | 0,000172 | 0,309108 |
| cg07176285 |  | 0,409219 | 0,000237 | 0,324629 |
| cg16547127 |  | 0,315781 | 0,000439 | 0,340759 |
| cg02602925 |  | -0,23391 | 0,000521 | 0,341471 |
| cg00193551 |  | 0,340485 | 0,000521 | 0,341471 |
| cg12219587 |  | 0,506306 | 0,00055 | 0,341509 |
| cg10773128 |  | 0,438609 | 0,000901 | 0,353293 |
| cg16978449 |  | 0,241613 | 0,00116 | 0,364103 |
| cg18025438 |  | -0,32446 | 0,001193 | 0,365457 |
| cg00986626 |  | 0,21586 | 0,001219 | 0,366886 |
| cg00324979 |  | 0,250985 | 0,001253 | 0,368786 |
| cg25279747 |  | 0,264792 | 0,001325 | 0,371764 |
| cg04398163 |  | 0,208121 | 0,00142 | 0,378135 |
| cg03635442 |  | -0,21487 | 0,001541 | 0,383078 |
| cg23052585 |  | -0,4089 | 0,001589 | 0,384757 |
| cg15517438 |  | -0,40839 | 0,001686 | 0,386739 |
| cg00145055 |  | 0,297173 | 0,001768 | 0,388666 |
| cg17661798 |  | 0,293119 | 0,001834 | 0,389808 |
| cg02096220 |  | 0,372653 | 0,001843 | 0,390132 |
| cg11608150 |  | 0,230273 | 0,002304 | 0,400541 |
| cg27112983 |  | 0,251834 | 0,00246 | 0,402283 |
| cg07158503 |  | 0,221343 | 0,0026 | 0,407691 |
| cg08164151 |  | -0,20916 | 0,002638 | 0,407943 |
| cg17920809 |  | -0,22237 | 0,002765 | 0,411943 |
| cg06736229 |  | 0,253571 | 0,002992 | 0,414163 |
| cg06405219 |  | -0,4284 | 0,003166 | 0,416121 |
| cg24906015 |  | 0,426768 | 0,003234 | 0,41719 |
| cg06777732 |  | -0,22237 | 0,003367 | 0,417445 |
| cg12308308 |  | 0,423226 | 0,00448 | 0,436317 |
| cg24358762 |  | 0,321938 | 0,004499 | 0,436732 |
| cg12770425 |  | -0,23685 | 0,004918 | 0,441285 |
| cg02118671 |  | 0,226117 | 0,005062 | 0,443114 |
| cg12540243 |  | 0,315922 | 0,005075 | 0,443201 |
| cg23885472 |  | 0,230099 | 0,005108 | 0,443598 |
| cg25960393 |  | 0,240608 | 0,005467 | 0,446534 |
| cg25673075 |  | -0,32574 | 0,005952 | 0,452594 |
| cg26387458 |  | 0,269918 | 0,006218 | 0,454916 |
| cg25254350 |  | 0,294279 | 0,006485 | 0,45754 |
| cg24711482 |  | 0,247492 | 0,006923 | 0,461655 |
| cg20627512 |  | -0,21278 | 0,007122 | 0,463619 |
| cg01502301 |  | 0,2127 | 0,007332 | 0,464772 |
| cg03930924 |  | 0,354921 | 0,007603 | 0,466905 |
| cg05971102 |  | -0,48324 | 0,007678 | 0,467258 |
| cg16373765 |  | 0,271348 | 0,007769 | 0,467905 |
| cg11733135 |  | 0,300233 | 0,007926 | 0,469755 |
| cg25951717 |  | -0,27248 | 0,008986 | 0,479435 |
| cg05472380 |  | 0,267518 | 0,009143 | 0,480487 |
| cg18359218 |  | 0,386086 | 0,009481 | 0,482724 |
| cg05123933 |  | -0,23612 | 0,009508 | 0,482724 |
| cg07564979 |  | -0,31363 | 0,009534 | 0,482806 |
| cg02177141 |  | -0,30542 | 0,009643 | 0,483422 |
| cg15828613 |  | -0,34728 | 0,009655 | 0,483518 |
| cg01433855 |  | 0,215458 | 0,009733 | 0,483929 |
| cg21974656 |  | 0,255107 | 0,010002 | 0,487019 |
| cg08049519 |  | 0,309437 | 0,010721 | 0,490936 |
| cg08961558 |  | 0,271529 | 0,011119 | 0,494145 |
| cg01451645 |  | 0,272165 | 0,011166 | 0,494292 |
| cg17215151 |  | 0,299075 | 0,011304 | 0,495524 |
| cg23513018 |  | -0,39372 | 0,011326 | 0,495733 |
| cg00147627 |  | -0,21312 | 0,011384 | 0,496201 |
| cg02533724 |  | 0,46259 | 0,012472 | 0,50162 |
| cg08024471 |  | -0,33505 | 0,012493 | 0,501712 |
| cg01245604 |  | 0,398628 | 0,012836 | 0,503544 |
| cg15877769 |  | 0,270629 | 0,013831 | 0,510112 |
| cg09673061 |  | 0,200672 | 0,013917 | 0,510499 |
| cg17726767 |  | 0,36435 | 0,014611 | 0,514771 |
| cg01436125 |  | 0,420165 | 0,014871 | 0,51588 |
| cg20236856 |  | 0,202586 | 0,014913 | 0,516165 |
| cg27395310 |  | -0,26748 | 0,015415 | 0,519588 |
| cg04889166 |  | 0,346156 | 0,015631 | 0,520602 |
| cg01236565 |  | -0,3086 | 0,015704 | 0,520768 |
| cg00777622 |  | 0,272123 | 0,015859 | 0,521602 |
| cg20778915 |  | -0,21616 | 0,016615 | 0,524241 |
| cg21193212 |  | 0,258998 | 0,016996 | 0,525938 |
| cg17758652 |  | -0,32734 | 0,017525 | 0,528783 |
| cg03747858 |  | 0,217425 | 0,017544 | 0,528867 |
| cg19214707 |  | -0,27936 | 0,017927 | 0,530235 |
| cg04319046 |  | 0,331991 | 0,018595 | 0,53377 |
| cg06746449 |  | 0,284523 | 0,018763 | 0,534866 |
| cg10523200 |  | -0,32018 | 0,019144 | 0,536069 |
| cg10681981 |  | -0,34576 | 0,019688 | 0,537778 |
| cg11842073 |  | 0,235567 | 0,019747 | 0,537906 |
| cg08197665 |  | 0,332458 | 0,021042 | 0,543908 |
| cg00718752 |  | 0,342667 | 0,021148 | 0,544276 |
| cg16729283 |  | 0,202902 | 0,021446 | 0,545041 |
| cg21790413 |  | -0,21861 | 0,021544 | 0,545463 |
| cg26237810 |  | 0,218816 | 0,021715 | 0,545618 |
| cg00463901 |  | 0,233492 | 0,022017 | 0,546252 |
| cg08516846 |  | 0,22794 | 0,022034 | 0,546266 |
| cg14375582 |  | -0,31917 | 0,022692 | 0,548187 |
| cg13320413 |  | 0,300831 | 0,02302 | 0,549438 |
| cg17779733 |  | -0,21535 | 0,023819 | 0,55271 |
| cg08651389 |  | 0,202138 | 0,024109 | 0,553664 |
| cg22304519 |  | 0,318987 | 0,024824 | 0,555835 |
| cg15279541 |  | -0,24554 | 0,024997 | 0,556477 |
| cg22807110 |  | 0,223406 | 0,02506 | 0,556917 |
| cg20673407 |  | -0,28349 | 0,026196 | 0,560453 |
| cg10619365 |  | 0,246502 | 0,026403 | 0,560837 |
| cg10942914 |  | -0,24769 | 0,027094 | 0,562484 |
| cg24245216 |  | 0,28779 | 0,027393 | 0,563128 |
| cg25904344 |  | -0,20324 | 0,027852 | 0,563747 |
| cg08736526 |  | -0,35049 | 0,028641 | 0,565699 |
| cg02427933 |  | -0,21353 | 0,02951 | 0,568122 |
| cg00967012 |  | 0,289692 | 0,029628 | 0,568312 |
| cg15852849 |  | 0,244234 | 0,0299 | 0,56919 |
| cg04986567 |  | -0,20871 | 0,030039 | 0,569424 |
| cg09422696 |  | -0,23312 | 0,030198 | 0,569856 |
| cg10463108 |  | 0,392337 | 0,030674 | 0,571111 |
| cg14271106 |  | -0,31142 | 0,031974 | 0,574633 |
| cg19225953 |  | 0,34671 | 0,032011 | 0,574745 |
| cg06394109 |  | 0,21649 | 0,032013 | 0,574745 |
| cg12293347 |  | 0,234776 | 0,032195 | 0,575647 |
| cg06031234 |  | 0,334508 | 0,03246 | 0,57571 |
| cg04608203 |  | -0,29739 | 0,032971 | 0,576497 |
| cg12381370 |  | -0,31571 | 0,034507 | 0,580003 |
| cg11314779 |  | 0,221135 | 0,034587 | 0,580146 |
| cg21444500 |  | 0,207375 | 0,034785 | 0,580583 |
| cg02120552 |  | -0,34115 | 0,035317 | 0,581792 |
| cg15483637 |  | 0,284856 | 0,035572 | 0,582396 |
| cg12333628 |  | -0,28509 | 0,035761 | 0,58262 |
| cg14241748 |  | 0,334312 | 0,036383 | 0,584385 |
| cg11878555 |  | -0,21324 | 0,037205 | 0,586398 |
| cg04875706 |  | 0,332467 | 0,0373 | 0,586498 |
| cg06002867 |  | -0,35222 | 0,038034 | 0,587853 |
| cg00474373 |  | -0,24015 | 0,038486 | 0,58876 |
| cg10266977 |  | -0,26023 | 0,039113 | 0,590192 |
| cg06060457 |  | -0,23824 | 0,039251 | 0,59072 |
| cg24065597 |  | 0,279322 | 0,039674 | 0,591603 |
| cg09434726 |  | -0,20854 | 0,039852 | 0,592104 |
| cg16069065 |  | -0,31355 | 0,041572 | 0,595337 |
| cg15111296 |  | -0,23816 | 0,041924 | 0,595662 |
| cg12156950 |  | 0,223909 | 0,042125 | 0,595869 |
| cg26567385 |  | 0,243207 | 0,042459 | 0,596545 |
| cg02877261 |  | 0,292104 | 0,042594 | 0,596747 |
| cg13604933 |  | 0,305513 | 0,043072 | 0,597713 |
| cg08146708 |  | 0,319908 | 0,04318 | 0,597901 |
| cg10811640 |  | -0,34917 | 0,043387 | 0,598705 |
| cg17920646 |  | 0,216249 | 0,043406 | 0,598728 |
| cg14716990 |  | -0,21373 | 0,044655 | 0,601061 |
| cg16089727 |  | 0,22818 | 0,045052 | 0,601669 |
| cg10486833 |  | 0,208298 | 0,045161 | 0,60192 |
| cg02157626 |  | -0,22061 | 0,045487 | 0,602631 |
| cg00017157 |  | -0,33781 | 0,04588 | 0,603249 |
| cg16523141 |  | -0,3462 | 0,046487 | 0,604654 |
| cg02100397 |  | 0,317373 | 0,046779 | 0,605023 |
| cg07686394 |  | -0,24225 | 0,046863 | 0,605198 |
| cg11420142 |  | 0,360646 | 0,048201 | 0,607375 |
| cg04540199 |  | 0,300691 | 0,048642 | 0,608128 |
