# Supplementary material for: Genome-wide DNA Methylation Profiling in Lyme Neuroborreliosis Reveals Altered Methylation Patterns of HLA Genes
Source: J Infect Dis. 2023 Oct 12;229(4):1209–14. doi: 10.1093/infdis/jiad451 (PMC11011177; doi:10.1093/infdis/jiad451)
Supplement: jiad451_Supplementary_Data [file jiad451_supplementary_data.zip › Table S4.docx]

| **Table S4.** Genes identified using the module inference methods MCODE, DIAMoND. The consensus module was derived by intersecting the MCODE and DIAMoND modules. Yes= in module, x= not in the module. | | | |
| --- | --- | --- | --- |
| **SYMBOL** | **MCODE (n=2886)** | **DIAMoND (n=303)** | **Consensus (n=57)** |
| AAK1 | Yes | x | x |
| AANAT | Yes | x | x |
| ABCA1 | x | Yes | x |
| ABCC6 | Yes | x | x |
| ABCE1 | Yes | x | x |
| ABL1 | Yes | x | x |
| ACAA1 | Yes | x | x |
| ACAD9 | Yes | x | x |
| ACADSB | x | Yes | x |
| ACAP1 | Yes | x | x |
| ACAP2 | Yes | x | x |
| ACKR3 | Yes | x | x |
| ACLY | Yes | x | x |
| ACO2 | Yes | x | x |
| ACOT11 | x | Yes | x |
| ACSM3 | Yes | x | x |
| ACTN1 | Yes | x | x |
| ACTN4 | Yes | x | x |
| ACTR10 | Yes | x | x |
| ACTR1A | Yes | x | x |
| ACTR2 | Yes | x | x |
| ACTR3 | Yes | x | x |
| ACY1 | Yes | x | x |
| ADA2 | Yes | x | x |
| ADAM10 | Yes | x | x |
| ADAM22 | Yes | x | x |
| ADAMTS17 | x | Yes | x |
| ADAMTS7 | Yes | x | x |
| ADAMTSL2 | x | Yes | x |
| ADAR | x | Yes | x |
| ADCY1 | Yes | x | x |
| ADCY2 | Yes | x | x |
| ADCY3 | Yes | x | x |
| ADCY4 | Yes | x | x |
| ADCY5 | Yes | x | x |
| ADCY6 | Yes | x | x |
| ADCY7 | Yes | x | x |
| ADCY8 | Yes | x | x |
| ADCY9 | Yes | x | x |
| ADCYAP1 | Yes | x | x |
| ADCYAP1R1 | Yes | x | x |
| ADGRA1 | Yes | x | x |
| ADGRD1 | x | Yes | x |
| ADGRL1 | Yes | x | x |
| ADGRL2 | Yes | x | x |
| ADGRL3 | Yes | x | x |
| ADGRV1 | Yes | x | x |
| ADI1 | Yes | x | x |
| ADM | Yes | x | x |
| ADM2 | Yes | x | x |
| ADORA1 | Yes | x | x |
| ADORA2B | Yes | x | x |
| ADRA2A | Yes | x | x |
| ADRA2B | Yes | x | x |
| ADRA2C | Yes | x | x |
| ADRB1 | Yes | x | x |
| ADRB2 | Yes | x | x |
| ADRB3 | Yes | x | x |
| ADRM1 | Yes | x | x |
| AEBP1 | x | Yes | x |
| AFP | Yes | x | x |
| AGA | Yes | x | x |
| AGFG1 | Yes | x | x |
| AGRN | Yes | x | x |
| AGT | Yes | x | x |
| AGTPBP1 | x | Yes | x |
| AGTR1 | Yes | x | x |
| AGTR2 | Yes | x | x |
| AHRR | x | Yes | x |
| AHSA1 | Yes | x | x |
| AHSG | Yes | x | x |
| AIMP1 | Yes | x | x |
| AIRE | Yes | x | x |
| AJUBA | Yes | x | x |
| AKAP11 | Yes | x | x |
| AKAP5 | Yes | x | x |
| AKAP7 | Yes | x | x |
| AKR7A2 | x | Yes | x |
| AKT1 | Yes | x | x |
| AKT1S1 | Yes | x | x |
| ALB | Yes | x | x |
| ALDH18A1 | Yes | x | x |
| ALDH1A1 | Yes | x | x |
| ALDH1A2 | Yes | x | x |
| ALDH1A3 | Yes | x | x |
| ALDOA | Yes | x | x |
| ALDOC | Yes | x | x |
| ALMS1 | x | Yes | x |
| ALPG | Yes | x | x |
| ALPI | Yes | x | x |
| ALPL | Yes | x | x |
| AMBN | Yes | x | x |
| AMBRA1 | Yes | x | x |
| AMELX | Yes | x | x |
| AMER1 | Yes | x | x |
| AMOTL1 | Yes | x | x |
| AMOTL2 | Yes | x | x |
| AMPH | Yes | x | x |
| AMTN | Yes | x | x |
| ANAPC1 | Yes | x | x |
| ANAPC10 | Yes | x | x |
| ANAPC15 | Yes | x | x |
| ANAPC16 | Yes | x | x |
| ANAPC4 | Yes | x | x |
| ANAPC5 | Yes | x | x |
| ANAPC7 | Yes | x | x |
| ANG | x | Yes | x |
| ANK2 | Yes | x | x |
| ANK3 | Yes | x | x |
| ANKRD6 | Yes | x | x |
| ANKS4B | Yes | x | x |
| ANO1 | Yes | x | x |
| ANO2 | Yes | x | x |
| ANO6 | Yes | x | x |
| ANO8 | Yes | x | x |
| ANPEP | Yes | x | x |
| ANXA1 | Yes | x | x |
| ANXA2 | Yes | x | x |
| AOC1 | Yes | x | x |
| AOX1 | Yes | x | x |
| AP1B1 | Yes | x | x |
| AP1G1 | Yes | x | x |
| AP1M1 | Yes | x | x |
| AP1M2 | Yes | x | x |
| AP1S1 | Yes | x | x |
| AP1S2 | Yes | x | x |
| AP1S3 | Yes | x | x |
| AP2A1 | Yes | x | x |
| AP2A2 | Yes | x | x |
| AP2B1 | Yes | x | x |
| AP2M1 | Yes | x | x |
| AP2S1 | Yes | x | x |
| APBB2 | x | Yes | x |
| APC | Yes | x | x |
| APC2 | Yes | x | x |
| APEX1 | Yes | x | x |
| APIP | Yes | x | x |
| APLNR | Yes | x | x |
| APLP2 | Yes | x | x |
| APOA1 | Yes | x | x |
| APOA2 | Yes | x | x |
| APOA5 | Yes | x | x |
| APOB | Yes | x | x |
| APOE | Yes | x | x |
| APOL1 | Yes | x | x |
| APP | Yes | x | x |
| APRT | Yes | x | x |
| AQR | Yes | x | x |
| AR | Yes | x | x |
| ARAF | Yes | x | x |
| ARCN1 | Yes | x | x |
| AREG | Yes | x | x |
| ARF1 | Yes | x | x |
| ARF3 | Yes | x | x |
| ARF4 | Yes | x | x |
| ARF5 | Yes | x | x |
| ARF6 | Yes | x | x |
| ARFGAP1 | Yes | x | x |
| ARFGAP2 | Yes | x | x |
| ARFGAP3 | Yes | x | x |
| ARFIP1 | Yes | x | x |
| ARFIP2 | Yes | x | x |
| ARG1 | Yes | x | x |
| ARG2 | Yes | x | x |
| ARHGAP45 | Yes | x | x |
| ARHGDIA | Yes | x | x |
| ARHGEF19 | x | Yes | x |
| ARHGEF28 | x | Yes | x |
| ARID1B | x | Yes | x |
| ARMC6 | Yes | x | x |
| ARMC8 | Yes | x | x |
| ARNTL | Yes | x | x |
| ARNTL2 | Yes | x | x |
| ARPC1A | Yes | x | x |
| ARPC2 | Yes | x | x |
| ARPC4 | Yes | x | x |
| ARPC5 | Yes | x | x |
| ARRB1 | Yes | x | x |
| ARRB2 | Yes | x | x |
| ARSA | Yes | x | x |
| ARSB | Yes | x | x |
| ARSK | Yes | x | x |
| ART3 | Yes | x | x |
| ART4 | Yes | x | x |
| ASAH1 | Yes | x | x |
| ASAP1 | Yes | x | x |
| ASAP2 | Yes | x | x |
| ASB10 | Yes | x | x |
| ASB7 | Yes | x | x |
| ASL | Yes | x | x |
| ASPSCR1 | Yes | x | x |
| ASS1 | Yes | x | x |
| ATG10 | Yes | x | x |
| ATG101 | Yes | x | x |
| ATG12 | Yes | x | x |
| ATG13 | Yes | x | x |
| ATG14 | Yes | x | x |
| ATG16L1 | Yes | x | x |
| ATG16L2 | Yes | x | x |
| ATG2A | Yes | x | x |
| ATG2B | Yes | x | x |
| ATG3 | Yes | x | x |
| ATG4A | Yes | x | x |
| ATG4B | Yes | x | x |
| ATG4C | Yes | x | x |
| ATG4D | Yes | x | x |
| ATG5 | Yes | x | x |
| ATG7 | Yes | x | x |
| ATG9A | Yes | x | x |
| ATM | Yes | x | x |
| ATOX1 | Yes | x | x |
| ATP10A | x | Yes | x |
| ATP23 | Yes | x | x |
| ATP5F1A | Yes | x | x |
| ATP5F1B | Yes | x | x |
| ATP5MC1 | Yes | x | x |
| ATP6V0D2 | x | Yes | x |
| ATPAF2 | Yes | x | x |
| ATR | Yes | x | x |
| ATRIP | Yes | x | x |
| AURKA | Yes | x | x |
| AURKAIP1 | Yes | x | x |
| AURKB | Yes | x | x |
| AVP | Yes | x | x |
| AVPR2 | Yes | x | x |
| AXIN1 | Yes | x | x |
| AXIN2 | Yes | x | x |
| AXL | Yes | x | x |
| AZU1 | Yes | x | x |
| B3GALT6 | Yes | x | x |
| B3GAT1 | Yes | x | x |
| B3GAT3 | Yes | x | x |
| B4GALT7 | Yes | x | x |
| BACH2 | x | Yes | x |
| BAIAP2L1 | Yes | x | x |
| BAMBI | Yes | x | x |
| BANP | x | Yes | x |
| BARD1 | Yes | x | x |
| BAZ1B | Yes | x | x |
| BCAN | Yes | x | x |
| BCAP31 | Yes | x | x |
| BCAR1 | Yes | x | x |
| BCAR3 | x | Yes | x |
| BCL2L13 | Yes | x | x |
| BCS1L | Yes | x | x |
| BDKRB1 | Yes | x | x |
| BDKRB2 | Yes | x | x |
| BDNF | Yes | x | x |
| BECN1 | Yes | x | x |
| BEST1 | Yes | x | x |
| BEST2 | Yes | x | x |
| BEST3 | Yes | x | x |
| BEST4 | Yes | x | x |
| BET1 | Yes | x | x |
| BET1L | Yes | x | x |
| BGN | Yes | x | x |
| BHLHE40 | Yes | x | x |
| BHLHE41 | Yes | x | x |
| BIN1 | Yes | x | x |
| BIRC2 | Yes | x | x |
| BIRC3 | Yes | x | x |
| BLM | Yes | x | x |
| BMP15 | Yes | x | x |
| BMP2 | Yes | x | x |
| BMP2K | Yes | x | x |
| BMP4 | Yes | x | x |
| BNIP3 | Yes | x | x |
| BNIP3L | Yes | x | x |
| BOD1L1 | Yes | x | x |
| BPI | Yes | x | x |
| BPIFB2 | Yes | x | x |
| BPTF | Yes | x | x |
| BRAF | Yes | x | x |
| BRCA1 | Yes | x | x |
| BRCA2 | Yes | x | x |
| BRCC3 | Yes | x | x |
| BRIP1 | Yes | x | x |
| BSG | Yes | x | x |
| BST1 | Yes | x | x |
| BST2 | x | Yes | x |
| BTK | Yes | x | x |
| BTRC | Yes | x | x |
| BUB1B | Yes | x | x |
| BUB3 | Yes | x | x |
| BUD23 | Yes | x | x |
| C1QTNF8 | x | Yes | x |
| C2CD5 | Yes | x | x |
| C3 | Yes | x | x |
| C3AR1 | Yes | x | x |
| C4A | Yes | x | x |
| C4B | Yes | x | x |
| C5 | Yes | x | x |
| C5AR1 | Yes | x | x |
| C6orf120 | Yes | x | x |
| C9orf72 | Yes | x | x |
| CACNA1A | Yes | x | x |
| CACNA1B | Yes | x | x |
| CACNA1C | Yes | x | x |
| CACNA1D | Yes | x | x |
| CACNA1E | Yes | x | x |
| CACNA1F | Yes | x | x |
| CACNA1H | Yes | x | x |
| CACNA1S | Yes | x | x |
| CACNA2D1 | Yes | x | x |
| CACNA2D2 | Yes | x | x |
| CACNA2D3 | Yes | x | x |
| CACNA2D4 | Yes | x | x |
| CACNB1 | Yes | x | x |
| CACNB2 | Yes | x | x |
| CACNB3 | Yes | x | x |
| CACNB4 | Yes | x | x |
| CACNG1 | Yes | x | x |
| CACNG2 | Yes | x | x |
| CACNG4 | Yes | x | x |
| CACNG5 | Yes | x | x |
| CACNG7 | Yes | x | x |
| CACNG8 | Yes | x | x |
| CAD | Yes | x | x |
| CALCA | Yes | x | x |
| CALCB | Yes | x | x |
| CALCOCO2 | Yes | x | x |
| CALCR | Yes | x | x |
| CALCRL | Yes | x | x |
| CALM1 | Yes | x | x |
| CALM2 | Yes | x | x |
| CALM3 | Yes | x | x |
| CALML3 | Yes | x | x |
| CALML4 | Yes | x | x |
| CALML5 | Yes | x | x |
| CALML6 | Yes | x | x |
| CALR | Yes | x | x |
| CALU | Yes | x | x |
| CAMK1D | x | Yes | x |
| CAMK1G | Yes | x | x |
| CAMK2A | Yes | x | x |
| CAMK2B | Yes | x | x |
| CAMK2D | Yes | x | x |
| CAMK2G | Yes | x | x |
| CAMP | Yes | x | x |
| CANT1 | Yes | x | x |
| CANX | Yes | x | x |
| CAP1 | Yes | x | x |
| CAPZA1 | Yes | x | x |
| CAPZA2 | Yes | x | x |
| CAPZA3 | Yes | x | x |
| CAPZB | Yes | x | x |
| CARD11 | Yes | x | x |
| CASP8 | Yes | x | x |
| CASR | Yes | x | x |
| CATIP | Yes | x | x |
| CATSPER1 | Yes | x | x |
| CATSPER2 | Yes | x | x |
| CATSPER3 | Yes | x | x |
| CATSPER4 | Yes | x | x |
| CATSPERB | Yes | x | x |
| CATSPERD | Yes | x | x |
| CATSPERE | Yes | x | x |
| CATSPERG | Yes | x | x |
| CBL | Yes | x | x |
| CBS | Yes | x | x |
| CCDC154 | x | Yes | x |
| CCDC17 | Yes | x | x |
| CCDC88C | Yes | x | x |
| CCL1 | Yes | x | x |
| CCL11 | Yes | x | x |
| CCL13 | Yes | x | x |
| CCL16 | Yes | x | x |
| CCL19 | Yes | x | x |
| CCL2 | Yes | x | x |
| CCL20 | Yes | x | x |
| CCL21 | Yes | x | x |
| CCL25 | Yes | x | x |
| CCL27 | Yes | x | x |
| CCL28 | Yes | x | x |
| CCL4 | Yes | x | x |
| CCL4L1 | Yes | x | x |
| CCL4L2 | Yes | x | x |
| CCL5 | Yes | x | x |
| CCL7 | Yes | x | x |
| CCN1 | Yes | x | x |
| CCN4 | Yes | x | x |
| CCNA1 | Yes | x | x |
| CCNA2 | Yes | x | x |
| CCND1 | Yes | x | x |
| CCNE1 | Yes | x | x |
| CCNE2 | Yes | x | x |
| CCNH | Yes | x | x |
| CCR1 | Yes | x | x |
| CCR10 | Yes | x | x |
| CCR2 | Yes | x | x |
| CCR3 | Yes | x | x |
| CCR4 | Yes | x | x |
| CCR5 | Yes | x | x |
| CCR6 | Yes | x | x |
| CCR7 | Yes | x | x |
| CCR8 | Yes | x | x |
| CCR9 | Yes | x | x |
| CCT2 | Yes | x | x |
| CCT3 | Yes | x | x |
| CCT4 | Yes | x | x |
| CCT5 | Yes | x | x |
| CCT6A | Yes | x | x |
| CCT7 | Yes | x | x |
| CCT8 | Yes | x | x |
| CCZ1 | x | Yes | x |
| CD101 | Yes | x | x |
| CD109 | Yes | x | x |
| CD151 | Yes | x | x |
| CD1D | Yes | x | x |
| CD2 | Yes | x | x |
| CD247 | Yes | x | x |
| CD274 | Yes | x | x |
| CD28 | Yes | x | x |
| CD300C | Yes | x | x |
| CD3D | Yes | x | x |
| CD3E | Yes | x | x |
| CD3G | Yes | x | x |
| CD4 | Yes | x | x |
| CD40LG | Yes | x | x |
| CD47 | Yes | x | x |
| CD5 | Yes | x | x |
| CD52 | Yes | x | x |
| CD55 | Yes | x | x |
| CD59 | Yes | x | x |
| CD6 | Yes | x | x |
| CD74 | Yes | x | x |
| CD80 | Yes | x | x |
| CD86 | Yes | x | x |
| CD8A | Yes | x | x |
| CD8B | Yes | x | x |
| CDA | Yes | x | x |
| CDC16 | Yes | x | x |
| CDC20 | Yes | x | x |
| CDC23 | Yes | x | x |
| CDC25A | Yes | x | x |
| CDC26 | Yes | x | x |
| CDC27 | Yes | x | x |
| CDC37 | Yes | x | x |
| CDC42 | Yes | x | x |
| CDC5L | Yes | x | x |
| CDC6 | Yes | x | x |
| CDH1 | Yes | x | x |
| CDH10 | Yes | x | x |
| CDH11 | Yes | x | x |
| CDH12 | Yes | x | x |
| CDH13 | Yes | x | x |
| CDH15 | Yes | x | x |
| CDH17 | Yes | x | x |
| CDH18 | Yes | x | x |
| CDH2 | Yes | x | x |
| CDH23 | Yes | x | x |
| CDH24 | Yes | x | x |
| CDH3 | Yes | x | x |
| CDH4 | Yes | x | x |
| CDH5 | Yes | x | x |
| CDH6 | Yes | x | x |
| CDH7 | Yes | x | x |
| CDH8 | Yes | x | x |
| CDH9 | Yes | x | x |
| CDHR2 | Yes | x | x |
| CDHR5 | Yes | x | x |
| CDIPT | Yes | x | x |
| CDK1 | Yes | x | x |
| CDK11A | x | Yes | x |
| CDK18 | x | Yes | x |
| CDK2 | Yes | x | x |
| CDK2AP1 | x | Yes | x |
| CDK7 | Yes | x | x |
| CDKN1A | Yes | x | x |
| CDKN1B | Yes | x | x |
| CEACAM5 | Yes | x | x |
| CEACAM7 | Yes | x | x |
| CELA1 | Yes | x | x |
| CELA2A | Yes | x | x |
| CELA2B | Yes | x | x |
| CELA3A | Yes | x | x |
| CELA3B | Yes | x | x |
| CELSR1 | Yes | x | x |
| CENPE | Yes | x | x |
| CENPS | Yes | x | x |
| CENPS-CORT | Yes | x | x |
| CENPX | Yes | x | x |
| CEP290 | Yes | x | x |
| CETN2 | Yes | x | x |
| CFP | Yes | x | x |
| CFTR | Yes | x | x |
| CGA | Yes | x | x |
| CHCHD1 | Yes | x | x |
| CHD1L | Yes | x | x |
| CHGB | Yes | x | x |
| CHI3L1 | Yes | x | x |
| CHIA | x | Yes | x |
| CHIT1 | Yes | x | x |
| CHM | Yes | x | x |
| CHML | Yes | x | x |
| CHMP2A | Yes | x | x |
| CHMP2B | Yes | x | x |
| CHMP4A | Yes | x | x |
| CHMP4B | Yes | x | x |
| CHMP4C | Yes | x | x |
| CHMP6 | Yes | x | x |
| CHMP7 | Yes | x | x |
| CHORDC1 | Yes | x | x |
| CHPF | Yes | x | x |
| CHPF2 | Yes | x | x |
| CHRDL1 | Yes | x | x |
| CHRM2 | Yes | x | x |
| CHRM4 | Yes | x | x |
| CHRNA3 | Yes | x | x |
| CHRNA5 | Yes | x | x |
| CHRNB4 | Yes | x | x |
| CHST11 | Yes | x | x |
| CHST12 | Yes | x | x |
| CHST13 | Yes | x | x |
| CHST14 | Yes | x | x |
| CHST15 | Yes | x | x |
| CHST3 | Yes | x | x |
| CHST7 | Yes | x | x |
| CHSY1 | Yes | x | x |
| CHSY3 | Yes | x | x |
| CIB2 | Yes | x | x |
| CIITA | x | Yes | x |
| CITED1 | Yes | x | x |
| CITED2 | Yes | x | x |
| CITED4 | Yes | x | x |
| CKAP4 | Yes | x | x |
| CKS1B | Yes | x | x |
| CLCA1 | Yes | x | x |
| CLCN1 | Yes | x | x |
| CLCN2 | Yes | x | x |
| CLCN3 | Yes | x | x |
| CLEC1B | Yes | x | x |
| CLINT1 | Yes | x | x |
| CLOCK | Yes | x | x |
| CLRN1 | Yes | x | x |
| CLSPN | Yes | x | x |
| CLTA | Yes | x | x |
| CLTB | Yes | x | x |
| CLTC | Yes | x | x |
| CLTCL1 | Yes | x | x |
| CNIH1 | Yes | x | x |
| CNIH2 | Yes | x | x |
| CNIH3 | Yes | x | x |
| CNKSR2 | Yes | x | x |
| CNN2 | Yes | x | x |
| CNR1 | Yes | x | x |
| CNR2 | Yes | x | x |
| CNTN2 | Yes | x | x |
| CNTN3 | Yes | x | x |
| CNTN4 | Yes | x | x |
| CNTN5 | Yes | x | x |
| CNTNAP2 | x | Yes | x |
| COA3 | Yes | x | x |
| COA5 | Yes | x | x |
| COA6 | Yes | x | x |
| COL13A1 | x | Yes | x |
| COL14A1 | Yes | x | x |
| COL18A1 | Yes | x | x |
| COL1A1 | Yes | x | x |
| COL1A2 | Yes | x | x |
| COL3A1 | Yes | x | x |
| COL4A1 | Yes | x | x |
| COL4A2 | Yes | x | x |
| COL4A3 | Yes | x | x |
| COL4A4 | Yes | x | x |
| COL4A5 | Yes | x | x |
| COL4A6 | Yes | x | x |
| COL6A1 | Yes | x | x |
| COL6A2 | Yes | x | x |
| COL6A3 | Yes | x | x |
| COL7A1 | Yes | x | x |
| COMP | Yes | x | x |
| COP1 | Yes | x | x |
| COPA | Yes | x | x |
| COPB1 | Yes | x | x |
| COPB2 | Yes | x | x |
| COPE | Yes | x | x |
| COPG1 | Yes | x | x |
| COPG2 | Yes | x | x |
| COPS8 | x | Yes | x |
| COPZ1 | Yes | x | x |
| COPZ2 | Yes | x | x |
| COQ2 | Yes | x | x |
| CORT | Yes | x | x |
| COTL1 | Yes | x | x |
| COX1 | Yes | x | x |
| COX10 | Yes | x | x |
| COX11 | Yes | x | x |
| COX14 | Yes | x | x |
| COX15 | Yes | x | x |
| COX17 | Yes | x | x |
| COX19 | Yes | x | x |
| COX2 | Yes | x | x |
| COX20 | Yes | x | x |
| COX3 | Yes | x | x |
| COX4I1 | Yes | x | x |
| COX4I2 | Yes | x | x |
| COX5A | Yes | x | x |
| COX5B | Yes | x | x |
| COX6A1 | Yes | x | x |
| COX6A2 | Yes | x | x |
| COX6B1 | Yes | x | x |
| CP | Yes | x | x |
| CPA1 | Yes | x | x |
| CPA2 | Yes | x | x |
| CPA3 | Yes | x | x |
| CPB1 | Yes | x | x |
| CPLX1 | Yes | x | x |
| CPM | Yes | x | x |
| CPNE4 | x | Yes | x |
| CPPED1 | Yes | x | x |
| CPS1 | Yes | x | x |
| CPT1B | Yes | x | x |
| CRABP1 | Yes | x | x |
| CRACR2A | Yes | x | x |
| CREB1 | Yes | x | x |
| CREBBP | Yes | x | x |
| CREG1 | Yes | x | x |
| CRH | Yes | x | x |
| CRHR1 | Yes | x | x |
| CRHR2 | Yes | x | x |
| CRISP3 | Yes | x | x |
| CRK | Yes | x | x |
| CRKL | Yes | x | x |
| CRP | x | Yes | x |
| CRY1 | Yes | x | x |
| CRY2 | Yes | x | x |
| CS | Yes | x | x |
| CSF1 | Yes | x | x |
| CSGALNACT2 | Yes | x | x |
| CSK | Yes | x | x |
| CSKMT | Yes | x | x |
| CSNK1A1 | Yes | x | x |
| CSNK1D | Yes | x | x |
| CSNK1E | Yes | x | x |
| CSNK1G1 | Yes | x | x |
| CSNK1G2 | Yes | x | x |
| CSNK2A1 | Yes | x | x |
| CSNK2A2 | Yes | x | x |
| CSNK2A3 | Yes | x | x |
| CSNK2B | Yes | x | x |
| CSPG4 | Yes | x | x |
| CSPG5 | Yes | x | x |
| CST3 | Yes | x | x |
| CSTB | Yes | x | x |
| CSTF1 | Yes | x | x |
| CTHRC1 | Yes | x | x |
| CTLA4 | Yes | x | x |
| CTNNA1 | Yes | x | x |
| CTNNB1 | Yes | x | x |
| CTNND1 | Yes | x | x |
| CTNND2 | Yes | x | x |
| CTRB1 | Yes | x | x |
| CTRB2 | Yes | x | x |
| CTRC | Yes | x | x |
| CTRL | Yes | x | x |
| CTSA | Yes | x | x |
| CTSB | Yes | x | x |
| CTSC | Yes | x | x |
| CTSD | Yes | x | x |
| CTSE | Yes | x | x |
| CTSF | Yes | x | x |
| CTSG | Yes | x | x |
| CTSK | Yes | x | x |
| CTSL | Yes | x | x |
| CTSO | Yes | x | x |
| CTSS | Yes | x | x |
| CTSV | Yes | x | x |
| CTSZ | Yes | x | x |
| CUL1 | Yes | x | x |
| CUL2 | Yes | x | x |
| CUL3 | Yes | x | x |
| CUL4A | Yes | x | x |
| CUL4B | Yes | x | x |
| CX3CL1 | Yes | x | x |
| CX3CR1 | Yes | x | x |
| CXCL1 | Yes | x | x |
| CXCL11 | Yes | x | x |
| CXCL12 | Yes | x | x |
| CXCL13 | Yes | x | x |
| CXCL16 | Yes | x | x |
| CXCL2 | Yes | x | x |
| CXCL3 | Yes | x | x |
| CXCL5 | Yes | x | x |
| CXCL6 | Yes | x | x |
| CXCL8 | Yes | x | x |
| CXCL9 | Yes | x | x |
| CXCR1 | Yes | x | x |
| CXCR2 | Yes | x | x |
| CXCR3 | Yes | x | x |
| CXCR4 | Yes | x | x |
| CXCR5 | Yes | x | x |
| CXCR6 | Yes | x | x |
| CXXC1 | Yes | x | x |
| CYB5A | Yes | x | x |
| CYB5R3 | Yes | x | x |
| CYC1 | Yes | x | x |
| CYCS | Yes | x | x |
| CYFIP1 | Yes | x | x |
| CYP1A1 | Yes | x | x |
| CYP1A2 | Yes | x | x |
| CYP1B1 | Yes | x | x |
| CYP26A1 | Yes | x | x |
| CYP26B1 | Yes | x | x |
| CYP26C1 | Yes | x | x |
| CYP2A6 | Yes | x | x |
| CYP2B6 | Yes | x | x |
| CYP2C18 | Yes | x | x |
| CYP2C8 | Yes | x | x |
| CYP2C9 | Yes | x | x |
| CYP2S1 | Yes | x | x |
| CYP3A4 | Yes | x | x |
| CYP3A5 | Yes | x | x |
| CYP3A7 | Yes | x | x |
| CYP3A7-CYP3A51P | Yes | x | x |
| CYP4A11 | Yes | x | x |
| CYP51A1 | Yes | x | x |
| CYREN | Yes | x | x |
| CYTB | Yes | x | x |
| CYTH1 | Yes | x | x |
| CYTH3 | Yes | x | x |
| DAAM1 | Yes | x | x |
| DAAM2 | Yes | x | x |
| DAB1 | Yes | x | x |
| DAB2 | Yes | x | x |
| DACT1 | Yes | x | x |
| DACT2 | Yes | x | x |
| DBNL | Yes | x | x |
| DCHS1 | Yes | x | x |
| DCHS2 | Yes | x | x |
| DCLRE1A | Yes | x | x |
| DCLRE1B | Yes | x | x |
| DCLRE1C | Yes | x | x |
| DCN | Yes | x | x |
| DCTN1 | Yes | x | x |
| DCTN2 | Yes | x | x |
| DCTN3 | Yes | x | x |
| DCTN4 | Yes | x | x |
| DCTN5 | Yes | x | x |
| DCTN6 | Yes | x | x |
| DDB1 | Yes | x | x |
| DDB2 | Yes | x | x |
| DDC | Yes | x | x |
| DDX11 | x | Yes | x |
| DDX58 | x | Yes | x |
| DDX60 | x | Yes | x |
| DEFA3 | Yes | x | x |
| DEFA4 | Yes | x | x |
| DERL1 | Yes | x | x |
| DERL2 | Yes | x | x |
| DHCR24 | Yes | x | x |
| DHCR7 | Yes | x | x |
| DHH | Yes | x | x |
| DHX37 | x | Yes | x |
| DHX58 | x | Yes | x |
| DLAT | Yes | x | x |
| DLD | Yes | x | x |
| DLG1 | Yes | x | x |
| DLG4 | Yes | x | x |
| DMKN | x | Yes | x |
| DMP1 | Yes | x | x |
| DNA2 | Yes | x | x |
| DNAJA1 | Yes | x | x |
| DNAJC11 | Yes | x | x |
| DNAJC19 | Yes | x | x |
| DNAJC3 | Yes | x | x |
| DNAJC6 | Yes | x | x |
| DNAJC8 | x | Yes | x |
| DNASE1L1 | Yes | x | x |
| DNASE2 | Yes | x | x |
| DNM1 | Yes | x | x |
| DNM2 | Yes | x | x |
| DOCK2 | Yes | x | x |
| DPM1 | Yes | x | x |
| DPM2 | Yes | x | x |
| DPP7 | Yes | x | x |
| DPT | Yes | x | x |
| DRD1 | Yes | x | x |
| DRD2 | Yes | x | x |
| DRD3 | Yes | x | x |
| DRD4 | Yes | x | x |
| DRD5 | Yes | x | x |
| DSE | Yes | x | x |
| DSEL | Yes | x | x |
| DSN1 | Yes | x | x |
| DSP | Yes | x | x |
| DTNA | x | Yes | x |
| DVL1 | Yes | x | x |
| DVL2 | Yes | x | x |
| DVL3 | Yes | x | x |
| DYDC1 | x | Yes | x |
| DYNC1H1 | Yes | x | x |
| DYNC1I1 | Yes | x | x |
| DYNC1I2 | Yes | x | x |
| DYNC1LI1 | Yes | x | x |
| DYNC1LI2 | Yes | x | x |
| DYNLL1 | Yes | x | x |
| DYNLL2 | Yes | x | x |
| DYRK4 | x | Yes | x |
| DYSF | x | Yes | x |
| DZIP1 | x | Yes | x |
| ECSIT | Yes | x | x |
| EDN1 | Yes | x | x |
| EEF1A1 | Yes | x | x |
| EEF1AKMT2 | Yes | x | x |
| EEF1B2 | Yes | x | x |
| EFNA1 | Yes | x | x |
| EFNA2 | Yes | x | x |
| EFNA3 | Yes | x | x |
| EFNA4 | Yes | x | x |
| EFNA5 | Yes | x | x |
| EFNB1 | Yes | x | x |
| EFNB2 | Yes | x | x |
| EFNB3 | Yes | x | x |
| EFTUD2 | Yes | x | x |
| EGF | Yes | x | x |
| EGFR | Yes | x | x |
| EGLN1 | Yes | x | x |
| EGR1 | x | Yes | x |
| EIF1AX | Yes | x | x |
| EIF2AK2 | x | Yes | x |
| EIF2S1 | Yes | x | x |
| EIF2S3 | Yes | x | x |
| EIF3E | Yes | x | x |
| EIF3G | Yes | x | x |
| EIF3I | Yes | x | x |
| EIF3M | Yes | x | x |
| EIF5B | Yes | x | x |
| ELANE | Yes | x | x |
| ELL | x | Yes | x |
| ELN | Yes | x | x |
| ELOB | Yes | x | x |
| ELOC | Yes | x | x |
| ELP6 | Yes | x | x |
| EME1 | Yes | x | x |
| EME2 | Yes | x | x |
| ENAH | Yes | x | x |
| ENAM | Yes | x | x |
| ENOPH1 | Yes | x | x |
| EP300 | Yes | x | x |
| EPAS1 | Yes | x | x |
| EPB41L1 | Yes | x | x |
| EPDR1 | Yes | x | x |
| EPN1 | Yes | x | x |
| EPN2 | Yes | x | x |
| EPPK1 | x | Yes | x |
| EPRS1 | Yes | x | x |
| EPS15 | Yes | x | x |
| EPS15L1 | Yes | x | x |
| EPS8 | Yes | x | x |
| ERAL1 | Yes | x | x |
| ERBB2 | Yes | x | x |
| ERBB3 | Yes | x | x |
| ERBB4 | Yes | x | x |
| ERCC1 | Yes | x | x |
| ERCC2 | Yes | x | x |
| ERCC3 | Yes | x | x |
| ERCC4 | Yes | x | x |
| ERCC5 | Yes | x | x |
| ERCC6 | Yes | x | x |
| ERCC8 | Yes | x | x |
| ERG28 | Yes | x | x |
| ERLEC1 | Yes | x | x |
| ERLIN1 | Yes | x | x |
| ERLIN2 | Yes | x | x |
| ERP44 | Yes | x | x |
| ERVW-1 | Yes | x | x |
| ESCO1 | Yes | x | x |
| ESF1 | Yes | x | x |
| ESR1 | Yes | x | x |
| ESR2 | Yes | x | x |
| ESRRB | x | Yes | x |
| ETF1 | Yes | x | x |
| ETFA | Yes | x | x |
| ETFBKMT | Yes | x | x |
| EVA1A | Yes | x | x |
| EVL | Yes | x | x |
| EXO1 | Yes | x | x |
| EXOC1 | Yes | x | x |
| EXOC2 | Yes | x | x |
| EXOC3 | Yes | x | x |
| EXOC4 | Yes | x | x |
| EXOC5 | Yes | x | x |
| EXOC6 | Yes | x | x |
| EXOC6B | Yes | x | x |
| EXOC7 | Yes | x | x |
| EXOC8 | Yes | x | x |
| EXT1 | Yes | x | x |
| EXTL2 | Yes | x | x |
| EXTL3 | Yes | x | x |
| EZR | Yes | x | x |
| F11R | Yes | x | x |
| F2 | Yes | x | x |
| F5 | Yes | x | x |
| F8 | Yes | x | x |
| F9 | Yes | x | x |
| FAAP100 | Yes | x | x |
| FAAP24 | Yes | x | x |
| FABP5 | Yes | x | x |
| FAF2 | Yes | x | x |
| FAM107B | x | Yes | x |
| FAM120B | x | Yes | x |
| FAM136A | Yes | x | x |
| FAM13B | Yes | x | x |
| FAM171A2 | Yes | x | x |
| FAM20A | Yes | x | x |
| FAM20B | Yes | x | x |
| FAM20C | Yes | x | x |
| FAN1 | Yes | x | x |
| FANCA | Yes | x | x |
| FANCB | Yes | x | x |
| FANCC | Yes | x | x |
| FANCD2 | Yes | x | x |
| FANCE | Yes | x | x |
| FANCF | Yes | x | x |
| FANCG | Yes | x | x |
| FANCI | Yes | x | x |
| FANCL | Yes | x | x |
| FANCM | Yes | x | x |
| FAT4 | Yes | x | x |
| FAU | Yes | x | x |
| FBN1 | Yes | x | x |
| FBXL17 | Yes | x | x |
| FBXL3 | Yes | x | x |
| FBXL7 | Yes | x | x |
| FBXO3 | x | Yes | x |
| FBXO5 | Yes | x | x |
| FBXW7 | Yes | x | x |
| FCER1A | Yes | x | x |
| FCER1G | Yes | x | x |
| FCGR1A | x | Yes | x |
| FCGR1BP | x | Yes | x |
| FCGR3A | Yes | x | x |
| FCGR3B | Yes | x | x |
| FCHO1 | Yes | x | x |
| FCHO2 | Yes | x | x |
| FDFT1 | Yes | x | x |
| FDPS | Yes | x | x |
| FDXR | Yes | x | x |
| FEN1 | Yes | x | x |
| FGA | Yes | x | x |
| FGF17 | x | Yes | x |
| FGF2 | Yes | x | x |
| FGF23 | Yes | x | x |
| FGG | Yes | x | x |
| FIGLA | Yes | x | x |
| FKBP1A | Yes | x | x |
| FKBP4 | Yes | x | x |
| FKBP5 | Yes | x | x |
| FLG2 | Yes | x | x |
| FLNA | Yes | x | x |
| FLNC | Yes | x | x |
| FMR1 | Yes | x | x |
| FNBP1 | Yes | x | x |
| FNBP1L | Yes | x | x |
| FOLR1 | Yes | x | x |
| FOLR2 | Yes | x | x |
| FOLR3 | Yes | x | x |
| FOXA1 | Yes | x | x |
| FOXO1 | Yes | x | x |
| FOXRED1 | Yes | x | x |
| FPR1 | Yes | x | x |
| FPR2 | Yes | x | x |
| FPR3 | Yes | x | x |
| FRAT1 | Yes | x | x |
| FRAT2 | Yes | x | x |
| FRK | Yes | x | x |
| FSHB | Yes | x | x |
| FSHR | Yes | x | x |
| FSTL1 | Yes | x | x |
| FSTL3 | Yes | x | x |
| FTH1 | Yes | x | x |
| FTL | Yes | x | x |
| FTSJ1 | Yes | x | x |
| FUCA1 | Yes | x | x |
| FUCA2 | Yes | x | x |
| FUNDC1 | Yes | x | x |
| FXN | Yes | x | x |
| FYB1 | Yes | x | x |
| FYN | Yes | x | x |
| FZD1 | Yes | x | x |
| FZD10 | Yes | x | x |
| FZD2 | Yes | x | x |
| FZD3 | Yes | x | x |
| FZD4 | Yes | x | x |
| FZD5 | Yes | x | x |
| FZD6 | Yes | x | x |
| FZD7 | Yes | x | x |
| FZD8 | Yes | x | x |
| FZD9 | Yes | x | x |
| FZR1 | Yes | x | x |
| GABARAP | Yes | x | x |
| GABARAPL1 | Yes | x | x |
| GABARAPL2 | Yes | x | x |
| GABBR1 | Yes | x | x |
| GABRB1 | x | Yes | x |
| GABRG2 | Yes | x | x |
| GAD1 | Yes | x | x |
| GADD45GIP1 | Yes | x | x |
| GAK | Yes | x | x |
| GAL | Yes | x | x |
| GALNS | Yes | x | x |
| GALNT10 | x | Yes | x |
| GALR1 | Yes | x | x |
| GALR2 | Yes | x | x |
| GALR3 | Yes | x | x |
| GAPVD1 | Yes | x | x |
| GAS6 | Yes | x | x |
| GATA3 | Yes | x | x |
| GBE1 | Yes | x | x |
| GBF1 | Yes | x | x |
| GBP1 | x | Yes | x |
| GBP2 | x | Yes | x |
| GBP3 | x | Yes | x |
| GBP4 | x | Yes | x |
| GBP5 | x | Yes | x |
| GBP6 | x | Yes | x |
| GBP7 | x | Yes | x |
| GCA | Yes | x | x |
| GCC2 | Yes | x | x |
| GCGR | Yes | x | x |
| GCK | x | Yes | x |
| GCNT1 | Yes | x | x |
| GCNT2 | Yes | x | x |
| GCNT4 | Yes | x | x |
| GDI1 | Yes | x | x |
| GDI2 | Yes | x | x |
| GEN1 | Yes | x | x |
| GET3 | Yes | x | x |
| GFM1 | Yes | x | x |
| GFM2 | Yes | x | x |
| GGA1 | Yes | x | x |
| GGA2 | Yes | x | x |
| GGH | Yes | x | x |
| GHDC | Yes | x | x |
| GHRH | Yes | x | x |
| GHRHR | Yes | x | x |
| GID4 | Yes | x | x |
| GID8 | Yes | x | x |
| GIP | Yes | x | x |
| GIPR | Yes | x | x |
| GJA1 | Yes | x | x |
| GLA | Yes | x | x |
| GLB1 | Yes | x | x |
| GLCE | Yes | x | x |
| GLI1 | Yes | x | x |
| GLI2 | Yes | x | x |
| GLI3 | Yes | x | x |
| GLMN | Yes | x | x |
| GLP1R | Yes | x | x |
| GLP2R | Yes | x | x |
| GLRA1 | Yes | x | x |
| GLRA3 | Yes | x | x |
| GLRA4 | Yes | x | x |
| GM2A | Yes | x | x |
| GMNN | Yes | x | x |
| GNAI1 | Yes | x | x |
| GNAI2 | Yes | x | x |
| GNAI3 | Yes | x | x |
| GNAO1 | Yes | x | x |
| GNAQ | Yes | x | x |
| GNAS | Yes | x | x |
| GNAT3 | Yes | x | x |
| GNAZ | Yes | x | x |
| GNB1 | Yes | x | x |
| GNB2 | Yes | x | x |
| GNB3 | Yes | x | x |
| GNB4 | Yes | x | x |
| GNB5 | Yes | x | x |
| GNG10 | Yes | x | x |
| GNG11 | Yes | x | x |
| GNG12 | Yes | x | x |
| GNG13 | Yes | x | x |
| GNG2 | Yes | x | x |
| GNG3 | Yes | x | x |
| GNG4 | Yes | x | x |
| GNG5 | Yes | x | x |
| GNG7 | Yes | x | x |
| GNG8 | Yes | x | x |
| GNGT1 | Yes | x | x |
| GNGT2 | Yes | x | x |
| GNS | Yes | x | x |
| GOLGA2 | Yes | x | x |
| GOLGA7 | Yes | x | x |
| GOLM1 | Yes | x | x |
| GOPC | Yes | x | x |
| GORASP1 | Yes | x | x |
| GOSR1 | Yes | x | x |
| GOSR2 | Yes | x | x |
| GP2 | Yes | x | x |
| GP6 | Yes | x | x |
| GPBAR1 | Yes | x | x |
| GPBP1L1 | Yes | x | x |
| GPC1 | Yes | x | x |
| GPC2 | Yes | x | x |
| GPC3 | Yes | x | x |
| GPC4 | Yes | x | x |
| GPC5 | Yes | x | x |
| GPC6 | Yes | x | x |
| GPER1 | Yes | x | x |
| GPHA2 | Yes | x | x |
| GPHB5 | Yes | x | x |
| GPI | Yes | x | x |
| GPLD1 | Yes | x | x |
| GPN1 | Yes | x | x |
| GPR15 | Yes | x | x |
| GPR150 | Yes | x | x |
| GPR17 | Yes | x | x |
| GPR176 | Yes | x | x |
| GPR18 | Yes | x | x |
| GPR183 | Yes | x | x |
| GPR20 | Yes | x | x |
| GPR25 | Yes | x | x |
| GPR27 | Yes | x | x |
| GPR31 | Yes | x | x |
| GPR32 | Yes | x | x |
| GPR37 | Yes | x | x |
| GPR37L1 | Yes | x | x |
| GPR39 | Yes | x | x |
| GPR45 | Yes | x | x |
| GPR55 | Yes | x | x |
| GPR63 | x | Yes | x |
| GPR83 | Yes | x | x |
| GPR84 | Yes | x | x |
| GPRC5A | Yes | x | x |
| GPS2 | Yes | x | x |
| GPSM1 | Yes | x | x |
| GPSM2 | Yes | x | x |
| GPSM3 | Yes | x | x |
| GRAMD1B | x | Yes | x |
| GRAMD4 | x | Yes | x |
| GRAP | Yes | x | x |
| GRAP2 | Yes | x | x |
| GREB1 | x | Yes | x |
| GRIA1 | Yes | x | x |
| GRIA2 | Yes | x | x |
| GRIA3 | Yes | x | x |
| GRIA4 | Yes | x | x |
| GRIN1 | Yes | x | x |
| GRIN2A | Yes | x | x |
| GRIN2B | Yes | x | x |
| GRIN2C | Yes | x | x |
| GRIN2D | Yes | x | x |
| GRIN3A | Yes | x | x |
| GRIN3B | Yes | x | x |
| GRM2 | Yes | x | x |
| GRM3 | Yes | x | x |
| GRM4 | Yes | x | x |
| GRM6 | Yes | x | x |
| GRM7 | Yes | x | x |
| GRM8 | Yes | x | x |
| GRN | Yes | x | x |
| GRPEL1 | Yes | x | x |
| GRPEL2 | Yes | x | x |
| GSDMD | Yes | x | x |
| GSK3A | Yes | x | x |
| GSK3B | Yes | x | x |
| GTF2H1 | Yes | x | x |
| GTF2H2 | Yes | x | x |
| GTF2H3 | Yes | x | x |
| GTF2H4 | Yes | x | x |
| GTF2H5 | Yes | x | x |
| GTF2IRD1 | Yes | x | x |
| GTSE1 | Yes | x | x |
| GUF1 | Yes | x | x |
| GUSB | Yes | x | x |
| H2AX | Yes | x | x |
| H2BC1 | Yes | x | x |
| H2BC11 | Yes | x | x |
| H2BC12 | Yes | x | x |
| H2BC13 | Yes | x | x |
| H2BC14 | Yes | x | x |
| H2BC15 | Yes | x | x |
| H2BC17 | Yes | x | x |
| H2BC21 | Yes | x | x |
| H2BC3 | Yes | x | x |
| H2BC5 | Yes | x | x |
| H2BC9 | Yes | x | x |
| H3-4 | Yes | x | x |
| HBB | Yes | x | x |
| HCAR1 | Yes | x | x |
| HCAR2 | Yes | x | x |
| HCAR3 | Yes | x | x |
| HCFC1 | Yes | x | x |
| HCK | Yes | x | x |
| HCLS1 | Yes | x | x |
| HEBP1 | Yes | x | x |
| HEBP2 | Yes | x | x |
| HERC2 | Yes | x | x |
| HERC5 | x | Yes | x |
| HERC6 | x | Yes | x |
| HES1 | Yes | x | x |
| HEXB | Yes | x | x |
| HGF | Yes | x | x |
| HGS | Yes | x | x |
| HIBCH | Yes | x | x |
| HIF1A | Yes | x | x |
| HIF3A | Yes | x | x |
| HIP1 | Yes | x | x |
| HIP1R | Yes | x | x |
| HIVEP2 | x | Yes | x |
| HIVEP3 | Yes | x | x |
| HLA-C | x | Yes | x |
| HLA-DMB | Yes | x | x |
| HLA-DOA | Yes | x | x |
| HLA-DOB | Yes | x | x |
| HLA-DRA | Yes | x | x |
| HMGCR | Yes | x | x |
| HMGCS1 | Yes | x | x |
| HMGCS2 | Yes | x | x |
| HMGN1 | Yes | x | x |
| HOMEZ | Yes | x | x |
| HOXA11 | Yes | x | x |
| HOXA5 | Yes | x | x |
| HOXA6 | Yes | x | x |
| HOXA7 | Yes | x | x |
| HOXB4 | Yes | x | x |
| HOXB5 | Yes | x | x |
| HOXB6 | Yes | x | x |
| HOXB7 | Yes | x | x |
| HOXC4 | Yes | x | x |
| HOXC5 | Yes | x | x |
| HOXC6 | Yes | x | x |
| HOXC8 | Yes | x | x |
| HOXD4 | Yes | x | x |
| HP | Yes | x | x |
| HPSE | Yes | x | x |
| HRAS | Yes | x | x |
| HRC | Yes | x | x |
| HRH1 | x | Yes | x |
| HRH2 | Yes | x | x |
| HRH3 | Yes | x | x |
| HRH4 | Yes | x | x |
| HRNR | Yes | x | x |
| HS2ST1 | Yes | x | x |
| HS3ST1 | Yes | x | x |
| HS6ST1 | Yes | x | x |
| HSCB | Yes | x | x |
| HSD17B7 | Yes | x | x |
| HSF1 | Yes | x | x |
| HSP90AA1 | Yes | x | x |
| HSP90AB1 | Yes | x | x |
| HSP90B1 | Yes | x | x |
| HSPA12A | x | Yes | x |
| HSPA1A | Yes | x | x |
| HSPA1B | Yes | x | x |
| HSPA1L | Yes | x | x |
| HSPA2 | Yes | x | x |
| HSPA4 | Yes | x | x |
| HSPA8 | Yes | x | x |
| HSPA9 | Yes | x | x |
| HSPB7 | x | Yes | x |
| HSPD1 | Yes | x | x |
| HSPG2 | Yes | x | x |
| HTATSF1 | Yes | x | x |
| HTR1A | Yes | x | x |
| HTR1B | Yes | x | x |
| HTR1D | Yes | x | x |
| HTR1E | Yes | x | x |
| HTR1F | Yes | x | x |
| HTR3A | Yes | x | x |
| HTR4 | Yes | x | x |
| HTR5A | Yes | x | x |
| HTR6 | Yes | x | x |
| HTR7 | Yes | x | x |
| HUS1 | Yes | x | x |
| HYKK | Yes | x | x |
| IAPP | Yes | x | x |
| IDH1 | Yes | x | x |
| IDH3G | Yes | x | x |
| IDI1 | Yes | x | x |
| IDO1 | Yes | x | x |
| IDO2 | Yes | x | x |
| IFI27 | x | Yes | x |
| IFI30 | x | Yes | x |
| IFI44 | x | Yes | x |
| IFI44L | x | Yes | x |
| IFI6 | x | Yes | x |
| IFIH1 | x | Yes | x |
| IFIT1 | x | Yes | x |
| IFIT2 | x | Yes | x |
| IFIT3 | x | Yes | x |
| IFIT5 | x | Yes | x |
| IFITM1 | x | Yes | x |
| IFITM2 | x | Yes | x |
| IFITM3 | x | Yes | x |
| IFNG | Yes | x | x |
| IGF1R | Yes | x | x |
| IGF2 | Yes | x | x |
| IGF2R | Yes | x | x |
| IGFBP1 | Yes | x | x |
| IGFBP3 | Yes | x | x |
| IGFBP4 | Yes | x | x |
| IGFBP5 | Yes | x | x |
| IGFBP7 | Yes | x | x |
| IGLL5 | Yes | x | x |
| IHH | Yes | x | x |
| IL16 | Yes | x | x |
| IL2 | Yes | x | x |
| IL2RA | Yes | x | x |
| IL4 | Yes | x | x |
| IL6 | Yes | x | x |
| IL7R | Yes | x | x |
| ILF2 | Yes | x | x |
| IMMP1L | Yes | x | x |
| IMP3 | Yes | x | x |
| IMPDH1 | Yes | x | x |
| INMT | Yes | x | x |
| INPP5A | x | Yes | x |
| INPP5D | Yes | x | x |
| INS | Yes | x | x |
| INSIG1 | Yes | x | x |
| INSL3 | Yes | x | x |
| INSL5 | Yes | x | x |
| INTS3 | Yes | x | x |
| INVS | Yes | x | x |
| IP6K2 | x | Yes | x |
| IQGAP2 | Yes | x | x |
| IQSEC1 | Yes | x | x |
| IRF1 | x | Yes | x |
| IRF2 | x | Yes | x |
| IRF3 | x | Yes | x |
| IRF4 | x | Yes | x |
| IRF5 | x | Yes | x |
| IRF6 | x | Yes | x |
| IRF7 | x | Yes | x |
| IRF8 | x | Yes | x |
| IRF9 | x | Yes | x |
| IRX2 | Yes | x | x |
| IRX3 | Yes | x | x |
| IRX4 | Yes | x | x |
| ISG15 | x | Yes | x |
| ISG20 | x | Yes | x |
| IST1 | Yes | x | x |
| ITCH | Yes | x | x |
| ITGA1 | Yes | x | x |
| ITGA10 | Yes | x | x |
| ITGA2 | Yes | x | x |
| ITGA4 | Yes | x | x |
| ITGA5 | Yes | x | x |
| ITGA6 | Yes | x | x |
| ITGA8 | Yes | x | x |
| ITGA9 | Yes | x | x |
| ITGAD | Yes | x | x |
| ITGAE | Yes | x | x |
| ITGAL | Yes | x | x |
| ITGAM | Yes | x | x |
| ITGAV | Yes | x | x |
| ITGAX | Yes | x | x |
| ITGB1 | Yes | x | x |
| ITGB3 | Yes | x | x |
| ITGB4 | Yes | x | x |
| ITGB5 | Yes | x | x |
| ITGB6 | Yes | x | x |
| ITGB7 | Yes | x | x |
| ITGB8 | Yes | x | x |
| ITIH2 | Yes | x | x |
| ITIH4 | Yes | x | x |
| ITK | Yes | x | x |
| ITPRID1 | Yes | x | x |
| ITSN1 | Yes | x | x |
| ITSN2 | Yes | x | x |
| IZUMO1R | Yes | x | x |
| JAM2 | Yes | x | x |
| JCHAIN | Yes | x | x |
| JUP | Yes | x | x |
| KAT5 | Yes | x | x |
| KCNE3 | x | Yes | x |
| KCNG3 | x | Yes | x |
| KCNJ1 | Yes | x | x |
| KCNJ5 | x | Yes | x |
| KCNN4 | Yes | x | x |
| KCNQ2 | x | Yes | x |
| KCTD1 | Yes | x | x |
| KCTD11 | x | Yes | x |
| KCTD15 | Yes | x | x |
| KCTD3 | Yes | x | x |
| KDELR1 | Yes | x | x |
| KDELR2 | Yes | x | x |
| KDELR3 | Yes | x | x |
| KDM1A | Yes | x | x |
| KIAA0319 | Yes | x | x |
| KIAA0319L | Yes | x | x |
| KIAA0513 | x | Yes | x |
| KIAA1614 | x | Yes | x |
| KIF11 | Yes | x | x |
| KIF12 | Yes | x | x |
| KIF13A | Yes | x | x |
| KIF13B | Yes | x | x |
| KIF15 | Yes | x | x |
| KIF16B | Yes | x | x |
| KIF18A | Yes | x | x |
| KIF18B | Yes | x | x |
| KIF19 | Yes | x | x |
| KIF1A | Yes | x | x |
| KIF1B | Yes | x | x |
| KIF1C | Yes | x | x |
| KIF20A | Yes | x | x |
| KIF20B | Yes | x | x |
| KIF21A | Yes | x | x |
| KIF21B | Yes | x | x |
| KIF22 | Yes | x | x |
| KIF23 | Yes | x | x |
| KIF25 | Yes | x | x |
| KIF26A | Yes | x | x |
| KIF27 | Yes | x | x |
| KIF2A | Yes | x | x |
| KIF2B | Yes | x | x |
| KIF2C | Yes | x | x |
| KIF3A | Yes | x | x |
| KIF3B | Yes | x | x |
| KIF3C | Yes | x | x |
| KIF4A | Yes | x | x |
| KIF4B | Yes | x | x |
| KIF5A | Yes | x | x |
| KIF5B | Yes | x | x |
| KIF6 | Yes | x | x |
| KIF9 | Yes | x | x |
| KIFAP3 | Yes | x | x |
| KIFC1 | Yes | x | x |
| KIFC2 | Yes | x | x |
| KLC1 | Yes | x | x |
| KLC2 | Yes | x | x |
| KLC3 | Yes | x | x |
| KLC4 | Yes | x | x |
| KLHL12 | Yes | x | x |
| KLRC2 | Yes | x | x |
| KNG1 | Yes | x | x |
| KPNB1 | Yes | x | x |
| KRAS | Yes | x | x |
| KRT1 | Yes | x | x |
| KRT10 | Yes | x | x |
| KRT12 | Yes | x | x |
| KRT13 | Yes | x | x |
| KRT14 | Yes | x | x |
| KRT15 | Yes | x | x |
| KRT16 | Yes | x | x |
| KRT17 | Yes | x | x |
| KRT18 | Yes | x | x |
| KRT19 | Yes | x | x |
| KRT2 | Yes | x | x |
| KRT20 | Yes | x | x |
| KRT23 | Yes | x | x |
| KRT24 | Yes | x | x |
| KRT25 | Yes | x | x |
| KRT26 | Yes | x | x |
| KRT27 | Yes | x | x |
| KRT28 | Yes | x | x |
| KRT3 | Yes | x | x |
| KRT31 | Yes | x | x |
| KRT32 | Yes | x | x |
| KRT33A | Yes | x | x |
| KRT33B | Yes | x | x |
| KRT34 | Yes | x | x |
| KRT35 | Yes | x | x |
| KRT36 | Yes | x | x |
| KRT37 | Yes | x | x |
| KRT38 | Yes | x | x |
| KRT39 | Yes | x | x |
| KRT40 | Yes | x | x |
| KRT5 | Yes | x | x |
| KRT6A | Yes | x | x |
| KRT6B | Yes | x | x |
| KRT6C | Yes | x | x |
| KRT7 | Yes | x | x |
| KRT71 | Yes | x | x |
| KRT72 | Yes | x | x |
| KRT73 | Yes | x | x |
| KRT74 | Yes | x | x |
| KRT75 | Yes | x | x |
| KRT76 | Yes | x | x |
| KRT77 | Yes | x | x |
| KRT78 | Yes | x | x |
| KRT79 | Yes | x | x |
| KRT8 | Yes | x | x |
| KRT80 | Yes | x | x |
| KRT81 | Yes | x | x |
| KRT82 | Yes | x | x |
| KRT83 | Yes | x | x |
| KRT84 | Yes | x | x |
| KRT85 | Yes | x | x |
| KRT86 | Yes | x | x |
| KRT9 | Yes | x | x |
| KSR1 | Yes | x | x |
| KTN1 | Yes | x | x |
| L1CAM | Yes | x | x |
| L1TD1 | x | Yes | x |
| LAMA1 | Yes | x | x |
| LAMA2 | Yes | x | x |
| LAMA3 | Yes | x | x |
| LAMA4 | Yes | x | x |
| LAMA5 | Yes | x | x |
| LAMB1 | Yes | x | x |
| LAMB2 | Yes | x | x |
| LAMB3 | Yes | x | x |
| LAMC1 | Yes | x | x |
| LAMC2 | Yes | x | x |
| LAMC3 | Yes | x | x |
| LAMTOR1 | Yes | x | x |
| LAMTOR2 | Yes | x | x |
| LAMTOR3 | Yes | x | x |
| LAMTOR4 | Yes | x | x |
| LAMTOR5 | Yes | x | x |
| LARS1 | Yes | x | x |
| LAT2 | Yes | x | x |
| LATS1 | Yes | x | x |
| LATS2 | Yes | x | x |
| LBR | Yes | x | x |
| LCE1C | x | Yes | x |
| LCK | Yes | x | x |
| LCN2 | Yes | x | x |
| LCP2 | Yes | x | x |
| LDHD | Yes | x | x |
| LDLR | Yes | x | x |
| LDLRAP1 | Yes | x | x |
| LGALS1 | Yes | x | x |
| LGI1 | Yes | x | x |
| LGMN | Yes | x | x |
| LHB | Yes | x | x |
| LHCGR | Yes | x | x |
| LHX8 | Yes | x | x |
| LIG1 | Yes | x | x |
| LIG4 | Yes | x | x |
| LIMD1 | Yes | x | x |
| LIN7A | Yes | x | x |
| LINC02210-CRHR1 | Yes | x | x |
| LINGO1 | Yes | x | x |
| LLGL2 | x | Yes | x |
| LMAN1 | Yes | x | x |
| LMAN1L | Yes | x | x |
| LMAN2 | Yes | x | x |
| LMAN2L | Yes | x | x |
| LMF1 | x | Yes | x |
| LMTK2 | x | Yes | x |
| LOC100653049 | Yes | x | x |
| LOC102724334 | Yes | x | x |
| LOC102724560 | Yes | x | x |
| LPAR1 | Yes | x | x |
| LPAR2 | Yes | x | x |
| LPAR3 | Yes | x | x |
| LPAR5 | Yes | x | x |
| LRG1 | Yes | x | x |
| LRP2 | Yes | x | x |
| LRP5 | Yes | x | x |
| LRP6 | Yes | x | x |
| LRP8 | Yes | x | x |
| LRRC40 | Yes | x | x |
| LRRC7 | Yes | x | x |
| LSAMP | Yes | x | x |
| LSM3 | Yes | x | x |
| LSS | Yes | x | x |
| LTA | Yes | x | x |
| LTA4H | Yes | x | x |
| LTB | Yes | x | x |
| LTB4R2 | Yes | x | x |
| LTBP1 | Yes | x | x |
| LTBR | Yes | x | x |
| LTF | Yes | x | x |
| LTV1 | Yes | x | x |
| LUM | Yes | x | x |
| LY6D | Yes | x | x |
| LY6E | Yes | x | x |
| LY6G6C | Yes | x | x |
| LY6G6D | Yes | x | x |
| LY6H | Yes | x | x |
| LY6K | Yes | x | x |
| LYNX1 | x | Yes | x |
| LYPD1 | Yes | x | x |
| LYPD2 | Yes | x | x |
| LYPD3 | Yes | x | x |
| LYPD4 | Yes | x | x |
| LYPD5 | Yes | x | x |
| LYPD8 | Yes | x | x |
| LYZ | Yes | x | x |
| M6PR | Yes | x | x |
| MAD2L1 | Yes | x | x |
| MAEA | Yes | x | x |
| MAG | Yes | x | x |
| MAN2A1 | Yes | x | x |
| MAN2B1 | Yes | x | x |
| MAOA | Yes | x | x |
| MAOB | Yes | x | x |
| MAP1LC3A | Yes | x | x |
| MAP1LC3B | Yes | x | x |
| MAP1LC3C | Yes | x | x |
| MAP2K6 | x | Yes | x |
| MAP3K1 | Yes | x | x |
| MAP3K11 | Yes | x | x |
| MAP3K14 | Yes | x | x |
| MAP3K5 | Yes | x | x |
| MAP3K7 | Yes | x | x |
| MAP4K1 | Yes | x | x |
| MAP4K5 | Yes | x | x |
| MAPK1 | Yes | x | x |
| MAPK10 | Yes | x | x |
| MAPK11 | Yes | x | x |
| MAPK12 | Yes | x | x |
| MAPK13 | Yes | x | x |
| MAPK14 | Yes | x | x |
| MAPK3 | Yes | x | x |
| MAPK6 | Yes | x | x |
| MAPK8 | Yes | x | x |
| MAPK9 | Yes | x | x |
| MAPT | Yes | x | x |
| MATN3 | Yes | x | x |
| MBL2 | x | Yes | x |
| MBTPS1 | Yes | x | x |
| MC1R | Yes | x | x |
| MC2R | Yes | x | x |
| MC3R | Yes | x | x |
| MC4R | Yes | x | x |
| MC5R | Yes | x | x |
| MCFD2 | Yes | x | x |
| MCHR1 | Yes | x | x |
| MCHR2 | Yes | x | x |
| MCM4 | Yes | x | x |
| MCM6 | Yes | x | x |
| MCMDC2 | Yes | x | x |
| MDC1 | Yes | x | x |
| MDGA1 | Yes | x | x |
| MDGA2 | Yes | x | x |
| MDM2 | Yes | x | x |
| MED17 | Yes | x | x |
| MEIS1 | Yes | x | x |
| MELTF | Yes | x | x |
| MEN1 | Yes | x | x |
| MEOX2 | x | Yes | x |
| MET | Yes | x | x |
| METTL1 | Yes | x | x |
| METTL13 | Yes | x | x |
| METTL7A | Yes | x | x |
| MFGE8 | Yes | x | x |
| MGAT4A | Yes | x | x |
| MIA3 | Yes | x | x |
| MICOS13 | Yes | x | x |
| MID1 | x | Yes | x |
| MIF | Yes | x | x |
| MKLN1 | Yes | x | x |
| MLH1 | Yes | x | x |
| MLH3 | Yes | x | x |
| MLLT10 | x | Yes | x |
| MLST8 | Yes | x | x |
| MMP2 | Yes | x | x |
| MMP3 | Yes | x | x |
| MMP7 | Yes | x | x |
| MMP8 | Yes | x | x |
| MMP9 | Yes | x | x |
| MNAT1 | Yes | x | x |
| MNDA | Yes | x | x |
| MOB1A | Yes | x | x |
| MOB1B | Yes | x | x |
| MORN1 | x | Yes | x |
| MOV10 | Yes | x | x |
| MPDU1 | Yes | x | x |
| MPHOSPH8 | Yes | x | x |
| MPND | Yes | x | x |
| MPO | Yes | x | x |
| MPPED2 | Yes | x | x |
| MRAS | Yes | x | x |
| MRE11 | Yes | x | x |
| MRI1 | Yes | x | x |
| MRPL1 | Yes | x | x |
| MRPL10 | Yes | x | x |
| MRPL11 | Yes | x | x |
| MRPL12 | Yes | x | x |
| MRPL13 | Yes | x | x |
| MRPL14 | Yes | x | x |
| MRPL15 | Yes | x | x |
| MRPL16 | Yes | x | x |
| MRPL17 | Yes | x | x |
| MRPL18 | Yes | x | x |
| MRPL19 | Yes | x | x |
| MRPL2 | Yes | x | x |
| MRPL20 | Yes | x | x |
| MRPL21 | Yes | x | x |
| MRPL22 | Yes | x | x |
| MRPL23 | Yes | x | x |
| MRPL24 | Yes | x | x |
| MRPL27 | Yes | x | x |
| MRPL28 | Yes | x | x |
| MRPL3 | Yes | x | x |
| MRPL30 | Yes | x | x |
| MRPL32 | Yes | x | x |
| MRPL33 | Yes | x | x |
| MRPL34 | Yes | x | x |
| MRPL35 | Yes | x | x |
| MRPL36 | Yes | x | x |
| MRPL37 | Yes | x | x |
| MRPL4 | Yes | x | x |
| MRPL40 | Yes | x | x |
| MRPL41 | Yes | x | x |
| MRPL42 | Yes | x | x |
| MRPL43 | Yes | x | x |
| MRPL44 | Yes | x | x |
| MRPL46 | Yes | x | x |
| MRPL47 | Yes | x | x |
| MRPL48 | Yes | x | x |
| MRPL49 | Yes | x | x |
| MRPL51 | Yes | x | x |
| MRPL52 | Yes | x | x |
| MRPL53 | Yes | x | x |
| MRPL54 | Yes | x | x |
| MRPS10 | Yes | x | x |
| MRPS11 | Yes | x | x |
| MRPS12 | Yes | x | x |
| MRPS14 | Yes | x | x |
| MRPS15 | Yes | x | x |
| MRPS16 | Yes | x | x |
| MRPS17 | Yes | x | x |
| MRPS18A | Yes | x | x |
| MRPS18B | Yes | x | x |
| MRPS18C | Yes | x | x |
| MRPS2 | Yes | x | x |
| MRPS21 | Yes | x | x |
| MRPS22 | Yes | x | x |
| MRPS23 | Yes | x | x |
| MRPS24 | Yes | x | x |
| MRPS25 | Yes | x | x |
| MRPS26 | Yes | x | x |
| MRPS27 | Yes | x | x |
| MRPS28 | Yes | x | x |
| MRPS30 | Yes | x | x |
| MRPS31 | Yes | x | x |
| MRPS33 | Yes | x | x |
| MRPS34 | Yes | x | x |
| MRPS35 | Yes | x | x |
| MRPS36 | Yes | x | x |
| MRPS6 | Yes | x | x |
| MRPS7 | Yes | x | x |
| MRPS9 | Yes | x | x |
| MRRF | Yes | x | x |
| MRTO4 | Yes | x | x |
| MS4A12 | Yes | x | x |
| MSH2 | Yes | x | x |
| MSH3 | Yes | x | x |
| MSH6 | Yes | x | x |
| MSLN | Yes | x | x |
| MSMO1 | Yes | x | x |
| MSRA | x | Yes | x |
| MSTN | Yes | x | x |
| MT2A | x | Yes | x |
| MTAP | Yes | x | x |
| MTIF2 | Yes | x | x |
| MTIF3 | Yes | x | x |
| MTNR1A | Yes | x | x |
| MTNR1B | Yes | x | x |
| MTOR | Yes | x | x |
| MTRF1L | Yes | x | x |
| MUS81 | Yes | x | x |
| MVD | Yes | x | x |
| MVK | Yes | x | x |
| MX1 | x | Yes | x |
| MX2 | x | Yes | x |
| MXRA8 | Yes | x | x |
| MYC | Yes | x | x |
| MYH10 | Yes | x | x |
| MYH9 | Yes | x | x |
| MYO15A | Yes | x | x |
| MYO1C | Yes | x | x |
| MYO1E | Yes | x | x |
| MYO6 | Yes | x | x |
| MYO7A | Yes | x | x |
| MYO7B | Yes | x | x |
| MYOC | Yes | x | x |
| MYOF | x | Yes | x |
| MYOM2 | x | Yes | x |
| MYSM1 | Yes | x | x |
| MZB1 | Yes | x | x |
| NAGS | Yes | x | x |
| NANOS2 | Yes | x | x |
| NAPA | Yes | x | x |
| NAPB | Yes | x | x |
| NAPG | Yes | x | x |
| NAPRT | Yes | x | x |
| NBN | Yes | x | x |
| NBR1 | Yes | x | x |
| NCAM1 | x | Yes | x |
| NCAN | Yes | x | x |
| NCAPH | x | Yes | x |
| NCK1 | Yes | x | x |
| NCK2 | Yes | x | x |
| NCOA3 | Yes | x | x |
| ND1 | Yes | x | x |
| ND2 | Yes | x | x |
| ND3 | Yes | x | x |
| ND4 | Yes | x | x |
| ND5 | Yes | x | x |
| ND6 | Yes | x | x |
| NDUFA1 | Yes | x | x |
| NDUFA10 | Yes | x | x |
| NDUFA11 | Yes | x | x |
| NDUFA12 | Yes | x | x |
| NDUFA13 | Yes | x | x |
| NDUFA2 | Yes | x | x |
| NDUFA3 | Yes | x | x |
| NDUFA4 | Yes | x | x |
| NDUFA5 | Yes | x | x |
| NDUFA6 | Yes | x | x |
| NDUFA7 | Yes | x | x |
| NDUFA8 | Yes | x | x |
| NDUFA9 | Yes | x | x |
| NDUFAB1 | Yes | x | x |
| NDUFAF1 | Yes | x | x |
| NDUFAF2 | Yes | x | x |
| NDUFAF3 | Yes | x | x |
| NDUFAF4 | Yes | x | x |
| NDUFAF5 | Yes | x | x |
| NDUFAF6 | Yes | x | x |
| NDUFAF7 | Yes | x | x |
| NDUFB1 | Yes | x | x |
| NDUFB10 | Yes | x | x |
| NDUFB11 | Yes | x | x |
| NDUFB2 | Yes | x | x |
| NDUFB3 | Yes | x | x |
| NDUFB4 | Yes | x | x |
| NDUFB5 | Yes | x | x |
| NDUFB6 | Yes | x | x |
| NDUFB7 | Yes | x | x |
| NDUFB8 | Yes | x | x |
| NDUFB9 | Yes | x | x |
| NDUFC1 | Yes | x | x |
| NDUFC2 | Yes | x | x |
| NDUFS1 | Yes | x | x |
| NDUFS2 | Yes | x | x |
| NDUFS3 | Yes | x | x |
| NDUFS4 | Yes | x | x |
| NDUFS5 | Yes | x | x |
| NDUFS6 | Yes | x | x |
| NDUFS7 | Yes | x | x |
| NDUFS8 | Yes | x | x |
| NDUFV1 | Yes | x | x |
| NDUFV2 | Yes | x | x |
| NDUFV3 | Yes | x | x |
| NECAP1 | Yes | x | x |
| NECAP2 | Yes | x | x |
| NEDD8 | Yes | x | x |
| NEGR1 | Yes | x | x |
| NEU1 | Yes | x | x |
| NF1 | Yes | x | x |
| NFIL3 | Yes | x | x |
| NFKB1 | Yes | x | x |
| NFKB2 | Yes | x | x |
| NFKBIA | Yes | x | x |
| NFKBIB | Yes | x | x |
| NFKBIE | Yes | x | x |
| NFYA | Yes | x | x |
| NFYB | Yes | x | x |
| NFYC | Yes | x | x |
| NGFR | Yes | x | x |
| NHEJ1 | Yes | x | x |
| NHLRC3 | Yes | x | x |
| NID1 | Yes | x | x |
| NIT2 | Yes | x | x |
| NKAIN4 | x | Yes | x |
| NKD1 | Yes | x | x |
| NKD2 | Yes | x | x |
| NMNAT3 | x | Yes | x |
| NMS | Yes | x | x |
| NMU | Yes | x | x |
| NMUR1 | Yes | x | x |
| NMUR2 | Yes | x | x |
| NOB1 | Yes | x | x |
| NOS1 | Yes | x | x |
| NOS2 | Yes | x | x |
| NOS3 | Yes | x | x |
| NOTCH1 | Yes | x | x |
| NOTCH2 | Yes | x | x |
| NOTCH3 | Yes | x | x |
| NOTCH4 | Yes | x | x |
| NOTUM | Yes | x | x |
| NPAS2 | Yes | x | x |
| NPB | Yes | x | x |
| NPBWR1 | Yes | x | x |
| NPBWR2 | Yes | x | x |
| NPC2 | Yes | x | x |
| NPS | Yes | x | x |
| NPSR1 | Yes | x | x |
| NPW | Yes | x | x |
| NPY | Yes | x | x |
| NPY1R | Yes | x | x |
| NPY2R | Yes | x | x |
| NPY4R | Yes | x | x |
| NPY4R2 | Yes | x | x |
| NPY5R | Yes | x | x |
| NR1D1 | Yes | x | x |
| NR1D2 | Yes | x | x |
| NR3C1 | Yes | x | x |
| NRAS | Yes | x | x |
| NRIP1 | Yes | x | x |
| NRN1 | Yes | x | x |
| NRN1L | Yes | x | x |
| NRP1 | Yes | x | x |
| NRP2 | Yes | x | x |
| NRXN1 | Yes | x | x |
| NRXN2 | x | Yes | x |
| NSA2 | Yes | x | x |
| NSD2 | Yes | x | x |
| NSD3 | Yes | x | x |
| NSDHL | Yes | x | x |
| NSUN2 | Yes | x | x |
| NSUN3 | Yes | x | x |
| NTHL1 | Yes | x | x |
| NTN1 | Yes | x | x |
| NTNG1 | Yes | x | x |
| NTNG2 | Yes | x | x |
| NUBPL | Yes | x | x |
| NUCB1 | Yes | x | x |
| NUMB | Yes | x | x |
| NUP43 | Yes | x | x |
| NXN | x | Yes | x |
| OAS1 | x | Yes | x |
| OAS2 | x | Yes | x |
| OAS3 | x | Yes | x |
| OASL | x | Yes | x |
| OAT | Yes | x | x |
| OAZ1 | Yes | x | x |
| OAZ2 | Yes | x | x |
| OAZ3 | Yes | x | x |
| OCRL | Yes | x | x |
| ODC1 | Yes | x | x |
| OGG1 | Yes | x | x |
| OLFM2 | Yes | x | x |
| OLFM4 | Yes | x | x |
| OMG | Yes | x | x |
| OPCML | Yes | x | x |
| OPLAH | Yes | x | x |
| OPN4 | Yes | x | x |
| OPRD1 | Yes | x | x |
| OPRK1 | Yes | x | x |
| OPRL1 | Yes | x | x |
| OPRM1 | Yes | x | x |
| OPTN | Yes | x | x |
| OR2T11 | x | Yes | x |
| OR2T34 | x | Yes | x |
| ORC1 | Yes | x | x |
| ORM1 | Yes | x | x |
| ORM2 | Yes | x | x |
| OS9 | Yes | x | x |
| OSBPL7 | Yes | x | x |
| OSCAR | Yes | x | x |
| OTC | Yes | x | x |
| OTOA | Yes | x | x |
| OTOG | Yes | x | x |
| OXA1L | Yes | x | x |
| OXER1 | Yes | x | x |
| OXGR1 | Yes | x | x |
| P2RY12 | Yes | x | x |
| P2RY13 | Yes | x | x |
| P2RY14 | Yes | x | x |
| P2RY4 | Yes | x | x |
| P4HB | Yes | x | x |
| PA2G4 | Yes | x | x |
| PACSIN1 | Yes | x | x |
| PACSIN2 | Yes | x | x |
| PACSIN3 | Yes | x | x |
| PADI2 | Yes | x | x |
| PAG1 | Yes | x | x |
| PAK1 | Yes | x | x |
| PAK2 | Yes | x | x |
| PAK3 | Yes | x | x |
| PALLD | x | Yes | x |
| PAM16 | Yes | x | x |
| PARD6A | Yes | x | x |
| PARP1 | Yes | x | x |
| PARP12 | x | Yes | x |
| PARP2 | Yes | x | x |
| PARVA | Yes | x | x |
| PAXX | Yes | x | x |
| PCDH15 | Yes | x | x |
| PCDHA10 | x | Yes | x |
| PCLAF | Yes | x | x |
| PCNA | Yes | x | x |
| PCP2 | Yes | x | x |
| PCSK6 | x | Yes | x |
| PCSK9 | Yes | x | x |
| PDCD1 | Yes | x | x |
| PDCD11 | Yes | x | x |
| PDCD1LG2 | Yes | x | x |
| PDE4D | x | Yes | x |
| PDE8A | x | Yes | x |
| PDF | Yes | x | x |
| PDHA1 | Yes | x | x |
| PDHA2 | Yes | x | x |
| PDHB | Yes | x | x |
| PDHX | Yes | x | x |
| PDIA3 | Yes | x | x |
| PDIA6 | Yes | x | x |
| PDK1 | Yes | x | x |
| PDK2 | Yes | x | x |
| PDK3 | Yes | x | x |
| PDK4 | Yes | x | x |
| PDP2 | Yes | x | x |
| PDRG1 | Yes | x | x |
| PDXK | Yes | x | x |
| PDYN | Yes | x | x |
| PDZK1 | Yes | x | x |
| PENK | Yes | x | x |
| PER1 | Yes | x | x |
| PER2 | Yes | x | x |
| PER3 | Yes | x | x |
| PET117 | Yes | x | x |
| PF4 | Yes | x | x |
| PFDN2 | Yes | x | x |
| PFDN5 | Yes | x | x |
| PFDN6 | Yes | x | x |
| PFN1 | Yes | x | x |
| PGLYRP1 | Yes | x | x |
| PGM1 | Yes | x | x |
| PGR | Yes | x | x |
| PGRMC1 | Yes | x | x |
| PHC2 | x | Yes | x |
| PHF21A | Yes | x | x |
| PHLPP1 | Yes | x | x |
| PHYH | Yes | x | x |
| PI4K2A | Yes | x | x |
| PIAS4 | Yes | x | x |
| PICALM | Yes | x | x |
| PIGA | Yes | x | x |
| PIGB | Yes | x | x |
| PIGC | Yes | x | x |
| PIGG | Yes | x | x |
| PIGH | Yes | x | x |
| PIGK | Yes | x | x |
| PIGL | Yes | x | x |
| PIGM | Yes | x | x |
| PIGN | Yes | x | x |
| PIGO | Yes | x | x |
| PIGP | Yes | x | x |
| PIGQ | Yes | x | x |
| PIGV | Yes | x | x |
| PIGW | Yes | x | x |
| PIGY | Yes | x | x |
| PIH1D1 | Yes | x | x |
| PIK3C2A | Yes | x | x |
| PIK3C3 | Yes | x | x |
| PIK3CA | Yes | x | x |
| PIK3R1 | Yes | x | x |
| PIK3R2 | Yes | x | x |
| PIK3R4 | Yes | x | x |
| PINK1 | Yes | x | x |
| PIP5K1B | Yes | x | x |
| PISD | Yes | x | x |
| PITRM1 | Yes | x | x |
| PLA1A | x | Yes | x |
| PLAC8 | Yes | x | x |
| PLAT | Yes | x | x |
| PLAU | Yes | x | x |
| PLAUR | Yes | x | x |
| PLCG2 | Yes | x | x |
| PLD1 | Yes | x | x |
| PLD2 | Yes | x | x |
| PLEC | Yes | x | x |
| PLEK | Yes | x | x |
| PLEKHG4B | x | Yes | x |
| PLET1 | Yes | x | x |
| PLIN3 | Yes | x | x |
| PLK1 | Yes | x | x |
| PLXNA1 | Yes | x | x |
| PLXNA2 | Yes | x | x |
| PLXNA3 | Yes | x | x |
| PLXNA4 | Yes | x | x |
| PLXNB1 | Yes | x | x |
| PLXNB2 | Yes | x | x |
| PLXNB3 | Yes | x | x |
| PLXNC1 | Yes | x | x |
| PLXND1 | Yes | x | x |
| PMCH | Yes | x | x |
| PML | x | Yes | x |
| PMPCA | Yes | x | x |
| PMPCB | Yes | x | x |
| PMS1 | Yes | x | x |
| PMS2 | Yes | x | x |
| PMVK | Yes | x | x |
| PNLIP | Yes | x | x |
| PNLIPRP1 | Yes | x | x |
| PNMA2 | x | Yes | x |
| PNOC | Yes | x | x |
| PNPLA2 | Yes | x | x |
| PNPO | Yes | x | x |
| POLD1 | Yes | x | x |
| POLD2 | Yes | x | x |
| POLD3 | Yes | x | x |
| POLD4 | Yes | x | x |
| POLE2 | Yes | x | x |
| POLE3 | Yes | x | x |
| POLE4 | Yes | x | x |
| POLI | Yes | x | x |
| POLK | Yes | x | x |
| POLN | Yes | x | x |
| POLR1A | Yes | x | x |
| POLR1B | Yes | x | x |
| POLR2A | Yes | x | x |
| POLR2B | Yes | x | x |
| POLR2C | Yes | x | x |
| POLR2D | Yes | x | x |
| POLR2E | Yes | x | x |
| POLR2F | Yes | x | x |
| POLR2G | Yes | x | x |
| POLR2H | Yes | x | x |
| POLR2I | Yes | x | x |
| POLR2J | Yes | x | x |
| POLR2K | Yes | x | x |
| POLR2L | Yes | x | x |
| POMC | Yes | x | x |
| POMP | Yes | x | x |
| POR | Yes | x | x |
| POSTN | Yes | x | x |
| POU2AF1 | Yes | x | x |
| PPARGC1A | Yes | x | x |
| PPBP | Yes | x | x |
| PPID | Yes | x | x |
| PPIE | Yes | x | x |
| PPIL2 | x | Yes | x |
| PPP1CA | Yes | x | x |
| PPP1CB | Yes | x | x |
| PPP1CC | Yes | x | x |
| PPP2CA | Yes | x | x |
| PPP2CB | Yes | x | x |
| PPP2R1A | Yes | x | x |
| PPP2R1B | Yes | x | x |
| PPP2R2B | x | Yes | x |
| PPP2R2D | x | Yes | x |
| PPP2R3C | Yes | x | x |
| PPP2R5A | Yes | x | x |
| PPP2R5B | Yes | x | x |
| PPP2R5C | Yes | x | x |
| PPP2R5D | Yes | x | x |
| PPP2R5E | Yes | x | x |
| PPP5C | Yes | x | x |
| PPP5D1P | Yes | x | x |
| PPY | Yes | x | x |
| PRCP | x | Yes | x |
| PRDM16 | x | Yes | x |
| PRDX6 | Yes | x | x |
| PRG3 | Yes | x | x |
| PRICKLE1 | Yes | x | x |
| PRICKLE2 | Yes | x | x |
| PRICKLE3 | Yes | x | x |
| PRICKLE4 | Yes | x | x |
| PRIM2 | Yes | x | x |
| PRKAA1 | Yes | x | x |
| PRKAA2 | Yes | x | x |
| PRKAB1 | Yes | x | x |
| PRKAB2 | Yes | x | x |
| PRKACA | Yes | x | x |
| PRKACB | Yes | x | x |
| PRKACG | Yes | x | x |
| PRKAG1 | Yes | x | x |
| PRKAG3 | Yes | x | x |
| PRKAR1A | Yes | x | x |
| PRKAR1B | Yes | x | x |
| PRKAR2A | Yes | x | x |
| PRKAR2B | Yes | x | x |
| PRKCA | Yes | x | x |
| PRKCB | Yes | x | x |
| PRKCD | Yes | x | x |
| PRKCG | Yes | x | x |
| PRKCQ | Yes | x | x |
| PRKCSH | Yes | x | x |
| PRKDC | Yes | x | x |
| PRKG1 | Yes | x | x |
| PRND | Yes | x | x |
| PROC | Yes | x | x |
| PRPF19 | Yes | x | x |
| PRR12 | x | Yes | x |
| PRR15L | Yes | x | x |
| PRSS1 | Yes | x | x |
| PRSS2 | Yes | x | x |
| PRSS21 | Yes | x | x |
| PRSS23 | Yes | x | x |
| PRSS3 | Yes | x | x |
| PRTN3 | Yes | x | x |
| PSAP | Yes | x | x |
| PSD | Yes | x | x |
| PSMA1 | Yes | x | x |
| PSMA2 | Yes | x | x |
| PSMA3 | Yes | x | x |
| PSMA4 | Yes | x | x |
| PSMA5 | Yes | x | x |
| PSMA6 | Yes | x | x |
| PSMA7 | Yes | x | x |
| PSMA8 | Yes | x | x |
| PSMB1 | Yes | x | x |
| PSMB10 | Yes | x | x |
| PSMB11 | Yes | x | x |
| PSMB2 | Yes | x | x |
| PSMB3 | Yes | x | x |
| PSMB4 | Yes | x | x |
| PSMB5 | Yes | x | x |
| PSMB6 | Yes | x | x |
| PSMB7 | Yes | x | x |
| PSMB9 | Yes | x | x |
| PSMC1 | Yes | x | x |
| PSMC2 | Yes | x | x |
| PSMC3 | Yes | x | x |
| PSMC4 | Yes | x | x |
| PSMC5 | Yes | x | x |
| PSMC6 | Yes | x | x |
| PSMD1 | Yes | x | x |
| PSMD10 | Yes | x | x |
| PSMD11 | Yes | x | x |
| PSMD12 | Yes | x | x |
| PSMD14 | Yes | x | x |
| PSMD2 | Yes | x | x |
| PSMD3 | Yes | x | x |
| PSMD4 | Yes | x | x |
| PSMD5 | Yes | x | x |
| PSMD7 | Yes | x | x |
| PSMD8 | Yes | x | x |
| PSMD9 | Yes | x | x |
| PSME1 | Yes | x | x |
| PSME2 | Yes | x | x |
| PSME3 | Yes | x | x |
| PSME4 | Yes | x | x |
| PSMF1 | Yes | x | x |
| PSPC1 | Yes | x | x |
| PTAFR | x | Yes | x |
| PTCD3 | Yes | x | x |
| PTEN | Yes | x | x |
| PTF1A | Yes | x | x |
| PTGDR | Yes | x | x |
| PTGDR2 | Yes | x | x |
| PTGER2 | Yes | x | x |
| PTGER3 | Yes | x | x |
| PTGER4 | Yes | x | x |
| PTGES2 | Yes | x | x |
| PTGES3 | Yes | x | x |
| PTGIR | Yes | x | x |
| PTH | Yes | x | x |
| PTH1R | Yes | x | x |
| PTH2 | Yes | x | x |
| PTHLH | Yes | x | x |
| PTK2 | Yes | x | x |
| PTK2B | Yes | x | x |
| PTK7 | Yes | x | x |
| PTPN11 | Yes | x | x |
| PTPN22 | Yes | x | x |
| PTPN6 | Yes | x | x |
| PTPRC | Yes | x | x |
| PTPRJ | Yes | x | x |
| PTTG1 | Yes | x | x |
| PTX3 | Yes | x | x |
| PUS3 | Yes | x | x |
| PXN | Yes | x | x |
| PYCARD | Yes | x | x |
| PYGB | Yes | x | x |
| PYURF | Yes | x | x |
| PYY | Yes | x | x |
| QPCT | Yes | x | x |
| QPCTL | Yes | x | x |
| QSOX1 | Yes | x | x |
| RAB10 | Yes | x | x |
| RAB11A | Yes | x | x |
| RAB11FIP1 | Yes | x | x |
| RAB11FIP2 | Yes | x | x |
| RAB11FIP3 | Yes | x | x |
| RAB11FIP4 | Yes | x | x |
| RAB11FIP5 | Yes | x | x |
| RAB13 | Yes | x | x |
| RAB14 | Yes | x | x |
| RAB1A | Yes | x | x |
| RAB1B | Yes | x | x |
| RAB25 | x | Yes | x |
| RAB27A | Yes | x | x |
| RAB4A | Yes | x | x |
| RAB5A | Yes | x | x |
| RAB5B | Yes | x | x |
| RAB5C | Yes | x | x |
| RAB7A | Yes | x | x |
| RAB7B | Yes | x | x |
| RAB8A | Yes | x | x |
| RAB9A | Yes | x | x |
| RAB9B | Yes | x | x |
| RABEPK | Yes | x | x |
| RABIF | Yes | x | x |
| RAC1 | Yes | x | x |
| RAC2 | Yes | x | x |
| RAC3 | Yes | x | x |
| RACGAP1 | Yes | x | x |
| RACK1 | Yes | x | x |
| RAD1 | Yes | x | x |
| RAD17 | Yes | x | x |
| RAD23A | Yes | x | x |
| RAD23B | Yes | x | x |
| RAD50 | Yes | x | x |
| RAD51C | Yes | x | x |
| RAD52 | Yes | x | x |
| RAD9A | Yes | x | x |
| RAD9B | Yes | x | x |
| RAET1G | Yes | x | x |
| RAET1L | Yes | x | x |
| RAF1 | Yes | x | x |
| RALA | Yes | x | x |
| RALB | Yes | x | x |
| RALBP1 | Yes | x | x |
| RALGAPA2 | Yes | x | x |
| RALGAPB | Yes | x | x |
| RALGDS | Yes | x | x |
| RALGPS1 | Yes | x | x |
| RAMP1 | Yes | x | x |
| RAMP2 | Yes | x | x |
| RAMP3 | Yes | x | x |
| RAN | Yes | x | x |
| RANBP10 | Yes | x | x |
| RANBP9 | Yes | x | x |
| RAP1B | Yes | x | x |
| RARS1 | Yes | x | x |
| RB1CC1 | Yes | x | x |
| RBBP8 | Yes | x | x |
| RBM44 | Yes | x | x |
| RBM8A | Yes | x | x |
| RBX1 | Yes | x | x |
| RCHY1 | Yes | x | x |
| RCN1 | Yes | x | x |
| RCOR1 | Yes | x | x |
| RECK | Yes | x | x |
| REL | Yes | x | x |
| RELA | Yes | x | x |
| RELB | Yes | x | x |
| RELN | Yes | x | x |
| REPS1 | Yes | x | x |
| REPS2 | Yes | x | x |
| RETN | Yes | x | x |
| REV1 | Yes | x | x |
| REV3L | Yes | x | x |
| RFC1 | Yes | x | x |
| RFC2 | Yes | x | x |
| RFC3 | Yes | x | x |
| RFC4 | Yes | x | x |
| RFC5 | Yes | x | x |
| RGL1 | Yes | x | x |
| RGL2 | Yes | x | x |
| RGS1 | Yes | x | x |
| RGS14 | x | Yes | x |
| RHBDD1 | Yes | x | x |
| RHBDD2 | Yes | x | x |
| RHBDF1 | Yes | x | x |
| RHEB | Yes | x | x |
| RHNO1 | Yes | x | x |
| RHOA | Yes | x | x |
| RHOB | Yes | x | x |
| RHOBTB1 | Yes | x | x |
| RHOBTB2 | Yes | x | x |
| RHOBTB3 | Yes | x | x |
| RHOC | Yes | x | x |
| RHOD | Yes | x | x |
| RHOF | Yes | x | x |
| RHOG | Yes | x | x |
| RHOH | Yes | x | x |
| RHOJ | Yes | x | x |
| RHOQ | Yes | x | x |
| RHOT1 | Yes | x | x |
| RHOT2 | Yes | x | x |
| RHOU | Yes | x | x |
| RHOV | Yes | x | x |
| RLN2 | Yes | x | x |
| RLN3 | Yes | x | x |
| RMI1 | Yes | x | x |
| RMI2 | Yes | x | x |
| RMND5B | Yes | x | x |
| RNASE2 | Yes | x | x |
| RNASE3 | Yes | x | x |
| RNASEL | x | Yes | x |
| RNASET2 | Yes | x | x |
| RNF146 | Yes | x | x |
| RNF169 | x | Yes | x |
| RNF185 | Yes | x | x |
| RNF213 | x | Yes | x |
| RNF5 | Yes | x | x |
| RNF8 | Yes | x | x |
| ROR2 | Yes | x | x |
| RORA | Yes | x | x |
| RORC | Yes | x | x |
| RPA1 | Yes | x | x |
| RPA2 | Yes | x | x |
| RPA3 | Yes | x | x |
| RPAP1 | Yes | x | x |
| RPAP2 | Yes | x | x |
| RPAP3 | Yes | x | x |
| RPH3AL | x | Yes | x |
| RPL11 | Yes | x | x |
| RPL12 | Yes | x | x |
| RPL13 | Yes | x | x |
| RPL13A | Yes | x | x |
| RPL14 | Yes | x | x |
| RPL15 | Yes | x | x |
| RPL18 | Yes | x | x |
| RPL19 | Yes | x | x |
| RPL21 | Yes | x | x |
| RPL22 | Yes | x | x |
| RPL22L1 | Yes | x | x |
| RPL23 | Yes | x | x |
| RPL24 | Yes | x | x |
| RPL26 | Yes | x | x |
| RPL26L1 | Yes | x | x |
| RPL27 | Yes | x | x |
| RPL27A | Yes | x | x |
| RPL29 | Yes | x | x |
| RPL3 | Yes | x | x |
| RPL30 | Yes | x | x |
| RPL31 | Yes | x | x |
| RPL32 | Yes | x | x |
| RPL34 | Yes | x | x |
| RPL35 | Yes | x | x |
| RPL35A | Yes | x | x |
| RPL36 | Yes | x | x |
| RPL36A | Yes | x | x |
| RPL37A | Yes | x | x |
| RPL38 | Yes | x | x |
| RPL4 | Yes | x | x |
| RPL6 | Yes | x | x |
| RPL7 | Yes | x | x |
| RPL8 | Yes | x | x |
| RPLP0 | Yes | x | x |
| RPLP1 | Yes | x | x |
| RPLP2 | Yes | x | x |
| RPN1 | Yes | x | x |
| RPN2 | Yes | x | x |
| RPS10 | Yes | x | x |
| RPS10-NUDT3 | Yes | x | x |
| RPS14 | Yes | x | x |
| RPS15 | Yes | x | x |
| RPS15A | Yes | x | x |
| RPS17 | Yes | x | x |
| RPS18 | Yes | x | x |
| RPS19 | Yes | x | x |
| RPS2 | Yes | x | x |
| RPS20 | Yes | x | x |
| RPS21 | Yes | x | x |
| RPS23 | Yes | x | x |
| RPS24 | Yes | x | x |
| RPS25 | Yes | x | x |
| RPS26 | Yes | x | x |
| RPS27 | Yes | x | x |
| RPS27A | Yes | x | x |
| RPS27L | Yes | x | x |
| RPS28 | Yes | x | x |
| RPS29 | Yes | x | x |
| RPS3 | Yes | x | x |
| RPS3A | Yes | x | x |
| RPS5 | Yes | x | x |
| RPS6KA1 | Yes | x | x |
| RPS8 | Yes | x | x |
| RPS9 | Yes | x | x |
| RPTOR | Yes | x | x |
| RRAGA | Yes | x | x |
| RRAGB | Yes | x | x |
| RRAGC | Yes | x | x |
| RRAS | Yes | x | x |
| RRAS2 | Yes | x | x |
| RRM1 | Yes | x | x |
| RSAD2 | x | Yes | x |
| RSL24D1 | Yes | x | x |
| RTF1 | Yes | x | x |
| RTF2 | Yes | x | x |
| RTN1 | Yes | x | x |
| RTN4 | Yes | x | x |
| RTN4R | Yes | x | x |
| RTN4RL1 | Yes | x | x |
| RTN4RL2 | Yes | x | x |
| RTP4 | x | Yes | x |
| RUNX3 | Yes | x | x |
| RUVBL1 | Yes | x | x |
| RUVBL2 | Yes | x | x |
| RXFP1 | Yes | x | x |
| RXFP2 | Yes | x | x |
| RXFP3 | Yes | x | x |
| RXFP4 | Yes | x | x |
| RYR1 | Yes | x | x |
| S100A1 | Yes | x | x |
| S100A7 | Yes | x | x |
| S1PR1 | Yes | x | x |
| S1PR2 | Yes | x | x |
| S1PR3 | Yes | x | x |
| S1PR4 | Yes | x | x |
| S1PR5 | Yes | x | x |
| SAA1 | Yes | x | x |
| SAMD9L | x | Yes | x |
| SAMHD1 | x | Yes | x |
| SAR1B | Yes | x | x |
| SBF2 | x | Yes | x |
| SBK2 | x | Yes | x |
| SC5D | Yes | x | x |
| SCAMP1 | Yes | x | x |
| SCARA5 | x | Yes | x |
| SCARB2 | Yes | x | x |
| SCD5 | Yes | x | x |
| SCFD1 | Yes | x | x |
| SCG2 | Yes | x | x |
| SCG3 | Yes | x | x |
| SCNN1A | Yes | x | x |
| SCNN1B | Yes | x | x |
| SCNN1G | Yes | x | x |
| SCO1 | Yes | x | x |
| SCO2 | Yes | x | x |
| SCT | Yes | x | x |
| SCTR | Yes | x | x |
| SDC1 | Yes | x | x |
| SDC2 | Yes | x | x |
| SDC3 | Yes | x | x |
| SDC4 | Yes | x | x |
| SDCBP | Yes | x | x |
| SEC13 | Yes | x | x |
| SEC14L2 | Yes | x | x |
| SEC16A | Yes | x | x |
| SEC16B | Yes | x | x |
| SEC22A | Yes | x | x |
| SEC22B | Yes | x | x |
| SEC22C | Yes | x | x |
| SEC23A | Yes | x | x |
| SEC23IP | Yes | x | x |
| SEC24A | Yes | x | x |
| SEC24B | Yes | x | x |
| SEC24C | Yes | x | x |
| SEC24D | Yes | x | x |
| SEC31A | Yes | x | x |
| SEC61A1 | Yes | x | x |
| SEC61A2 | Yes | x | x |
| SEC61B | Yes | x | x |
| SEC61G | Yes | x | x |
| SEL1L | Yes | x | x |
| SELL | Yes | x | x |
| SEM1 | Yes | x | x |
| SEMA3F | Yes | x | x |
| SENP2 | Yes | x | x |
| SERPINA1 | Yes | x | x |
| SERPINA10 | Yes | x | x |
| SERPINB2 | x | Yes | x |
| SERPINB3 | Yes | x | x |
| SERPINB6 | x | Yes | x |
| SERPINB9 | x | Yes | x |
| SERPINC1 | Yes | x | x |
| SERPIND1 | Yes | x | x |
| SETD1A | Yes | x | x |
| SFMBT1 | x | Yes | x |
| SFRP2 | Yes | x | x |
| SH3BP2 | Yes | x | x |
| SH3GL1 | Yes | x | x |
| SH3GL2 | Yes | x | x |
| SH3GL3 | Yes | x | x |
| SH3KBP1 | Yes | x | x |
| SH3PXD2B | x | Yes | x |
| SHANK2 | x | Yes | x |
| SHC1 | Yes | x | x |
| SHH | Yes | x | x |
| SHISA5 | Yes | x | x |
| SHISA9 | Yes | x | x |
| SIAH3 | x | Yes | x |
| SIGMAR1 | Yes | x | x |
| SIRPA | Yes | x | x |
| SIRT1 | Yes | x | x |
| SKAP1 | Yes | x | x |
| SKAP2 | Yes | x | x |
| SKP1 | Yes | x | x |
| SKP2 | Yes | x | x |
| SLA2 | Yes | x | x |
| SLC12A2 | Yes | x | x |
| SLC15A2 | x | Yes | x |
| SLC18A3 | Yes | x | x |
| SLC19A1 | x | Yes | x |
| SLC25A10 | Yes | x | x |
| SLC25A4 | Yes | x | x |
| SLC26A3 | Yes | x | x |
| SLC26A6 | Yes | x | x |
| SLC26A8 | Yes | x | x |
| SLC26A9 | Yes | x | x |
| SLC2A4 | Yes | x | x |
| SLC2A8 | Yes | x | x |
| SLC35F3 | x | Yes | x |
| SLC38A9 | Yes | x | x |
| SLC39A14 | x | Yes | x |
| SLC3A1 | Yes | x | x |
| SLC3A2 | Yes | x | x |
| SLC4A4 | Yes | x | x |
| SLC6A19 | Yes | x | x |
| SLC6A20 | Yes | x | x |
| SLC6A6 | x | Yes | x |
| SLC7A1 | x | Yes | x |
| SLC7A9 | Yes | x | x |
| SLC9A2 | x | Yes | x |
| SLC9A3 | Yes | x | x |
| SLC9A3R1 | Yes | x | x |
| SLC9A3R2 | Yes | x | x |
| SLCO2B1 | x | Yes | x |
| SLIT3 | x | Yes | x |
| SLPI | Yes | x | x |
| SLX1A | Yes | x | x |
| SLX1B | Yes | x | x |
| SLX4 | Yes | x | x |
| SMAD6 | Yes | x | x |
| SMCO2 | x | Yes | x |
| SMCR8 | Yes | x | x |
| SMOC1 | x | Yes | x |
| SMURF1 | Yes | x | x |
| SMURF2 | Yes | x | x |
| SMYD3 | x | Yes | x |
| SNAP23 | Yes | x | x |
| SNAP25 | Yes | x | x |
| SNAP47 | Yes | x | x |
| SNAP91 | Yes | x | x |
| SNPH | x | Yes | x |
| SNRNP25 | x | Yes | x |
| SNRNP40 | x | Yes | x |
| SNRPG | Yes | x | x |
| SNX18 | Yes | x | x |
| SNX3 | Yes | x | x |
| SNX4 | Yes | x | x |
| SNX6 | x | Yes | x |
| SNX9 | Yes | x | x |
| SOD2 | Yes | x | x |
| SOHLH1 | Yes | x | x |
| SORT1 | Yes | x | x |
| SOS1 | Yes | x | x |
| SP1 | Yes | x | x |
| SP100 | x | Yes | x |
| SP6 | x | Yes | x |
| SPA17 | Yes | x | x |
| SPACA4 | Yes | x | x |
| SPARC | Yes | x | x |
| SPARCL1 | Yes | x | x |
| SPATA4 | Yes | x | x |
| SPG7 | Yes | x | x |
| SPINK1 | Yes | x | x |
| SPOP | Yes | x | x |
| SPOPL | Yes | x | x |
| SPP1 | Yes | x | x |
| SPP2 | Yes | x | x |
| SPRED1 | Yes | x | x |
| SPRED2 | Yes | x | x |
| SPRED3 | Yes | x | x |
| SPRN | Yes | x | x |
| SPTA1 | Yes | x | x |
| SPTAN1 | Yes | x | x |
| SPTB | Yes | x | x |
| SPTBN1 | Yes | x | x |
| SPTBN2 | Yes | x | x |
| SPTBN4 | Yes | x | x |
| SPTBN5 | Yes | x | x |
| SQLE | Yes | x | x |
| SQSTM1 | Yes | x | x |
| SRC | Yes | x | x |
| SREBF2 | Yes | x | x |
| SRM | Yes | x | x |
| SRP14 | Yes | x | x |
| SRP19 | Yes | x | x |
| SRP68 | Yes | x | x |
| SST | Yes | x | x |
| SSTR1 | Yes | x | x |
| SSTR2 | Yes | x | x |
| SSTR3 | Yes | x | x |
| SSTR4 | Yes | x | x |
| SSTR5 | Yes | x | x |
| ST3GAL5 | Yes | x | x |
| STAC3 | Yes | x | x |
| STAM | Yes | x | x |
| STAM2 | Yes | x | x |
| STAR | Yes | x | x |
| STARD3 | Yes | x | x |
| STARD3NL | Yes | x | x |
| STARD4 | Yes | x | x |
| STARD6 | Yes | x | x |
| STAT1 | x | Yes | x |
| STAT2 | x | Yes | x |
| STAT4 | Yes | x | x |
| STC2 | Yes | x | x |
| STIP1 | Yes | x | x |
| STK11IP | Yes | x | x |
| STON1 | Yes | x | x |
| STON1-GTF2A1L | Yes | x | x |
| STON2 | Yes | x | x |
| STRA8 | Yes | x | x |
| STUB1 | Yes | x | x |
| STX10 | Yes | x | x |
| STX11 | Yes | x | x |
| STX16 | Yes | x | x |
| STX17 | Yes | x | x |
| STX19 | Yes | x | x |
| STX1A | Yes | x | x |
| STX1B | Yes | x | x |
| STX3 | Yes | x | x |
| STX4 | Yes | x | x |
| STX5 | Yes | x | x |
| STX7 | Yes | x | x |
| STX8 | Yes | x | x |
| STXBP3 | Yes | x | x |
| SUCNR1 | Yes | x | x |
| SUFU | Yes | x | x |
| SUMO1 | Yes | x | x |
| SURF1 | Yes | x | x |
| SURF4 | Yes | x | x |
| SWSAP1 | Yes | x | x |
| SYK | Yes | x | x |
| SYNJ1 | Yes | x | x |
| SYNJ2 | Yes | x | x |
| SYPL1 | Yes | x | x |
| SYT1 | Yes | x | x |
| SYT11 | Yes | x | x |
| SYT13 | Yes | x | x |
| SYT2 | Yes | x | x |
| SYT8 | Yes | x | x |
| SYT9 | Yes | x | x |
| SYTL1 | Yes | x | x |
| TAAR1 | Yes | x | x |
| TAAR2 | Yes | x | x |
| TAAR5 | Yes | x | x |
| TAAR6 | Yes | x | x |
| TAAR8 | Yes | x | x |
| TAAR9 | Yes | x | x |
| TACO1 | Yes | x | x |
| TACR1 | Yes | x | x |
| TANC1 | x | Yes | x |
| TAPT1 | Yes | x | x |
| TARBP1 | Yes | x | x |
| TARDBP | Yes | x | x |
| TAS1R1 | Yes | x | x |
| TAS1R2 | Yes | x | x |
| TAS1R3 | Yes | x | x |
| TAS2R1 | Yes | x | x |
| TAS2R10 | Yes | x | x |
| TAS2R13 | Yes | x | x |
| TAS2R14 | Yes | x | x |
| TAS2R16 | Yes | x | x |
| TAS2R19 | Yes | x | x |
| TAS2R20 | Yes | x | x |
| TAS2R3 | Yes | x | x |
| TAS2R30 | Yes | x | x |
| TAS2R31 | Yes | x | x |
| TAS2R38 | Yes | x | x |
| TAS2R39 | Yes | x | x |
| TAS2R4 | Yes | x | x |
| TAS2R40 | Yes | x | x |
| TAS2R41 | Yes | x | x |
| TAS2R43 | Yes | x | x |
| TAS2R46 | Yes | x | x |
| TAS2R5 | Yes | x | x |
| TAS2R50 | Yes | x | x |
| TAS2R60 | Yes | x | x |
| TAS2R7 | Yes | x | x |
| TAS2R8 | Yes | x | x |
| TAS2R9 | Yes | x | x |
| TATDN1 | Yes | x | x |
| TAX1BP1 | Yes | x | x |
| TAX1BP3 | Yes | x | x |
| TBC1D15 | Yes | x | x |
| TBC1D17 | Yes | x | x |
| TBC1D25 | Yes | x | x |
| TBCD | x | Yes | x |
| TBKBP1 | Yes | x | x |
| TBL2 | Yes | x | x |
| TCEA1 | Yes | x | x |
| TCN1 | Yes | x | x |
| TCP1 | Yes | x | x |
| TDP1 | Yes | x | x |
| TEC | Yes | x | x |
| TECR | Yes | x | x |
| TECTA | Yes | x | x |
| TECTB | Yes | x | x |
| TEF | Yes | x | x |
| TELO2 | Yes | x | x |
| TEX101 | Yes | x | x |
| TF | Yes | x | x |
| TFAP2A | Yes | x | x |
| TFAP2B | Yes | x | x |
| TFAP2C | Yes | x | x |
| TFAP2D | Yes | x | x |
| TFAP2E | Yes | x | x |
| TFG | Yes | x | x |
| TFRC | Yes | x | x |
| TGFA | Yes | x | x |
| TGFB1 | Yes | x | x |
| TGFB2 | Yes | x | x |
| TGFB3 | Yes | x | x |
| TGFBI | Yes | x | x |
| TGFBR3 | x | Yes | x |
| TGOLN2 | Yes | x | x |
| THAP11 | Yes | x | x |
| THAP12 | Yes | x | x |
| THAP3 | Yes | x | x |
| THBS1 | Yes | x | x |
| THBS2 | Yes | x | x |
| THEMIS | Yes | x | x |
| THOC1 | Yes | x | x |
| THY1 | Yes | x | x |
| TIMELESS | Yes | x | x |
| TIMM17A | Yes | x | x |
| TIMM21 | Yes | x | x |
| TIMM22 | Yes | x | x |
| TIMM23 | Yes | x | x |
| TIMM44 | Yes | x | x |
| TIMM50 | Yes | x | x |
| TIMMDC1 | Yes | x | x |
| TIMP1 | Yes | x | x |
| TIMP2 | Yes | x | x |
| TJP1 | Yes | x | x |
| TLE3 | Yes | x | x |
| TLN1 | Yes | x | x |
| TLN2 | Yes | x | x |
| TLR2 | Yes | x | x |
| TLR4 | Yes | x | x |
| TM7SF2 | Yes | x | x |
| TMC7 | x | Yes | x |
| TMCO4 | x | Yes | x |
| TMED10 | Yes | x | x |
| TMED2 | Yes | x | x |
| TMED3 | Yes | x | x |
| TMED7 | Yes | x | x |
| TMED7-TICAM2 | Yes | x | x |
| TMED9 | Yes | x | x |
| TMEM126B | Yes | x | x |
| TMEM132A | Yes | x | x |
| TMEM176A | Yes | x | x |
| TMEM69 | Yes | x | x |
| TMIGD3 | Yes | x | x |
| TMPRSS3 | Yes | x | x |
| TMTC1 | x | Yes | x |
| TNC | Yes | x | x |
| TNF | Yes | x | x |
| TNFAIP6 | Yes | x | x |
| TNFRSF11A | Yes | x | x |
| TNFRSF12A | Yes | x | x |
| TNFRSF13B | Yes | x | x |
| TNFRSF13C | Yes | x | x |
| TNFRSF17 | Yes | x | x |
| TNFRSF19 | Yes | x | x |
| TNFRSF1A | Yes | x | x |
| TNFRSF1B | Yes | x | x |
| TNFSF11 | Yes | x | x |
| TNFSF12 | Yes | x | x |
| TNFSF12-TNFSF13 | Yes | x | x |
| TNFSF13 | Yes | x | x |
| TNFSF13B | Yes | x | x |
| TNFSF14 | Yes | x | x |
| TNKS2 | Yes | x | x |
| TOLLIP | Yes | x | x |
| TOM1L2 | Yes | x | x |
| TOMM40 | Yes | x | x |
| TOP1 | Yes | x | x |
| TOPBP1 | Yes | x | x |
| TOX4 | Yes | x | x |
| TP53 | Yes | x | x |
| TP53BP1 | Yes | x | x |
| TP73 | Yes | x | x |
| TPTEP2-CSNK1E | Yes | x | x |
| TRADD | Yes | x | x |
| TRAF2 | Yes | x | x |
| TRAF3 | Yes | x | x |
| TRAF5 | Yes | x | x |
| TRAPPC1 | Yes | x | x |
| TRAPPC10 | Yes | x | x |
| TRAPPC11 | Yes | x | x |
| TRAPPC12 | Yes | x | x |
| TRAPPC13 | Yes | x | x |
| TRAPPC2 | Yes | x | x |
| TRAPPC2L | Yes | x | x |
| TRAPPC3 | Yes | x | x |
| TRAPPC3L | Yes | x | x |
| TRAPPC4 | Yes | x | x |
| TRAPPC5 | Yes | x | x |
| TRAPPC6A | Yes | x | x |
| TRAPPC6B | Yes | x | x |
| TRAPPC8 | Yes | x | x |
| TRAPPC9 | Yes | x | x |
| TRAT1 | Yes | x | x |
| TREM2 | Yes | x | x |
| TRIM10 | x | Yes | x |
| TRIM14 | x | Yes | x |
| TRIM17 | x | Yes | x |
| TRIM2 | x | Yes | x |
| TRIM21 | x | Yes | x |
| TRIM22 | x | Yes | x |
| TRIM26 | x | Yes | x |
| TRIM29 | x | Yes | x |
| TRIM3 | x | Yes | x |
| TRIM31 | x | Yes | x |
| TRIM35 | x | Yes | x |
| TRIM38 | x | Yes | x |
| TRIM45 | x | Yes | x |
| TRIM46 | x | Yes | x |
| TRIM48 | x | Yes | x |
| TRIM5 | x | Yes | x |
| TRIM6 | x | Yes | x |
| TRIM62 | x | Yes | x |
| TRIM68 | x | Yes | x |
| TRIM8 | x | Yes | x |
| TRIOBP | x | Yes | x |
| TRIP10 | Yes | x | x |
| TRMT1 | Yes | x | x |
| TRMT44 | Yes | x | x |
| TRMT5 | Yes | x | x |
| TRUB1 | Yes | x | x |
| TSC1 | Yes | x | x |
| TSC2 | Yes | x | x |
| TSFM | Yes | x | x |
| TSHB | Yes | x | x |
| TSHR | Yes | x | x |
| TSPAN11 | x | Yes | x |
| TSR1 | Yes | x | x |
| TSR2 | Yes | x | x |
| TSR3 | Yes | x | x |
| TTC17 | Yes | x | x |
| TTI1 | Yes | x | x |
| TTLL6 | x | Yes | x |
| TTR | Yes | x | x |
| TUBB4B | Yes | x | x |
| TUFM | Yes | x | x |
| TXK | Yes | x | x |
| TXNDC5 | Yes | x | x |
| TYMS | Yes | x | x |
| TYROBP | Yes | x | x |
| U2AF1 | x | Yes | x |
| UBA2 | Yes | x | x |
| UBA5 | Yes | x | x |
| UBA52 | Yes | x | x |
| UBASH3A | Yes | x | x |
| UBASH3B | Yes | x | x |
| UBB | Yes | x | x |
| UBC | Yes | x | x |
| UBD | Yes | x | x |
| UBE2B | Yes | x | x |
| UBE2C | Yes | x | x |
| UBE2D1 | Yes | x | x |
| UBE2E1 | Yes | x | x |
| UBE2F | x | Yes | x |
| UBE2L6 | x | Yes | x |
| UBE2N | Yes | x | x |
| UBE2T | Yes | x | x |
| UBE2V2 | Yes | x | x |
| UBFD1 | Yes | x | x |
| UBL7 | Yes | x | x |
| UBQLN2 | Yes | x | x |
| UBR1 | Yes | x | x |
| UBR2 | Yes | x | x |
| UBR3 | Yes | x | x |
| UBR4 | Yes | x | x |
| UBR7 | Yes | x | x |
| UCHL5 | Yes | x | x |
| UCP3 | Yes | x | x |
| ULBP2 | Yes | x | x |
| ULK1 | Yes | x | x |
| ULK2 | Yes | x | x |
| UMPS | Yes | x | x |
| UNC13D | Yes | x | x |
| UQCR10 | Yes | x | x |
| UQCRB | Yes | x | x |
| UQCRC1 | Yes | x | x |
| UQCRC2 | Yes | x | x |
| UQCRFS1 | Yes | x | x |
| UQCRH | Yes | x | x |
| UQCRQ | Yes | x | x |
| URI1 | Yes | x | x |
| USE1 | Yes | x | x |
| USH1C | Yes | x | x |
| USH1G | Yes | x | x |
| USH2A | Yes | x | x |
| USHBP1 | Yes | x | x |
| USO1 | Yes | x | x |
| USP1 | Yes | x | x |
| USP10 | Yes | x | x |
| USP11 | Yes | x | x |
| USP14 | Yes | x | x |
| USP18 | x | Yes | x |
| USP19 | Yes | x | x |
| USP2 | Yes | x | x |
| USP28 | Yes | x | x |
| USP4 | Yes | x | x |
| USP43 | Yes | x | x |
| USP45 | Yes | x | x |
| USP8 | Yes | x | x |
| UST | Yes | x | x |
| UTP20 | Yes | x | x |
| UVRAG | Yes | x | x |
| UVSSA | Yes | x | x |
| UXT | Yes | x | x |
| VAMP1 | Yes | x | x |
| VAMP2 | Yes | x | x |
| VAMP3 | Yes | x | x |
| VAMP4 | Yes | x | x |
| VAMP7 | Yes | x | x |
| VAMP8 | Yes | x | x |
| VANGL1 | Yes | x | x |
| VANGL2 | Yes | x | x |
| VASP | Yes | x | x |
| VAT1 | Yes | x | x |
| VAV1 | Yes | x | x |
| VAV2 | Yes | x | x |
| VAV3 | Yes | x | x |
| VBP1 | Yes | x | x |
| VCAM1 | x | Yes | x |
| VCAN | Yes | x | x |
| VCL | Yes | x | x |
| VCP | Yes | x | x |
| VEGFA | Yes | x | x |
| VGF | Yes | x | x |
| VHL | Yes | x | x |
| VIPR1 | Yes | x | x |
| VLDLR | Yes | x | x |
| VNN1 | Yes | x | x |
| VNN2 | Yes | x | x |
| VNN3P | Yes | x | x |
| VPS51 | Yes | x | x |
| VPS52 | Yes | x | x |
| VPS53 | Yes | x | x |
| VPS54 | Yes | x | x |
| VSX2 | x | Yes | x |
| VTI1A | Yes | x | x |
| VTI1B | Yes | x | x |
| VTN | Yes | x | x |
| VWA1 | Yes | x | x |
| WAS | Yes | x | x |
| WASL | Yes | x | x |
| WDFY3 | Yes | x | x |
| WDFY4 | Yes | x | x |
| WDR11 | Yes | x | x |
| WDR20 | x | Yes | x |
| WDR26 | Yes | x | x |
| WDR31 | Yes | x | x |
| WDR36 | Yes | x | x |
| WDR48 | Yes | x | x |
| WDR54 | Yes | x | x |
| WDR72 | x | Yes | x |
| WFDC6 | x | Yes | x |
| WFS1 | Yes | x | x |
| WIPF3 | x | Yes | x |
| WIPI1 | Yes | x | x |
| WIPI2 | Yes | x | x |
| WNT1 | Yes | x | x |
| WNT11 | Yes | x | x |
| WNT3A | Yes | x | x |
| WNT4 | Yes | x | x |
| WNT5A | Yes | x | x |
| WNT5B | Yes | x | x |
| WNT8A | Yes | x | x |
| WNT8B | Yes | x | x |
| WRN | Yes | x | x |
| WTIP | Yes | x | x |
| WWOX | Yes | x | x |
| WWP1 | Yes | x | x |
| WWTR1 | Yes | x | x |
| XAB2 | Yes | x | x |
| XAF1 | x | Yes | x |
| XCR1 | Yes | x | x |
| XPA | Yes | x | x |
| XPC | Yes | x | x |
| XPNPEP2 | Yes | x | x |
| XPOT | Yes | x | x |
| XRCC1 | Yes | x | x |
| XRCC2 | Yes | x | x |
| XRCC3 | Yes | x | x |
| XRCC4 | Yes | x | x |
| XRCC5 | Yes | x | x |
| XRCC6 | Yes | x | x |
| XYLT1 | Yes | x | x |
| XYLT2 | Yes | x | x |
| YBX1 | Yes | x | x |
| YBX2 | Yes | x | x |
| YBX3 | Yes | x | x |
| YEATS4 | Yes | x | x |
| YKT6 | Yes | x | x |
| YPEL5 | Yes | x | x |
| YWHAB | Yes | x | x |
| YWHAG | Yes | x | x |
| YWHAZ | Yes | x | x |
| ZAP70 | Yes | x | x |
| ZBTB2 | x | Yes | x |
| ZBTB46 | x | Yes | x |
| ZNF365 | x | Yes | x |
| ZNF484 | x | Yes | x |
| ZNF550 | Yes | x | x |
| ZNF830 | Yes | x | x |
| ZNHIT3 | Yes | x | x |
| ZPBP | Yes | x | x |
| ZSWIM7 | Yes | x | x |
| ZSWIM8 | Yes | x | x |
| ZWINT | Yes | x | x |
| ZZEF1 | Yes | x | x |
| ADAM23 | Yes | Yes | Yes |
| ANAPC2 | Yes | Yes | Yes |
| ANK1 | Yes | Yes | Yes |
| ARPC3 | Yes | Yes | Yes |
| B2M | Yes | Yes | Yes |
| BLNK | Yes | Yes | Yes |
| CACNG3 | Yes | Yes | Yes |
| CD44 | Yes | Yes | Yes |
| CSGALNACT1 | Yes | Yes | Yes |
| CTSH | Yes | Yes | Yes |
| CXCL10 | Yes | Yes | Yes |
| DNM3 | Yes | Yes | Yes |
| FLNB | Yes | Yes | Yes |
| GABBR2 | Yes | Yes | Yes |
| GPIHBP1 | Yes | Yes | Yes |
| HECW1 | Yes | Yes | Yes |
| HLA-A | Yes | Yes | Yes |
| HLA-B | Yes | Yes | Yes |
| HLA-DPA1 | Yes | Yes | Yes |
| HLA-DPB1 | Yes | Yes | Yes |
| HLA-DQA2 | Yes | Yes | Yes |
| HLA-DQB1 | Yes | Yes | Yes |
| HLA-DRB1 | Yes | Yes | Yes |
| HLA-DRB5 | Yes | Yes | Yes |
| HLA-E | Yes | Yes | Yes |
| HLA-F | Yes | Yes | Yes |
| HLA-G | Yes | Yes | Yes |
| ICAM1 | Yes | Yes | Yes |
| ITGA11 | Yes | Yes | Yes |
| ITGA3 | Yes | Yes | Yes |
| ITGB2 | Yes | Yes | Yes |
| KIF26B | Yes | Yes | Yes |
| LYPD6B | Yes | Yes | Yes |
| MEPE | Yes | Yes | Yes |
| MGMT | Yes | Yes | Yes |
| MYO5A | Yes | Yes | Yes |
| NR3C2 | Yes | Yes | Yes |
| NSF | Yes | Yes | Yes |
| NTM | Yes | Yes | Yes |
| PDPN | Yes | Yes | Yes |
| PLCG1 | Yes | Yes | Yes |
| POLE | Yes | Yes | Yes |
| PRKAG2 | Yes | Yes | Yes |
| PSMB8 | Yes | Yes | Yes |
| PTH2R | Yes | Yes | Yes |
| SDHA | Yes | Yes | Yes |
| SGIP1 | Yes | Yes | Yes |
| SLC1A7 | Yes | Yes | Yes |
| SLC4A7 | Yes | Yes | Yes |
| TOP3A | Yes | Yes | Yes |
| TP53INP2 | Yes | Yes | Yes |
| TRIM25 | Yes | Yes | Yes |
| TSNARE1 | Yes | Yes | Yes |
| TUBB | Yes | Yes | Yes |
| USP15 | Yes | Yes | Yes |
| USP7 | Yes | Yes | Yes |
| VIPR2 | Yes | Yes | Yes |
