# Supplementary material for: Genome-wide DNA Methylation Profiling in Lyme Neuroborreliosis Reveals Altered Methylation Patterns of HLA Genes
Source: J Infect Dis. 2023 Oct 12;229(4):1209–14. doi: 10.1093/infdis/jiad451 (PMC11011177; doi:10.1093/infdis/jiad451)
Supplement: jiad451_Supplementary_Data [file jiad451_supplementary_data.zip › Table S5.docx]

| **Table S5.** KEGG pathway enrichment analysis of the genes derived from the consensus module. | | | | | |  |  |
| --- | --- | --- | --- | --- | --- | --- | --- |
| **ID** | **Description** | **GeneRatio** | **BgRatio** | **p.adjust** | **qvalue** | **geneID** | **Count** |
| hsa05416 | Viral myocarditis | 13/46 | 60/8292 | 0,00000000 | 0,00000000 | ICAM1/HLA-DQA2/HLA-DRB5/HLA-DPA1/HLA-F/HLA-B/HLA-E/HLA-DPB1/HLA-DQB1/ITGB2/HLA-A/HLA-DRB1/HLA-G | 13 |
| hsa05330 | Allograft rejection | 11/46 | 38/8292 | 0,00000000 | 0,00000000 | HLA-DQA2/HLA-DRB5/HLA-DPA1/HLA-F/HLA-B/HLA-E/HLA-DPB1/HLA-DQB1/HLA-A/HLA-DRB1/HLA-G | 11 |
| hsa05332 | Graft-versus-host disease | 11/46 | 42/8292 | 0,00000000 | 0,00000000 | HLA-DQA2/HLA-DRB5/HLA-DPA1/HLA-F/HLA-B/HLA-E/HLA-DPB1/HLA-DQB1/HLA-A/HLA-DRB1/HLA-G | 11 |
| hsa05169 | Epstein-Barr virus infection | 17/46 | 202/8292 | 0,00000000 | 0,00000000 | ICAM1/HLA-DQA2/HLA-DRB5/HLA-DPA1/CD44/B2M/HLA-F/HLA-B/HLA-E/CXCL10/BLNK/HLA-DPB1/HLA-DQB1/USP7/HLA-A/HLA-DRB1/HLA-G | 17 |
| hsa04940 | Type I diabetes mellitus | 11/46 | 43/8292 | 0,00000000 | 0,00000000 | HLA-DQA2/HLA-DRB5/HLA-DPA1/HLA-F/HLA-B/HLA-E/HLA-DPB1/HLA-DQB1/HLA-A/HLA-DRB1/HLA-G | 11 |
| hsa05320 | Autoimmune thyroid disease | 11/46 | 53/8292 | 0,00000000 | 0,00000000 | HLA-DQA2/HLA-DRB5/HLA-DPA1/HLA-F/HLA-B/HLA-E/HLA-DPB1/HLA-DQB1/HLA-A/HLA-DRB1/HLA-G | 11 |
| hsa04612 | Antigen processing and presentation | 12/46 | 78/8292 | 0,00000000 | 0,00000000 | HLA-DQA2/HLA-DRB5/HLA-DPA1/B2M/HLA-F/HLA-B/HLA-E/HLA-DPB1/HLA-DQB1/HLA-A/HLA-DRB1/HLA-G | 12 |
| hsa05166 | Human T-cell leukemia virus 1 infection | 15/46 | 222/8292 | 0,00000000 | 0,00000000 | ICAM1/HLA-DQA2/HLA-DRB5/HLA-DPA1/B2M/HLA-F/HLA-B/HLA-E/ANAPC2/HLA-DPB1/HLA-DQB1/ITGB2/HLA-A/HLA-DRB1/HLA-G | 15 |
| hsa04145 | Phagosome | 13/46 | 152/8292 | 0,00000000 | 0,00000000 | HLA-DQA2/HLA-DRB5/HLA-DPA1/HLA-F/HLA-B/HLA-E/HLA-DPB1/HLA-DQB1/ITGB2/HLA-A/TUBB/HLA-DRB1/HLA-G | 13 |
| hsa04514 | Cell adhesion molecules | 13/46 | 157/8292 | 0,00000000 | 0,00000000 | ICAM1/HLA-DQA2/HLA-DRB5/HLA-DPA1/HLA-F/HLA-B/HLA-E/HLA-DPB1/HLA-DQB1/ITGB2/HLA-A/HLA-DRB1/HLA-G | 13 |
| hsa05310 | Asthma | 6/46 | 31/8292 | 0,00000019 | 0,00000015 | HLA-DQA2/HLA-DRB5/HLA-DPA1/HLA-DPB1/HLA-DQB1/HLA-DRB1 | 6 |
| hsa05323 | Rheumatoid arthritis | 8/46 | 93/8292 | 0,00000044 | 0,00000034 | ICAM1/HLA-DQA2/HLA-DRB5/HLA-DPA1/HLA-DPB1/HLA-DQB1/ITGB2/HLA-DRB1 | 8 |
| hsa05150 | Staphylococcus aureus infection | 8/46 | 96/8292 | 0,00000052 | 0,00000040 | ICAM1/HLA-DQA2/HLA-DRB5/HLA-DPA1/HLA-DPB1/HLA-DQB1/ITGB2/HLA-DRB1 | 8 |
| hsa04640 | Hematopoietic cell lineage | 8/46 | 99/8292 | 0,00000061 | 0,00000047 | HLA-DQA2/HLA-DRB5/HLA-DPA1/CD44/ITGA3/HLA-DPB1/HLA-DQB1/HLA-DRB1 | 8 |
| hsa05140 | Leishmaniasis | 7/46 | 77/8292 | 0,00000185 | 0,00000143 | HLA-DQA2/HLA-DRB5/HLA-DPA1/HLA-DPB1/HLA-DQB1/ITGB2/HLA-DRB1 | 7 |
| hsa04672 | Intestinal immune network for IgA production | 6/46 | 49/8292 | 0,00000234 | 0,00000181 | HLA-DQA2/HLA-DRB5/HLA-DPA1/HLA-DPB1/HLA-DQB1/HLA-DRB1 | 6 |
| hsa05164 | Influenza A | 9/46 | 171/8292 | 0,00000283 | 0,00000219 | ICAM1/HLA-DQA2/HLA-DRB5/HLA-DPA1/TRIM25/CXCL10/HLA-DPB1/HLA-DQB1/HLA-DRB1 | 9 |
| hsa04658 | Th1 and Th2 cell differentiation | 7/46 | 92/8292 | 0,00000529 | 0,00000409 | HLA-DQA2/HLA-DRB5/HLA-DPA1/PLCG1/HLA-DPB1/HLA-DQB1/HLA-DRB1 | 7 |
| hsa05321 | Inflammatory bowel disease | 6/46 | 65/8292 | 0,00001089 | 0,00000841 | HLA-DQA2/HLA-DRB5/HLA-DPA1/HLA-DPB1/HLA-DQB1/HLA-DRB1 | 6 |
| hsa04659 | Th17 cell differentiation | 7/46 | 108/8292 | 0,00001419 | 0,00001096 | HLA-DQA2/HLA-DRB5/HLA-DPA1/PLCG1/HLA-DPB1/HLA-DQB1/HLA-DRB1 | 7 |
| hsa04650 | Natural killer cell mediated cytotoxicity | 7/46 | 132/8292 | 0,00005170 | 0,00003993 | ICAM1/PLCG1/HLA-B/HLA-E/ITGB2/HLA-A/HLA-G | 7 |
| hsa05145 | Toxoplasmosis | 6/46 | 112/8292 | 0,00022421 | 0,00017318 | HLA-DQA2/HLA-DRB5/HLA-DPA1/HLA-DPB1/HLA-DQB1/HLA-DRB1 | 6 |
| hsa05152 | Tuberculosis | 7/46 | 180/8292 | 0,00035427 | 0,00027363 | HLA-DQA2/HLA-DRB5/HLA-DPA1/HLA-DPB1/HLA-DQB1/ITGB2/HLA-DRB1 | 7 |
| hsa05167 | Kaposi sarcoma-associated herpesvirus infection | 7/46 | 194/8292 | 0,00054581 | 0,00042158 | ICAM1/PLCG1/HLA-F/HLA-B/HLA-E/HLA-A/HLA-G | 7 |
| hsa05322 | Systemic lupus erythematosus | 6/46 | 136/8292 | 0,00058662 | 0,00045310 | HLA-DQA2/HLA-DRB5/HLA-DPA1/HLA-DPB1/HLA-DQB1/HLA-DRB1 | 6 |
| hsa05170 | Human immunodeficiency virus 1 infection | 7/46 | 212/8292 | 0,00087824 | 0,00067834 | B2M/PLCG1/HLA-F/HLA-B/HLA-E/HLA-A/HLA-G | 7 |
| hsa04144 | Endocytosis | 7/46 | 251/8292 | 0,00238212 | 0,00183992 | HLA-F/HLA-B/HLA-E/ARPC3/HLA-A/HLA-G/DNM3 | 7 |
| hsa05203 | Viral carcinogenesis | 6/46 | 204/8292 | 0,00467041 | 0,00360736 | HLA-F/HLA-B/HLA-E/USP7/HLA-A/HLA-G | 6 |
| hsa05163 | Human cytomegalovirus infection | 6/46 | 225/8292 | 0,00749170 | 0,00578648 | B2M/HLA-F/HLA-B/HLA-E/HLA-A/HLA-G | 6 |
| hsa05410 | Hypertrophic cardiomyopathy | 4/46 | 90/8292 | 0,00767634 | 0,00592909 | ITGA3/ITGA11/PRKAG2/CACNG3 | 4 |
| hsa04218 | Cellular senescence | 5/46 | 156/8292 | 0,00807937 | 0,00624038 | HLA-F/HLA-B/HLA-E/HLA-A/HLA-G | 5 |
| hsa05165 | Human papillomavirus infection | 7/46 | 331/8292 | 0,01019753 | 0,00787642 | HLA-F/HLA-B/HLA-E/ITGA3/HLA-A/ITGA11/HLA-G | 7 |
| hsa04064 | NF-kappa B signaling pathway | 4/46 | 104/8292 | 0,01186865 | 0,00916717 | ICAM1/TRIM25/PLCG1/BLNK | 4 |
| hsa05412 | Arrhythmogenic right ventricular cardiomyopathy | 3/46 | 77/8292 | 0,03975250 | 0,03070425 | ITGA3/ITGA11/CACNG3 | 3 |
| hsa04721 | Synaptic vesicle cycle | 3/46 | 78/8292 | 0,04000716 | 0,03090095 | SLC1A7/NSF/DNM3 | 3 |
| hsa04512 | ECM-receptor interaction | 3/46 | 88/8292 | 0,05399006 | 0,04170114 | CD44/ITGA3/ITGA11 | 3 |
